# Non-Cognitive Specificities of Intellectually Gifted Children and Adolescents: A Systematic Review of the Literature

**DOI:** 10.3390/jintelligence11070141

**Published:** 2023-07-15

**Authors:** Emma Tourreix, Maud Besançon, Corentin Gonthier

**Affiliations:** 1 DysCo Lab, Paris Nanterre University, 92000 Nanterre, France; ejmtourreix@parisnanterre.fr; 2 LP3C, University of Rennes, 35000 Rennes, France; maud.besancon@univ-rennes2.fr; 3 Laboratoire de Psychologie des Pays de la Loire (LPPL UR 4638), Nantes Université, Chemin de la Censive du Tertre, 44312 Nantes, France

**Keywords:** intellectual giftedness, non-cognitive characteristics, children, adolescents

## Abstract

For several years, there was a growing interest in intellectual giftedness and in particular in the non-cognitive specificities of gifted individuals. This topic attracted much public attention and sometimes led to contradictions with the scientific literature. The current review synthesizes a broad set of results related to non-cognitive specificities of intellectual gifted in children and adolescents. This synthesis of scientific research on giftedness and its associated non-cognitive features does not support the conclusion that there is a stable profile across gifted individuals that would consistently separate them from non-gifted individuals. A few specificities in some areas are noted, but they are not necessarily being systematic. These specificities often turn out to be in favor of gifted youth, contrary to the view sometimes defended in the general public that gifted individuals suffer from major everyday difficulties. Finally, methodological issues are listed regarding the designs of existing studies, with recommendations for future research in the field.

## 1. Introduction

Because intellectually gifted individuals constitute a special population, and one that was not systematically studied, a great deal of information is still to be provided regarding related specificities. Many people have very different ideas about giftedness, including stereotypes or simply external perceptions that are not necessarily accurate, which can affect the way gifted youth are treated at school and in their everyday life (Barbier et al. 2022; Baudson 2016; Bergold et al. 2021; Carman 2011; Manaster et al. 1994; Subotnik et al. 2011; and Vialle et al. 2007).

Empirical studies support the conclusion that cognitive characteristics are associated with giftedness, such as high processing speed, effective representation of problems, flexibility in the choice of strategies and solutions, a broader knowledge base, etc. (Aubry et al. 2021; Calero et al. 2011; Geake 2008; Rodríguez Naveiras et al. 2019; and Steiner and Carr 2003). Nevertheless, it remains difficult to establish from the literature whether or not there are non-cognitive characteristics associated with giftedness. Indeed, despite the existence of a growing body of research interested in giftedness, no integrative literature review is available to synthesize findings about the non-cognitive specificities of gifted youth. However, public interest is largely focused on this non-cognitive profile of giftedness, which is often negatively connoted (Bergold et al. 2021). The implications of such a profile for clinical practice and education are very important. The daily lives, academic success, and well-being of gifted youth could be affected by these characteristics.

This article aims to fill this gap in the literature while confronting misconceptions about giftedness with available empirical findings. To prepare this review, we selected major topics regarding specificities associated with giftedness that come up frequently in discussions with laypeople and in media representations of giftedness. We only considered non-cognitive specificities in the sense that the topics covered here are not primarily related to cognitive ability as measured by a performance test; of course, this does not mean that the various topics discussed in this review are totally devoid of cognition (for example, emotional intelligence and humor are partly about cognitive processing). 

Because there is no universal criterion for identification (McBee and Makel 2019), it is important to be aware that the groups of gifted individuals identified in studies in the literature do not necessarily refer to a unique subpopulation of individuals (Carman 2013). In fact, the literature distinguishes several types of giftedness or even talent (Olszewski-Kubilius et al. 2016; Sternberg and Davidson 2005). Multidimensional models of giftedness and talent were proposed (e.g., Gagné 2005; Renzulli 2005). These models include cognitive variables, such as high intellectual ability or academic excellence, as well as non-cognitive variables, such as leadership, motivation, or talent. The major problem with these models is the lack of information on the specific functional relationships between their components, which hampers their use in empirical research and the interpretation of the resulting findings (Wirthwein et al. 2019; for further discussion of the implications of these different approaches for clinical practice, see McBee and Makel 2019). For this reason, we chose to focus this systematic review of the literature on a single component of giftedness: intellectual giftedness defined as high intellectual ability. This one-dimensional approach to giftedness is not unusual; in fact, it was the most prevalent approach in the literature for a long time (Carman 2013) and is still widely used today. Some authors may consider it a restrictive approach to giftedness. We believe that the criteria for including gifted groups should be different depending on whether we are talking about intellectual giftedness, creative giftedness, talent, or leadership. This distinction is essential from our point of view until we have more theoretical knowledge of how the different elements that make up multidimensional models of giftedness relate to each other, particularly because what applies to intellectual giftedness is not necessarily transposable to other types of giftedness. 

In many cases, more research was conducted regarding the correlation between a particular specificity and general intelligence than regarding the specific case of giftedness. In these instances, we first discuss results regarding correlations with intelligence in the general population before proceeding to studies involving gifted individuals. Moreover, very few studies focused on highly gifted individuals and their related characteristics. Definitions of high intellectual giftedness differ from one study to another: highly gifted youth are sometimes identified according to their academic performance (top 1% of the best students in the class versus 2.5% or 5% for gifted students), sometimes on the basis of a very high intelligence quotient (IQ ≥ 145, i.e., three standard deviations above the mean, versus IQ ≥ 130, i.e., two standard deviations above the mean for gifted youth). Where such studies are included in this literature review, this is mentioned in the body of the text. Details regarding the inclusion criteria for the highly gifted groups in each study are also provided in the summary tables appended.

## 2. Method

### 2.1. Study Search Procedure

In order to provide a comprehensive overview of the topics of interest, we conducted an exhaustive search in three stages. First, a search procedure in the PsycINFO, Eric Education, and PubMed databases was implemented. Next, we examined the reference lists of articles and reviews to identify any other relevant studies. Finally, an exploratory literature search was carried out using Google Scholar and its “cited by” function. 

To identify studies potentially eligible for inclusion in this literature review, we defined three sets of keywords. The first set aimed to restrict the literature to the gifted population using the following keywords: 

(*gifted* [Title/Abstract] OR *highly gifted* [Title/Abstract] OR *giftedness* [Title/Abstract] OR *high-IQ* [Title/Abstract] OR *high ability* [Title/Abstract]).

The second set of keywords intended to limit our research to literature on intelligence and academic achievement when appropriate: 

(*intelligence* [Title/Abstract] OR *IQ* [Title/Abstract] OR *cognitive ability* [Title/Abstract]) NOT (*emotional* [Title]) 

(*academic performance* [Title/Abstract] OR *achievement* [Title/Abstract] OR *school performance* [Title/Abstract]).

Finally, the third set of keywords varied according to each literature review section in order to tailor the results to the targeted research issues. Details of the sets used for each part of the literature review are presented in Table 1.

### 2.2. Inclusion and Exclusion Criteria

**Figure 1:**
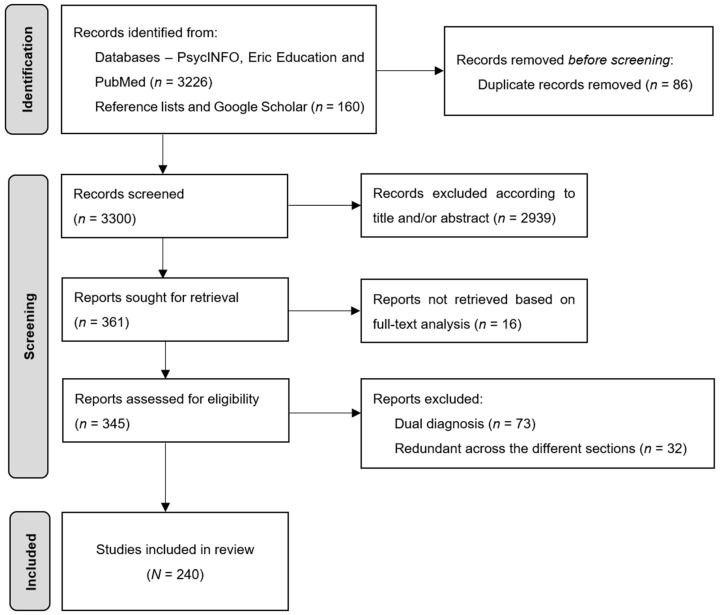
Flow chart describing the systematic review process.

Following this search procedure, we identified a total of 3386 studies across all sections. To limit our selection to only relevant articles for this literature review, inclusion and exclusion criteria were applied. With regard to the inclusion criteria, all studies included in this literature review were (a) published articles (b) written in English or French and (c) focusing on school age and/or adolescence (6–18 years, with the exception of correlational studies on general intelligence). Three restriction criteria were also applied, as described in Figure 1. Studies involving (a) populations with a dual diagnosis, including giftedness associated with a disorder (for example, attention-deficit/hyperactivity disorder ADHD, autism spectrum disorder ASD, or learning disabilities among the most common disorders in this field of the literature) were removed from the selection. The aim of this procedure was to limit as far as possible the effect of confounding variables on the results retained. Duplicate studies (b) were also systematically excluded. Finally, (c) studies deemed irrelevant according to their title, abstract, or a full-text analysis were removed from the final selection of articles. A total of 3114 articles were excluded following the application of these criteria (*n* = 73 studies excluded for double diagnosis, *n* = 86 excluded for duplication, *n* = 2939 studies excluded according to title and/or abstract, and *n* = 16 studies excluded because they were deemed irrelevant based on full-text analysis). A further 32 studies selected but redundant across the different sections were also removed, resulting in a final selection of *N* = 240 articles. Details on the final selection of articles included in this literature review are provided at the beginning of each section.

## 3. Integrative Review of the Literature

### 3.1. Anxiety

Are intellectually gifted children and adolescents more anxious than their non-gifted peers? This is the view shared by many people (Cross et al. 2008; Martin et al. 2010; Peyre et al. 2016; and Scholwinski and Reynolds 1985). For example, a survey conducted in France (Gauvrit 2014) from a Facebook group specialized in giftedness revealed a clear-cut opinion: intellectually gifted children are more anxious than their non-gifted peers according to 96% of the adult participants who responded to the survey. While public opinion seems unanimous, scientific research on the subject provides very different results (see Supplementary Table S1 for a summary of study methodologies and effect sizes on this topic). We first discuss anxiety in general, followed by test anxiety, and perfectionism.

#### 3.1.1. Anxiety in General

Total number of studies: *N* = 25 studies, including 3 meta-analyses, selected from 67 articles.

Anxiety is an emotion, characterized by apprehension and somatic symptoms of tension, in which an individual anticipates impending danger, catastrophe, or misfortune, as described by the American Psychological Association (2021). It is a normal and adaptive phenomenon present in every individual, but one that can become a problem when it appears in an excessive, pathological character. Studies examining anxiety in the intellectually gifted youth population focused primarily on the levels of non-pathological anxiety observed in participants.

Overall, the empirical studies report that intellectually gifted youth are not more anxious than their non-gifted peers (Beer 1991; Czeschlik and Rost 1994; Guénolé et al. 2013; and Pufal-Struzik 1999). For some authors, intellectually gifted youth have even lower anxiety levels than their non-gifted peers (Cernova 2005; Cross et al. 2008; Feldhusen and Klausmeier 1962; Milgram and Milgram 1976; Reynolds and Bradley 1983; and Zeidner and Shani-Zinovich 2011). In particular, a major study (Scholwinski and Reynolds 1985) conducted across the developmental span (6 to 19 years), including a large group of intellectually gifted youth identified as having a high IQ ≥ 130 (*N* = 584) as well as a large control group (*N* = 4923), found significantly lower scores on an anxiety scale in gifted than non-gifted individuals.

Three systematic reviews were conducted (Francis et al. 2015; Gauvrit 2014; and Martin et al. 2010) and led to the same conclusion that intellectually gifted youth are not more anxious than their non-gifted peers. In total, the four studies included in the analysis by Martin et al. (2010) reported a standardized mean difference of −0.72 (i.e., descriptively lower anxiety in gifted youth), with relatively comparable effect sizes across studies. No overall effect size was reported in the two other reviews, but 6 of 18 studies reviewed by Francis et al. (2015) and 12 of 13 studies reviewed by Gauvrit (2014) presented similar findings.

Some authors suggest that girls are more anxious than their male counterparts and that this pattern could be amplified by giftedness, but few studies present results in this direction. An example is Forsyth (1987), but this research suffered from major methodological limitations (the study design was based on a small sample of *n* = 42 gifted participants and the criteria used to define giftedness were not defined except for the fact that it was a combinatorial approach to intellectual and creative giftedness) and a lack of information to contextualize the findings (the article reports averages but no standard deviations, and the descriptive data are unspecified regarding group composition—participants’ age and grade level, or gender ratio in each group). To our knowledge, none of the other existing studies report such a gender effect for non-pathological anxiety in intellectually gifted youth. A few studies do, however, report an absence of a gender effect (Czeschlik and Rost 1994; Martin et al. 2010).

Pathological anxiety is a special case. On this topic, one landmark study in intellectually gifted youth (Guénolé et al. 2013) included the results of 106 participants aged 8 to 12 years, which were compared to normative data. The authors concluded that intellectually gifted children are no more likely to demonstrate pathological anxiety than their non-gifted peers on average. The authors also reported no significant gender effect in this study.

The concept of anxiety as a general construct includes multiple sub-components, such as worry and intolerance of uncertainty. These components, which are common to most anxiety disorders, are among the most reliable predictors of the development and maintenance of anxiety in an individual, especially in younger people (Kendall et al. 2020; Kertz et al. 2012; Osmanağaoğlu et al. 2018; Rabner et al. 2016; and Ryum et al. 2017). Although sub-dimensions of anxiety are frequently discussed in anxiety research, very little data are available regarding the study of anxiety in intellectually gifted young people. There are two major studies with conflicting results.

The first study (Peyre et al. 2016) provided results suggesting that intellectually gifted children tend to show excessive worry. However, this study was not specifically interested in worry and intolerance of uncertainty, and the authors warned of the need to replicate their findings. The second available study (Francis et al. 2018) investigated how intelligence (as measured with verbal, performance and full-scale IQ) was related to worry and intolerance of uncertainty in childhood and early adolescence. The results suggest that high intelligence, mediated by intolerance of uncertainty and threat appraisal, could be a protective rather than a risk factor for worry.

#### 3.1.2. The Case of Test Anxiety

Total number of studies: *N* = 6 studies, including 2 meta-analyses, selected from 19 articles.

Test anxiety refers to the anxiety felt specifically when faced with a skill and/or knowledge assessment situation in which the child has a vested interest in performing. Test anxiety and performance can reciprocally influence each other: on the one hand, high test anxiety before an exam multiplies the risks of performing poorly; on the other hand, dealing with recurrent failure situations increases the tendency to experience test anxiety when faced with an evaluation situation. Intellectually gifted young people could therefore be less vulnerable to this type of pattern insofar as they objectively have less difficulty achieving tasks on average.

Test anxiety in intellectually gifted youth is a topic particularly studied by Zeidner and Schleyer (1999), with results emphasizing that, in general, intellectually gifted youth experience less test anxiety than their non-gifted peers (in line with previous research: Beer 1991; Milgram and Milgram 1976). Studies about general intelligence, rather than giftedness, also support this conclusion: the meta-analyses by Hembree (1988) and Ackerman and Heggestad (1997) reported moderate negative correlations between the levels of test anxiety and intelligence scores (*r*s between −0.10 and −0.33; see Supplementary Table S2).

Even though intellectually gifted students tend to demonstrate lower test anxiety on average than their non-gifted peers across grade levels and gender groups (Zeidner and Schleyer 1999), particular patterns of anxiety can be observed for gifted youth who are enrolled in specialized education programs for the intellectually gifted. Two antagonistic effects can be observed in this situation. On the one hand, being in a situation where the performance of others is generally high—because of the high abilities of the reference group, a classroom composed of intellectually gifted peers—can lead to the development of a sense of incompetence through impairment of the student’s academic self-concept (Goetz et al. 2008). This phenomenon, where students have lower self-concepts as a result of comparing themselves to more able students, is summarized through the big-fish-little-pond effect (see Dai and Rinn 2008). On the other hand, being a member of a gifted group could have positive consequences on test anxiety in the context of the basking in reflected glory effect, which describes situations in which students are valued by belonging to a group that is positively recognized. Some students may increase their academic self-image because they recognize themselves as already competent/intelligent enough to be enrolled in these specialized classes for intellectually gifted youth. In such cases, test anxiety may decrease (Goetz et al. 2008).

#### 3.1.3. Perfectionism and Intellectual Giftedness

Total number of studies: *N* = 8 studies, including 8 meta-analyses, selected from 75 articles.

Perfectionism is commonly perceived as a personality disposition of individuals with intellectual giftedness, and it is part of several theoretical views of giftedness. Yet a recent study suggests that perfectionism may not be significantly associated with giftedness (Yi and Gentry 2021). Perfectionism is mostly studied according to a two-dimensional approach (Bieling et al. 2004), including perfectionistic concerns and perfectionistic strivings. Perfectionistic concerns are maladaptive, and they are linked to the various known indicators of psychological and behavioral maladjustment (e.g., fear of negative social evaluations or making mistakes, avoidance, discrepancy between expectations and performances in Hill and Curran 2015). By contrast, perfectionistic strivings are associated with desirable adaptative behaviors and positive outcomes as motivational clues (Sirois et al. 2017), such as pleasure during efforts and the setting of challenging goals inside and outside of educational contexts (Chan 2008).

Empirical results regarding perfectionism are highly heterogeneous but were summarized in two landmark meta-analyses (Stricker et al. 2019; Ogurlu 2020). Considering perfectionism in general, gifted youth appear to demonstrate levels of perfectionism similar to non-gifted peers (overall effect of *g* = 0.06 in Ogurlu 2020; the *g* effect size can be read as a corrected Cohen’s *d*; and Stornelli et al. 2009), and they seem to experience perfectionism in a constructive way (Parker 2000; Schuler 2000). In the case of perfectionistic concerns, the meta-analyses by Stricker et al. (2019) and Ogurlu (2020) share a common view, suggesting that intellectually gifted youth do not experience a higher level of maladaptive perfectionistic preoccupation than their non-gifted peers (non-significant differences in both meta-analyses, with *g* = −0.12 in Stricker et al. 2019 and *g* = −0.13 in Ogurlu 2020). Indeed, for some studies included in these meta-analyses, gifted youth may even exhibit less maladjusted perfectionism than their peers (Chan 2010; Kornblum and Ainley 2005; LoCicero and Ashby 2000; Parker et al. 2001). As for perfectionistic strivings, both meta-analyses suggest that intellectually gifted youth demonstrate descriptively more perfectionistic strivings than their peers, but the difference was only significant in Stricker et al. (2019) (*g* = 0.33 and *g* = 0.19, respectively; for a summary, see Supplementary Table S3). 

A few studies, published (Guignard et al. 2012; Roberts and Lovett 1994) or unpublished (Bull 1997; Reser 2016; and Vuyk 2010), never considered perfectionism under a multidimensional view centered around perfectionistic concerns and strivings, and were instead based on a distinction between perfectionism oriented towards oneself (“requirements imposed by the individual on his/herself for him/her to be perfect”) and perfectionism as socially prescribed (“requirements perceived by the individual that others require him/her to be perfect” in Affrunti and Woodruff-Borden 2014). While self-oriented perfectionism can be conceptually related to the high personal standards and motivational aspects that define perfectionistic striving, there is also a negative side to self-oriented perfectionism, including a tendency to self-blame and associated depressive and anxiety affects (Hewitt and Flett 1991). This negative facet is absent from the conception of perfectionistic striving and is obscured in the meta-analyses by Stricker et al. (2019) and Ogurlu (2020). This partly contributes to confusion surrounding the results: in particular, two studies (Guignard et al. 2012; Roberts and Lovett 1994) found higher levels of self-oriented perfectionism in gifted than in their non-gifted peers in some settings, which partly converges with the findings of meta-analyses regarding perfectionistic striving; on the other hand, in both studies, self-oriented perfectionism was associated with worry and negative affects rather than with the benefits of perfectionistic striving. Although these results could hint at more anxiety-related perfectionism in gifted youth, they concern very specific contexts: intellectually gifted youth displayed a higher level of self-oriented perfectionism than their peers only in the 6th grade in the study of Guignard et al. (2012), and only in dealing with failure in the school context in the study of Roberts and Lovett (1994).

In sum, the results observed in the literature do not provide evidence of a positive correlation between intellectual giftedness and anxiety, either pathological or non-pathological, when considering general or specific measures of anxiety and its components. The literature provides similar findings about test anxiety and dimensions of perfectionism.

### 3.2. Mood Disorders

#### 3.2.1. Depression in Intellectually Gifted Youth

Total number of studies: *N* = 8 studies, including 2 meta-analyses, selected from 57 articles.

Over the past few years, several reviews of the literature were dedicated to the examination of depression in intellectually gifted children and adolescents (Cross et al. 2008; Cross and Cross 2015; Francis et al. 2015; Martin et al. 2010; and Missett 2013). All these reviews provide almost unanimous results, despite major heterogeneity in the methodologies used to study depression: intellectually gifted youth are not any more at risk for developing depressive disorder than their peers (see Supplementary Table S4). For example, out of six studies devoted to depression in intellectually gifted youth as reported in Martin et al. (2010), five agreed that there was no significant difference between the depression scores observed in intellectually gifted youth and a reference group (*d* = −0.17). The sixth study (Bénony et al. 2007) reported contradictory results, with higher depression scores in the group of intellectually gifted participants compared to the control group, as linked to low self-esteem levels, but the results are based on small samples of children (*n* = 23 in each group). Some authors (Francis et al. 2015) suggest that intellectually gifted youth could have even lower depression scores than their peers.

One possibility is that the results could be different at the extreme range of intellectual giftedness, i.e., in highly gifted individuals. A single experimental study (Baker 1995) compared intellectually gifted, highly intellectually gifted, and non-gifted youth on a depression scale. The author found no differences between the groups. Nevertheless, this study suffered from small sample sizes (with *n* = 56 controls, *n* = 32 highly gifted participants, and *n* = 58 gifted participants) and gifted selection based on academic performance (SAT scores of the highly gifted and gifted participants corresponding to the top 1% and 5% of students of age 13, respectively; all of the highly gifted students and some of the gifted students (about 49% of the participants in both groups) were in a special program for the gifted and talented). A more recent multiple case study (Jackson and Peterson 2003) also focused on the specific case of depression among highly intellectually gifted youth. The opposite results were obtained, suggesting that highly intellectually gifted youth may be more likely to be depressed than their peers, both intellectually gifted and non-gifted.

In sum, there is a lack of data on depression among the highly gifted individuals, so no conclusions can be drawn. Nevertheless, it seems important to keep in mind that counselors and psychologists working with highly gifted youth may be led to believe that they are more vulnerable to depression than their gifted and non-gifted peers (Mueller and Winsor 2018). Additional research is needed to support these clinical findings (Wood and Laycraft 2020).

#### 3.2.2. Suicidal Ideation and Intellectual Giftedness

Total number of studies: *N* = 3 studies, including no meta-analyses, selected from 11 articles.

In line with research on depression, some authors examined the relationship between suicidal ideation and intellectual giftedness (see Supplementary Table S5). Martin et al. (2010) report two studies addressing this issue, including comparison groups of non-gifted participants. A first study (Baker 1995) compared intellectually gifted, highly intellectually gifted, and non-gifted youth (grades 9 through 11); the second study (Metha and McWhirter 1997) focused on a group of intellectually gifted youth and a group of non-gifted youth (grades 7 and 8). The results of these two studies were similar; there were no significant differences between the respective groups. This was more recently corroborated by Cross et al. (2006), also in intellectually gifted adolescents.

There are still few studies examining suicidal ideation in intellectually gifted youth compared to their reference group. The few empirical works that were conducted on the subject too often include samples that are not representative of the population of interest, and are usually very small-scale studies (Cross and Cross 2015). This lack of empirical evidence leads to a “tendency for authors to make conclusions and recommendations about the incidence of suicide without supporting data”, as noted by Cross (1996).

#### 3.2.3. Other Mood Disorders and Intellectual Giftedness

Total number of studies: *N* = 5 studies, including no meta-analyses, selected from 24 articles.

Other studies on giftedness used general measures of mental health difficulties and found small to moderate differences between gifted and non-gifted participants, always in favor of gifted participants (*d* = 0.59, *p* < .05 for participants aged 11 in Cook et al. 2020; and effect sizes ranging from *d* = 0.32 to *d* = 0.76 in Cross et al. 2008).

Although not specifically focused on gifted individuals, it is worth mentioning here two longitudinal studies that examined the relation between intelligence and mental health (Koenen et al. 2009; MacCabe et al. 2010). The results suggest that people with high abilities are significantly more at risk of developing a bipolar disorder in adulthood compared to people with average abilities (*d* = 1.28 in Koenen et al. 2009). The opposite trend was observed for all other mental disorders, including schizophrenia, anxiety, and depressive disorders, with high abilities seeming to act as a protective factor. These findings need to be treated with caution because neither study directly addressed the population of intellectually gifted youth (see Supplementary Table S6). The first study (Koenen et al. 2009), based on a 1972–1973 birth cohort from New Zealand, defined participants as having high abilities based on an IQ > 115; the second study (MacCabe et al. 2010), investigating data from all children completing compulsory schooling in Sweden between 1988 and 1997, defined participants as high achievers when they obtained a grade of A (top 7% of cohort members) on a national exam. These criteria are much less stringent than the usual threshold of an IQ of 130 or more used to define intellectual giftedness, which corresponds to the IQs obtained by 2.5% of the population.

Overall, existing empirical works do not support the hypothesis that intellectually gifted children and adolescents have specific mental health characteristics that could be risk factors for developing psychopathological disorders (Stricker et al. 2019). High achievers may conceivably be more vulnerable to the development of bipolar disorder in adulthood, but no study confirmed this result in intellectually gifted participants per se. Furthermore, prior works generally suffered from a lack of concern about the precise nature of the disorder (e.g., type I and type II bipolarity; see Missett 2013). This field of study thus requires further investigation to ensure reliable findings in the future.

### 3.3. Well-Being and Quality of Life

#### 3.3.1. Objective Indicators of Achievement and Quality of Life: General Intelligence

Total number of studies: *N* = 10 studies, including 1 meta-analyses, selected from 908 articles.

A first set of studies investigated the general correlation between IQ scores and achievement. Many authors identified, with stable results over time and across studies, that high cognitive ability was strongly associated with high academic performance (Deary et al. 2007; Demetriou et al. 2020; Guez et al. 2018; Li and Shi 2019; Neisser et al. 1996; and Watkins et al. 2007). A high IQ is also predictive of a higher number of years of schooling than average, a high social status, high work performance in adulthood, and a higher average income (Ceci and Williams 1997; Neisser et al. 1996; Ree and Earles 1992; and Strenze 2007). On average, individuals with high IQs are therefore objectively more likely than their peers to experience success and achievement in their lives, both at school and at work. In addition, and related to these different elements, correlations were observed between intelligence scores and life expectancy (Deary et al. 2008). Among other things, people with high cognitive skills are less likely to develop medical disorders during their lifetime, regarding both physical health and mental health (see above).

#### 3.3.2. Objective Indicators of Achievement and Quality of Life: Specific Case of Giftedness

Total number of studies: *N* = 3 studies, including no meta-analyses, selected from 360 articles.

Based on objective indicators of achievement and quality of life, empirical results suggest that high cognitive abilities act as a protective factor, predicting positive outcomes throughout life (see Supplementary Table S7). Several recent studies conducted specifically among samples of intellectually gifted children and adolescents (Bergold et al. 2020; Eklund et al. 2015; and Wirthwein et al. 2019) confirm these findings regarding school achievement. These results suggest that gifted youth are on average more likely to experience a situation of academic success than failure, although this is not systematically true for all individuals. To our knowledge, there are no major studies regarding objective measures of quality of life (apart from academic achievement) in gifted youth, making this an open area for future research.

#### 3.3.3. Life Satisfaction and Subjective Well-Being: General Intelligence

Total number of studies: *N* = 4 studies, including 1 meta-analyses, selected from 161 articles.

Empirical studies conducted to date suggest that there is a very weak relationship between general cognitive ability scores and levels of life satisfaction and subjective well-being in children and adolescents as well as adults (*r* = −0.08 in Huebner and Alderman 1993; *β* = 0.04 in Chmiel et al. 2012; and *r* = 0.02 in Cheng and Furnham 2014). A recent meta-analysis (Bücker et al. 2018) also highlighted a small, but statistically significant, correlation (*r* ≃ 0.16) between subjective well-being and academic achievement, which certain authors view as a proxy for intellectual abilities.

#### 3.3.4. Life Satisfaction and Subjective Well-Being: Specific Case of Giftedness

Total number of studies: *N* = 10 studies, including 1 meta-analyses, selected from 56 articles.

A few studies including control groups (Ash and Huebner 1998; Bergold et al. 2015; Shaunessy et al. 2006; and Yong and McIntyre 1991) focused on the level of life satisfaction of intellectually gifted children and adolescents, as reported in Zeidner (2021) meta-analysis. The results are consistent: there is no difference on average between the life satisfaction levels of gifted and non-gifted youth (summary effect *g* = −0.01, interpretable as a Cohen’s *d* in Zeidner 2021), except for one study (Shaunessy et al. 2006) that reported significantly higher life satisfaction scores among the gifted group than the control group (*d* = 0.39). However, these studies were mostly based on small samples of both gifted and non-gifted participants, which may limit the generalizability of the findings (see Supplementary Table S8).

One of these studies (Bergold et al. 2015) is of particular interest because it relied on a robust methodological design, including a large control group (*n* = 580) and a gifted group that was comparatively smaller (*n* = 75) but recruited in line with recommendations (i.e., top 2.5% of intelligence scores). An important feature of this study is that the gifted participants were identified based on testing after inclusion in the study, and participants were not made aware of their scores, thus preventing any labeling effect. The authors found no significant difference between the life satisfaction reported by intellectually gifted and non-gifted adolescents (*d* = −0.03). Despite a valuable methodological design, this result may be partly due to an unusual sampling, with all participants coming from a selective educational curriculum, which is not necessarily representative of the general population. Recently, a study investigating the school life satisfaction of gifted youth (*n* = 66 students labeled as gifted and *n* = 362 non-gifted students in Guignard et al. 2021) reached similar conclusions, namely that gifted youth do not differ from their non-gifted peers.

Apart from life satisfaction, a few studies were interested more specifically in subjective well-being. This line of research yielded very different outcomes. Some results indicate no group differences (Bergold et al. 2020; Yong and McIntyre 1991), others note lower scores of subjective well-being in intellectually gifted children compared to their non-gifted peers (Casino-García et al. 2019; Zeidner and Shani-Zinovich 2011), and yet others report significantly higher levels of general subjective well-being in gifted children than their peers (Pontes de France-Freitas et al. 2019). It is difficult to reconcile these results, given the small number of studies on this subject and given their discrepancy (*g* = −0.54 to 0.39 in Zeidner’s meta-analysis (Zeidner 2021).

In summary, while intellectual giftedness is positively correlated with academic achievement, no clear-cut conclusions can be drawn regarding the objectively assessed quality of life of gifted youth in particular. Nevertheless, there is evidence of general positive correlations between IQ, life expectancy, and physical health. Furthermore, the results consistently show no difference on average between gifted and non-gifted youth in terms of life satisfaction, but the findings regarding the subjective well-being of gifted youth relative to their peers are inconsistent. This discrepancy between, on the one hand, objective indicators of quality of life tending to be higher among gifted youth, and on the other hand, neutral or unstable findings regarding subjective indicators of life satisfaction and well-being, highlights why it is important to consider the experience of being gifted beyond skills.

### 3.4. Social Relationships and Intellectual Giftedness

#### 3.4.1. Social Abilities

Total number of studies: *N* = 30 studies, including no meta-analyses, selected from 67 articles.

Shared results with perceived socialization.

One line of research into the social abilities of gifted youth was interested in social coping, and the literature identified evidence of adaptive social coping abilities in intellectually gifted children and adolescents (Lehman and Erdwins 1981). There appear to be coping strategies that are specific to giftedness (Swiatek 1995, 2001), such as denying giftedness; reducing the impact of giftedness on peer acceptance; hiding giftedness or conforming to peers’ behaviors; denying importance of popularity; using humor; helping others; and engaging in social interactions and a high activity level to keep busy and share common interests. These social coping strategies may vary depending on gender, social contexts, and especially the school environment. Culture also seems to have an impact on the preferential use of certain social coping strategies over others in the case of giftedness (see Cross and Swiatek 2009). For example, hiding conformity is a social coping strategy widely used by gifted students in the United States and some European countries, whereas this is not the case in South Korea where it is socially valued to show the extent of one’s abilities.

Intellectually gifted children and adolescents are also reported as being motivated to maintain positive relationships with their peers and teachers, and as actively avoiding conflict (França-Freitas et al. 2014; Peairs et al. 2019; and Richards et al. 2003) although this is not a coping strategy identified by Swiatek (1995, 2001). The development of these social coping strategies would be in response to the particular life experience of gifted youth and would be explained in part by strong cognitive abilities that allow for the effective implementation of processes involved in successful socialization, such as perspective taking and problem solving (Peairs et al. 2019).

Another issue addressed by research in intellectually gifted individuals, especially gifted adolescents, is victimization and bullying. For example, one study (Ryoo et al. 2017) reported, on average, no significant differences between intellectually gifted adolescents and their non-gifted peers in terms of their history of victimization. Many authors also concluded that intellectually gifted children are more autonomous, independent, and self-sufficient in daily life than their peers of the same age, although they enjoy playing with others, including older children (Janos and Robinson 1985; Lehman and Erdwins 1981; and Richards et al. 2003). Gifted students also perceived themselves as being more intimate with their best friends than nongifted peers and reported being closer to their friends than to family (Field et al. 1998).

Two open issues emerge from this field of literature. First, there is the question of whether there are differences between intellectually gifted children and intellectually gifted adolescents. According to Mouchiroud (2004), intellectually gifted children report on average being closer to their friends than peers of the same age, but this result was not replicated among intellectually gifted junior high school students, who reported being less satisfied with their social support than peers of the same age (i.e., poorer quality of friendships—Masden et al. 2015; Vialle et al. 2007). The same trend was observed by a number of researchers. Socialization processes evolve during development, as do social norms and social expectations; the experience of having intellectual giftedness, or rather the experience of being labeled as gifted, can therefore change over time. In particular, intellectually gifted young people may experience more social difficulties during adolescence (Peairs et al. 2019; Robinson 2008; and Shechtman and Silektor 2012).

The literature suggests that this evolving life experience can affect the self-concept of gifted youth. One study (Milgram and Milgram 1976) observed that intellectually gifted children demonstrate higher degrees of personal worth and self-confidence (except for body image) than their peers in grades 4 to 6, but lower scores in grades 7 to 8 on several self-concept measures. Another study (Zeidner and Shani-Zinovich 2015) found that intellectually gifted adolescents reported less favorable social (partial η^2^ = 0.02, *p* < .001), personal (partial η^2^ = 0.01, *p* < .001), and physical self-concepts (partial η^2^ = 0.01, *p* < .01) than their peers in grades 10 to 12. However, these findings on gifted evolutionary life experience and self-concept are not systematic. For example, one study (Shechtman and Silektor 2012) including gifted students from segregated classrooms and pull-out programs, as well as non-gifted students, all from grades 5 to 12, found that self-concept scores did not depend on grade level, and were similar overall across all groups of participants for various self-concept facets (no significant difference between the groups on total self-concept scale scores). Nevertheless, some differences remain as all gifted youth reported higher levels of academic self-concept than their non-gifted peers, but lower physical self-concept on average. Social self-concept did not differ between groups.

A second recurring issue is the socialization of highly intellectually gifted children and adolescents. There is a distinct lack of consensus in the literature regarding youth with very high abilities. For some authors, very high intellectual abilities are associated with a lower degree of popularity, or even with social difficulties (Brody and Benbow 1986; Dauber and Benbow 1990; and Robinson 2008). Hollingworth (1942) proposed the concept of “socially optimal intelligence”, suggesting that there is a sort of optimal threshold: too high cognitive skills could turn into a disadvantage rather than an advantage for effective social processes. For instance, a very high intelligence could deepen gaps between highly intellectually gifted youth and their non-gifted peers in terms of common interests, social goals, and so on.

Other authors, however, never supported this conclusion (Gallucci 1988; Garland and Zigler 1999; and Norman et al. 1999) or offered nuanced findings. For example, Janos and Robinson (1985) reported that, overall, the social adjustment of highly intellectually gifted students did not differ from those of their moderately gifted peers. Only a minority of highly intellectually gifted youth appeared to experience substantial difficulties to make friends, although this minority might be somewhat larger than in youth with average and moderate high IQs.

#### 3.4.2. Perceived Socialization

Total number of studies: *N* = 30 studies, including no meta-analyses, selected from 67 articles.

Shared results with Social skills.

Although intellectually gifted children and adolescents do not appear to face more social difficulties than their non-gifted peers on average, they themselves report experiencing pressure from being assigned socially valuable characteristics by others due to their intellectual giftedness. For example, intellectually gifted people are often viewed as “weird” (Coleman and Cross 2014). This suggests that it can be valuable to investigate how others perceive social skills associated to giftedness.

##### Perception by Adults: Parents and Teachers

Available empirical evidence yields homogeneous observations regarding how gifted youth are perceived by adults. A large number of studies reported that intellectually gifted children and adolescents exhibit similar or better emotional and adaptive behavioral functioning than their peers, according to parents (Cook et al. 2020; Czeschlik and Rost 1994; Eklund et al. 2015; Galloway and Porath 1997; Garland and Zigler 1999; Peyre et al. 2016; and Richards et al. 2003). One study (Simoes Loureiro et al. 2010) presented contrary results based on interviews with parents of gifted and non-gifted children, but the sample of gifted children was based on a clinical population (children engaged in psychological follow-up) not representative of gifted youth in general.

The same results are found when the scales are filled in by teachers: both children and intellectually gifted adolescents are described as having similar or better functioning than their peers (Czeschlik and Rost 1994; Eklund et al. 2015; Field et al. 1998; Gallucci 1988; Gallucci et al. 1999; and Vialle et al. 2007). Gifted children and adolescents were also perceived by their teachers to be as (Bain and Bell 2004) or more (Košir et al. 2015; partial η^2^ = 0.02, *p* < 0.002) socially accepted by their classmates than their non-gifted peers. Another study (Peairs et al. 2019) expanded on this work and reported that intellectually gifted adolescents were perceived by their teachers as engaging in less bullying behavior and victimization than their peers.

Research on the perception of gifted youth by their teachers and its impact on socialization yielded a surprising finding: one study (Field et al. 1998) reported that “teachers rated the gifted students as being less happy [with intellectual giftedness] than the students rated themselves”. These results presumably reflect part of the social issues related to preconceptions about giftedness. This idea is described by Coleman and Cross (1988, 2014) as the “stigma of giftedness”. These authors detail three categories of strategies (visible coping, desertification, and/or camouflage strategies) used by gifted students to manage the information that others have about their giftedness in order to avoid this stigma.

##### Perception by Peers

Asking young people about which students they like most and least in their school or classroom, the results reveal that intellectually gifted children and adolescents are on average named equally often or more often than their non-gifted peers among the most well-liked students (Cohen et al. 1994; Košir et al. 2015; López and Sotillo 2009; and Peairs et al. 2019). These findings were reported in a situation of classic schooling (e.g., Peairs et al. 2019), and in a situation of participation in a pull-out enrichment program (e.g., Cohen et al. 1994). In these same studies, intellectually gifted young people (children in Cohen et al. 1994, Košir et al. 2015, and López and Sotillo 2009; adolescents in López and Sotillo 2009 and Peairs et al. 2019) were also categorized by their peers more often as “popular” than as “rejected”. Cohen et al. (1994) also reported good reciprocity in the friendships of intellectually gifted children, and there was a tendency reported by non-gifted peers for gifted children to be less victimized than their peers. Peairs et al. (2019) found no significant difference between victimization rates of intellectually gifted and non-gifted adolescents, as reported by non-gifted students. Furthermore, intellectually gifted adolescents are perceived by their peers as being less aggressive than others (Cohen et al. 1994; Peairs et al. 2019), and as engaging in more prosocial and cooperative behaviors (Peairs et al. 2019).

In summary, studies do not support the idea that intellectually gifted children and adolescents are less socially adjusted on average than their peers—whether according to peers, teachers, parents, or the intellectually gifted youth themselves (see Supplementary Table S9). This does not mean that intellectually gifted children and adolescents are fully protected from bullying or social isolation, but they do not appear to be particularly prone to such experiences as a group. Still, it is important to be aware of social issues in intellectually gifted children and adolescents, as possible suffering may be hidden behind internalized behaviors and high academic performance (Peairs et al. 2019).

### 3.5. Self-Esteem

One of the main sources of misconceptions about giftedness is how gifted youth perceive themselves. The literature covers a wide range of terms used to refer to self-perceptions, creating uncertainty in the conclusions drawn (Hansford and Hattie 1982). In this section, we focus on self-esteem, defined as a value judgment made by the individual about himself or herself (i.e., how satisfied I am with myself, how competent, valuable, worthy, etc.). This evaluative self-perception construct is to be distinguished from self-concept, which relates to a descriptive dimension of self-perception (Battle and Blowers 1982; Blyth and Traeger 1983; Courtinat-Camps et al. 2012; Foley-Nicpon et al. 2012; Harter 1993; del Mar Ferradás et al. 2020; and Orth and Robins 2022). In line with this evaluative component, self-esteem would strongly depend on feedback from significant others about oneself, even if implicit, as well as on the background of life experiences (Baumeister 1997; Casino-García et al. 2021; Coopersmith 1967; and Filosa et al. 2022). For children and adolescents, life experiences mainly refer to successes and failures in the school and social spheres. However, it is above all the importance attached to events that emerges as a key factor in self-esteem development (Preckel et al. 2016; Vialle et al. 2005). To illustrate, a child with poor coordination may be regularly challenged in sports. He/she may be cognitively able to report a description of his/her level of performance; this is a sub-part of the self-concept. He/she may also have a poor idea of his/her ability to do sport, based on previous experiences and feedback from friends or coach; this is self-esteem. However, if he/she is not committed to sport, self-esteem will not be affected (Vialle et al. 2005).

Since self-esteem is closely linked to feedback from others and past experiences, the question of self-esteem among gifted youth is particularly relevant. On the one hand, stereotypes associated with giftedness (Wirthwein et al. 2019) and the label effect that results in a negative feeling of being different for some gifted youth (Anderson 2020; Freeman 2013; Janos et al. 1985; Pérez et al. 2020; and Zschaler 2019) could make the gifted more vulnerable as a group when it comes to self-esteem. On the other hand, gifted youth were shown to perform better or as well as their non-gifted peers in many fields, including the intellectual and academic sphere, as well as the social sphere (cf. Objective indicators of achievement and quality of life and Social relationships and giftedness). In fact, one study (Preckel et al. 2016) suggests that gifted youth may value intellectual ability more than academic achievement in their self-esteem. From this point of view, most gifted youth should have rather high self-esteem compared to their non-gifted peers (Chiu 1990; Foley-Nicpon et al. 2012). The risk according to popular belief would ultimately be that gifted youth have self-esteem that is too high and that they tend to be arrogant (Barbier et al. 2022; Sekowski 1995; and Subotnik et al. 2011). 

#### 3.5.1. Self-Esteem: General Intelligence

Total number of studies: *N* = 32 studies, including 1 meta-analyses, selected from 903 articles.

Self-esteem was examined through its relationship with both intelligence and achievement. With regard to general intelligence first, the literature reports a wide range of correlations, from no relationship between intelligence and self-esteem (e.g., Kaya and Ogurlu 2015) to a correlation close to *r* = 0.30 (e.g., Simon and Simon 1975, only for verbal IQ). Overall, the literature is consistent with a weak positive relationship between intellectual ability and self-esteem (Coopersmith 1967; Lewis and Adank 1975; Papadopoulos 2021; Roznowski et al. 2000; and Simon and Simon 1975). However, a high level of intelligence would not be predictive of a high level of self-esteem, whereas a low level of intelligence would be predictive of a low level of self-esteem. Indeed, some studies report a curvilinear relationship between self-esteem and IQ (Asendorpf and van Aken 1994; Trowbridge 1974), which may partly explain why the Pearson correlations observed tend to be small. 

As a high IQ is strongly associated with high academic performance and the literature is extensive on the relationship between achievement and self-esteem, we thought it appropriate to summarize findings in this section, although not directly related to intelligence. In fact, the literature offers a conclusion quite similar to that observed just above, namely that there would be a weak positive correlation (Alsaker 1989; Baumeister et al. 2003; Coopersmith 1967; Di Giunta et al. 2013; Diseth et al. 2014; Ghobary and Hejazi 2007; Giofrè et al. 2017; Hansford and Hattie 1982; Lewis and Adank 1975; Marsh and Craven 2006; Moyano et al. 2020; Papadopoulos 2021; Pullmann and Allik 2008; Rosenberg et al. 1989; Ross and Broh 2000; Schmidt and Padilla 2003; Simon and Simon 1975; Tetzner et al. 2017; Topçu and Leana-Taşcılar 2018; and Yang et al. 2019), if not zero (D’Amico and Cardaci 2003; Kaya and Ogurlu 2015; Marsh and O’Mara 2008; Pullmann and Allik 2008; Schmidt and Padilla 2003; Vialle et al. 2005; and Zuffianò et al. 2013), between achievement and self-esteem. Authors interested in the direction of this relationship suggest that there is a reciprocal or recursive causal relationship between achievement and self-esteem, and that this may vary according to school level (see Liu et al. 1992; Pottebaum et al. 1986; Roskam and Nils 2007; and Skaalvik and Hagtvet 1990 for more information).

#### 3.5.2. Self-Esteem: Specific Case of Giftedness

Total number of studies: *N* = 32 studies, including 1 meta-analyses, selected from 75 articles.

Focusing on the self-esteem of gifted youth, we regret that the literature provides inconsistent or even contradictory results. On the whole, most studies conclude that gifted youth exhibit a rather high level of self-esteem, equivalent to that of their non-gifted peers (Bakar 2020; Bartell and Reynolds 1986; Brody and Benbow 1986; Chiu 1990; Colangelo et al. 1987; Dean 1977; Fanaj and Mustafa 2021; Field et al. 1998; Li and Shi 2019; McEwin and Cross 1982; Tidwell 1980; Vialle et al. 2005; Vialle et al. 2007; and Winne et al. 1982). Other studies showed that gifted youth may even have higher self-esteem than their non-gifted peers (Ball et al. 1994; Chan 1988; Chiu 1990; Cornell and Grossberg 1987; Ghobary and Hejazi 2007; Lehman and Erdwins 1981; Pearson and Beer 1990; Roznowski et al. 2000; Sarouphim 2011; and Van Tassel-Baska et al. 1994). Finally, a few studies found that gifted youth may have lower self-esteem than their non-gifted peers (Bénony et al. 2007; Casino-García et al. 2021; and Lea-Wood and Clunies-Ross 1995). Regarding these results, it seems worth highlighting two points: Firstly, one study (Chiu 1990) showed that teachers rated gifted youth as having higher self-esteem than their peers when the gifted youth themselves reported a level of self-esteem similar to that of their non-gifted peers. This is an important finding, as it echoes misconceptions held in public opinion, including among the people who surround gifted children and teenagers. It cannot be ruled out that this discrepancy in self-reported assessments by gifted youths and hetero-reported assessments by their teachers depicts these false beliefs, underscoring the interest of including this section in this literature review. Secondly, the authors of one of the four studies that found significantly lower self-esteem scores in gifted youth (Casino-García et al. 2021) warn that the results reported by gifted youth remain high, close to the maximum score of the scale (mean score in the gifted group = 4.00 on the Rosenberg global self-esteem scale, with scores ranging from 2.20 to 5, versus mean score in the non-gifted group = 4.20). Unfortunately, the two studies reporting significantly lower self-esteem scores among gifted youth (Bénony et al. 2007; Lea-Wood and Clunies-Ross 1995) do not describe the details of the scores (either at the level of the participants’ scores, or at the level of the scales), which does not allow us to go any further. However, this observation underlines that a lower self-esteem in gifted youth does not necessarily imply a low self-esteem per se.

A well-known characteristic about giftedness is that it brings together a wide variety of profiles of gifted youth, sharing very different experiences, if only in relation to the multiple modalities of gifted education. We mentioned at the start of this section that the development of self-esteem is partly based on youth’s history of experiences, particularly in the school environment. It is therefore necessary to consider the potential effect of different gifted education programs on the self-esteem of gifted students. Homogeneous grouping is described in the literature as having a deleterious effect on the academic self-concept of gifted youth (especially through the big-fish-little-pond effect described in the section on performance anxiety; for more information, see the meta-analysis by Fang et al. 2018). However, most quantitative studies failed to show an effect of homogeneous grouping on the self-esteem of gifted youth, either a negative effect (n.s in Chan 1988) or a positive effect (n.s in Preckel et al. 2016). The most recent meta-analysis to our knowledge addressing grouping’s influence on gifted youth’s self-esteem (Kulik and Kulik 1992) concludes from 13 studies that there is no significant effect of ability nor cross-class groupings on gifted youth’s self-esteem. Based on 11 of these 13 studies, however, it was noted that the impact of homogeneous grouping on self-esteem varied according to ability level. Teaching in homogeneous classes for the gifted would tend to reduce students’ self-esteem (mean effect of −0.15), in contrast to homogeneous classes for students with lower abilities (mean effect of 0.19). Decades after the publication of this meta-analysis, a study brought new results (Courtinat-Camps et al. 2012). This study involved *N* = 255 gifted junior high school students (including *n* = 204 participants in homogeneous grouping classes and *n* = 51 students in heterogeneous grouping classes, all identified as gifted according to an IQ ≥ 130). The results of this study are significant despite a small effect size (*p* < .01, η^2^ = 0.055), and suggest that gifted self-esteem would be higher in heterogeneous classes than in homogeneous groups. Similar findings were reported in a qualitative study (Adams-Byers et al. 2004). 

Finally, rare studies focus on the self-esteem of gifted youth belonging to minority subgroups. For example, twice-exceptional youth (gifted with ADHD in Foley-Nicpon et al. 2012), gifted underachievers (Reis and McCoach 2000), or gifted students with learning disabilities (Faouri 1998) would be lower than for gifted youth with no distinctive characteristics, but their self-esteem scores would remain about average.

Overall, the literature shows a weak positive relationship between intelligence and self-esteem, as well as between academic achievement and self-esteem. Gifted youth on average display similar, if not higher, levels of self-esteem than their non-gifted peers (see Supplementary Table S10). Specialized literature, however, points to the vulnerability of some sub-populations of gifted youth, underlining once again the heterogeneity of gifted youth profiles and experiences. High self-esteem is a protective factor against anxiety, depression, and social difficulties such as loneliness and rejection (Alexopoulou et al. 2019; Baumeister 1997; Cross et al. 2006; Greenberg et al. 1992; Kaiser and Berndt 1985; Lewis and Adank 1975; Liu et al. 1992; Orth and Robins 2022; Preckel et al. 2016; and Stricker and Preckel 2022). Self-esteem also plays an adaptive role in everyday life, enabling us to cope with challenges and failures (Abdulla Alabbasi et al. 2020; Baumeister 1997; del Mar Ferradás et al. 2020; Stricker and Preckel 2022; Tidwell 1980; and Wills 1994). In this respect, it is a key point in youth development, for gifted and non-gifted alike, and one that adults should be vigilant about, especially since pre-adolescence is reported to be a more difficult period for all youth regarding self-esteem (Courtinat-Camps et al. 2012; Filosa et al. 2022; Stricker and Preckel 2022; and Wigfield and Eccles 1994).

### 3.6. Humor

Research is primarily interested in the cognitive component of humor: there is a consensus that humor depends on the effectiveness of certain cognitive abilities, specifically those involved in the detection, treatment, and resolution of incongruity, which can be associated with humor comprehension and production abilities (Bergen 2009; Vrticka et al. 2013; and Ziv 1990). As such, humor is a characteristic often associated with intelligence and intellectual giftedness (Holt and Willard-Holt 1995; Shade 1991). However, humor also relies on an emotional component (Vrticka et al. 2013; Willinger et al. 2017), and is often cited as a non-cognitive specificity of gifted youth (Holt and Willard-Holt 1995), which motivated its inclusion in this review.

#### 3.6.1. Humor: General Intelligence

Total number of studies: *N* = 9 studies, including no meta-analyses, selected from 48 articles.

Humor was studied under the prism of its relationship with intelligence, suggesting that a high intellectual ability would lead to a better sense of humor. Focusing specifically on intelligence and humor assessments in children and adolescents, few data are available to our knowledge. A first study (Hauck and Thomas 1972) conducted among elementary school children (*N* = 80) reported that the level of sense of humor, accessed by peer nomination, is highly correlated (*r* = 0.91) with students’ IQ scores. Two studies were conducted with adolescents regarding intelligence scores and objective assessment of humor skills. One study (Masten 1986) of 90 adolescents aged 10–14 years showed a weak to moderate positive correlation between humor and intelligence (*r* between 0.38 and 0.55 depending on the humor task). These results are in contrast with those reported in Cunningham (1962), a study of 70 high school girls who described a negative correlation between intelligence and humor skills (*r* = −0.25). This significant discrepancy between the results from empirical studies of children and adolescents may be explained in part by wide variations in the measurements and designs used in the studies (Holt and Willard-Holt 1995). However, one recent study (Vrticka et al. 2013) based on fMRI analysis included a small sample of children and pre-adolescents (*n* = 22 participants aged 6 to 13). The results suggest that a high intelligence (as measured by IQ) could favor the treatment of incongruities because IQ is associated with brain activity during humor processing. More results are available in university students (Greengross and Miller 2011; Kellner and Benedek 2016) and in adults (Christensen et al. 2018; Willinger et al. 2017), with studies showing weak to moderate positive correlations between different humor abilities and components of the Cattell–Horn–Carroll intelligence model.

#### 3.6.2. Humor: Specific Case of Giftedness

Total number of studies: *N* = 6 studies, including no meta-analyses, selected from 7 articles.

Part of the research on humor and giftedness focused on the humor of gifted youth, as perceived by others. Ziv (1990) data revealed that intellectually gifted adolescents are most often perceived by their peers as having a good sense of humor, or conversely, as having little humor. Such a bimodal distribution appears to be unique to the intellectually gifted adolescent population: this pattern is different from non-gifted adolescents who follow a normal distribution of peer-perceived humor scores. Another study (Barnett and Fiscella 1985) reported no difference in performance between gifted preschoolers (as defined by IQ ≥ 130) and control participants, as perceived by teachers. However, this study did not directly address humor, which was assessed as a dimension of playfulness. Finally, asking intellectually gifted children and pre-adolescents (*n* = 74) directly about their own sense of humor, they rated themselves as having a high level of humor (Bergen 2009).

Other authors studied humor through differences in scores between participants on tasks measuring the ability to understand, produce, and appreciate humor. A first study (Shade 1991) reported higher scores for humor comprehension and appreciation in intellectually gifted children and adolescents (grades 4, 6, and 8) than in their non-gifted peers. Based on interviews with children and pre-adolescents and a comparison with normative data collected in earlier studies from control subjects, Bergen (2009) provided similar findings regarding the understanding of humor. The author also observed that intellectually gifted younger children were able to produce and explain more jokes overall than their non-gifted peers. This study found no qualitative differences in the preferences of gifted children for certain types of humor compared to their peers, with all youth favoring humor based on the observation of incongruity. More recently, a study including only female adolescents examined the scores of 60 gifted and 60 non-gifted participants (Sharifi and Sharifi 2014). The results reveal higher humor scores overall in the gifted group. However, the tool used to assess humor in this study is unclear, with the scale as described not matching the provided reference (Thorson and Powell 1993); the listed humor dimensions of “creativity, coping, facilitating and being grateful” were not detailed within the article. This makes it difficult to interpret the results.

Lastly, a small number of studies specifically investigated dark humor and irony in giftedness. One study (Shade 1991), including 60 children and pre-adolescents in each group (gifted and non-gifted), reported that gifted participants had higher mean scores than their peers for spontaneous mirth response (measured by a trained assistant’s assessment of the facial mirth response, based on a five-point Likert scale ranging from 0 = negative response (grimace, etc.) to 4 = laugh) and comprehension on verbal satire items. Another study of 23 intellectually gifted and 73 non-gifted adolescents focused specifically on the issue of irony (Bianchi et al. 2017), and found that intellectually gifted adolescents had better abilities to understand (*d* = 0.58) and produce verbal irony (*d* = 0.66).

In summary, the few studies that focused on humor among gifted youth seem to show that overall, gifted youth have better abilities than their non-gifted peers to understand, produce, and appreciate humor (see Supplementary Table S11), presumably due to the cognitive requirements of humor-related tasks. These findings also seem to apply to satire and irony. The results of self-reported measures are less clear: sometimes humor can also be challenging for intellectually gifted youth whose sense of humor may not be understood or appreciated by peers. Indeed, it is necessary to understand the different elements of a joke or pun in order to appreciate it, which requires that the person producing humor and the spectator are on the same page (Holt and Willard-Holt 1995). Another aspect to consider in order to better understand giftedness and its specificities could therefore be to investigate the knowledge and interests shared between gifted youth and their non-gifted peers, which is covered in the next section.

### 3.7. Interests

Total number of studies: *N* = 2 studies, including no meta-analyses, selected from 16 articles.

One possible source of differences between gifted youth and their peers is in the interests that they develop and value: in other words, the things that capture their attention and that they enjoy in their free time (Ziv 1990). This hypothesis is mainly based on clinical observations; for example, in Simoes Loureiro et al. (2010) or in the Cohen (1989) single case study.

Empirical studies in this field are almost non-existent (see Supplementary Table S12). Part of the evidence comes from asking teachers involved with gifted students. One study (Schack and Starko 1990) reported that teachers attending intellectually gifted youth cited multiple interests as one of the three criteria they used most to identify intellectual giftedness when selecting children to join special education programs, whereas teachers from regular classes mentioned achievement criteria instead. As for studies asking gifted youth directly, an ancient study by Lehman and Witty (1927) was carried out on 50 intellectually gifted children compared to non-gifted peers on their preferential activities and versatility of interests. The results did not differ between the two groups in terms of versatility and number of interests, but participants in the gifted group were more solitary in their play and more engaged in reading activities according to frequency and time devoted.

More recent outcomes about intellectually gifted young people’s interests in relation to those of their peers are provided by (Roznowski et al. 2000). Their study included 12,630 10th grade students followed for three years, and was based on a database used by a national longitudinal studies program. Participants were separated into ability groups based on a large battery of cognitive tests summarized into composite scores; the gifted group was defined as the top 5% of composite scores. According to their results, intellectually gifted adolescents spend more time doing homework, have more academic interests than their peers, and watch less TV. The authors conclude, however, that intellectually gifted adolescents, although more serious and hard-working, are far from the dynamic of withdrawal from social life as might be imagined: “activity participation, contrary to negative stereotypes of talented students, indicates that higher ability students participate at least as much as the typical student across the board and in several cases, participate more than the average”.

In sum, the issue of interests among intellectually gifted youth would deserve much more investigation to reach a definitive conclusion. Although there is no evidence, other than personal experience, that intellectually gifted youth differ from their non-gifted peers in terms of interests or hobbies due to intellectual giftedness, it remains a plausible and logical hypothesis to consider. Knowing more about the differences between gifted and non-gifted youth in terms of their interests would allow for a better understanding of the differences observed within other processes. This information would be particularly useful in adolescents because it may be highly related to other sensitive variables at this time of life, such as humor or socialization.

### 3.8. Moral Development

Moral development, which encompasses a plurality of concepts, includes a cognitive component of moral judgment and moral reasoning, as well as an emotional component referring to moral sensitivity and moral motivation (Beißert and Hasselhorn 2016; Derryberry et al. 2005).

#### 3.8.1. Moral Development: General Intelligence

Total number of studies: *N* = 11 studies, including no meta-analyses, selected from 39 articles.

In the case of the cognitive component, several studies highlighted the involvement of cognitive abilities in moral development (Derryberry and Barger 2008; Hoffman 1977; Lee and Olszewski-Kubilius 2006; Lee et al. 2020; Simmons and Zumpf 1986; and Tirri and Nokelainen 2007). Many complex cognitive processes, such as considering, selecting, processing, and interpreting multiple intrinsic and extrinsic factors, various contexts, and perspectives as a whole, but also anticipating the consequences of actions and applying social norms accordingly, are essential for moral development (Beißert and Hasselhorn 2016; Derryberry et al. 2005). Certain personality traits, such as openness to experience, often associated with high intelligence, would also be a characteristic conducive to accelerated moral development (Derryberry et al. 2005). These findings led to the assumption that high cognitive abilities should be systematically associated with high moral development.

Based on this hypothesis, a number of authors assumed that moral development should be associated with giftedness, but moral development was little studied in the context of general intelligence. One study of interest (Beißert and Hasselhorn 2016) recently found an absence of correlation between moral development and nonverbal intelligence, as assessed by figurative inductive reasoning tests, such as the Cattell Culture Fair Intelligence Test and matrices. To explain this counterintuitive finding, the authors suggested that the involvement of cognitive abilities usually assigned to moral development does not reflect a general relation between intelligence and moral development, but is due instead to the nature of the psychometric tools used to measure moral development.

Indeed, many moral development tests rely on verbal abilities. For example, the widely used Defining Issues Test (DIT) of moral judgment appears to be highly dependent on the individual’s verbal abilities (Karnes and Brown 1980; Sanders et al. 1995), although it is a different construct (Derryberry and Barger 2008). Moreover, knowledge is thought to play a significant role in the assessment of moral development (Barone and Barone 2018). Reflecting this point, an individual’s levels of formal education and social maturity would explain between 30% and 50% of the variance observed in Defining Issues Test scores (Alnabhan 2011; Derryberry et al. 2005). Such confounding variables could be a source of significant bias in creating a correlation with general intelligence, and possibly with intellectual giftedness.

#### 3.8.2. Moral Development: Specific Case of Giftedness

Total number of studies: *N* = 21 studies, including no meta-analyses, selected from 27 articles.

Some findings suggest that intellectually gifted youth could be more likely to experience greater or faster moral development than their peers. This issue was particularly addressed in studies with gifted adolescents and interested in the cognitive component of moral development, i.e., moral reasoning and moral judgment abilities. With respect to moral reasoning (rationally thinking about moral concerns) empirical research repeatedly found higher performance in intellectually gifted adolescents relative to their peers (Chovan and Freeman 1993; Howard-Hamilton and Franks 1995; Karnes and Brown 1980; and Tan-Willman and Gutteridge 1981). With respect to moral judgment, the other component of moral development, more recent studies obtained similar results with higher abilities for intellectually gifted adolescents compared to non-gifted peers, and sometimes compared to older college students (Alnabhan 2011; η^2^ = 0.29, *d* = 1.07 in Derryberry and Barger 2008; η^2^ = 0.05, *p* = .005 in Derryberry et al. 2005; *d* = −0.11 to 0.50 in Lee and Olszewski-Kubilius 2006; Tirri and Nokelainen 2007; and Tirri and Pehkonen 2002). In other words, gifted adolescents seem better able to evaluate whether a situation is moral or not than their peers. 

Moral judgment (the other aspect of the cognitive component of moral development) can be divided into three levels: a first level that focuses on the satisfaction of personal needs and interests, a second level oriented around norms and sanctions by authority, and the last level involving moral principles of justice and fairness (Derryberry and Barger 2008). Little empirical research was conducted directly with intellectually gifted children, but the results appear generally similar to those observed in intellectually gifted adolescents; namely that gifted children exhibit more advanced moral development than their non-gifted peers at the same age (Kohlberg 1964 and Gross 1993 cited in Lee and Olszewski-Kubilius 2006; Simmons and Zumpf 1986). Some qualitative studies and case studies also agree with these findings (Ambrose and Cross 2009; Hollingworth 1942; Lovecky 1992; and Roeper and Silverman 2009).

With regard to these results, high intellectual abilities seem to allow for a high level of moral development in gifted children and adolescents who experience better moral judgment scores than their non-gifted peers, on average (see Supplementary Table S13). On the other hand, intellectual giftedness and high abilities are not always predictive of higher moral development (Derryberry and Barger 2008; Narváez 1993; and Tirri and Nokelainen 2007). Moreover, being capable of advanced moral reasoning and/or judgment does not necessarily lead to better decision making and moral behavior (Derryberry and Barger 2008; Tirri 2010). This is particularly important when considering the fact that dilemmas used to measure moral development are very different from dilemmas encountered by young people in everyday life, whether intellectually gifted or not (Beißert and Hasselhorn 2016; Lee et al. 2020; and Tirri and Nokelainen 2007).

In summary, giftedness seems to be associated with better moral reasoning and moral judgment abilities in complex situations, which does not necessarily imply that gifted individuals would have a greater sense of justice or would act in a more moral way in everyday life. In fact, it is mainly cognitive specificities that would be involved in differentiating the sense of morality and justice of gifted youth from their peers, and not non-cognitive specificities such as personality or insight. It is also worth considering that these differences in abilities between gifted and non-gifted youth may partly summarize a task effect, which would be interesting to investigate further in future studies to support the current findings.

### 3.9. Leadership

Leadership, as defined by the American Psychological Association (2021), refers to “the processes involved in leading others, including organizing, directing, coordinating, and motivating their efforts toward achieving certain group or organizational goals”. Leadership is associated with intelligence and claimed as a purported characteristic of intellectually gifted individuals (Matthews 2004; Peairs et al. 2019), although there are very few studies on the subject.

#### 3.9.1. Leadership: General Intelligence

Total number of studies: *N* = 5 studies, including 4 meta-analyses, selected from 150 articles.

Several meta-analytic works and literature reviews addressed the question of a possible correlation between intelligence and some constituents of leadership capacity (effective leadership in Hoffman et al. 2011; objective and perceptual measures of leadership in Judge et al. 2004; leadership perceptions and leader emergence in Lord et al. 1986). These works highlight the existence of a low to moderate positive correlation between intelligence and leadership, in the *r* = 0.20 range, relatively stable from one study to another. One study posterior to these meta-analyses (Daly et al. 2015) also showed that childhood cognitive ability would predict a significantly higher probability of leadership role occupancy at different times in adulthood. Along the same lines, the meta-analysis of Mills (2009) reported on the correlation between emotional intelligence and effective leadership and noted a moderate to strong positive correlation relationship across studies (*r* = 0.38 on average). However, a wide heterogeneity of the correlation coefficients examined in this meta-analysis should be noted, with correlations ranging from −0.03 to 0.90.

#### 3.9.2. Leadership: Specific Case of Giftedness

Total number of studies: *N* = 8 studies, including no meta-analyses, selected from 53 articles.

Considering specifically the leadership abilities of intellectually gifted youth, a first major difficulty is the relative lack of existing studies devoted to this topic (see Supplementary Table S14). Moreover, research in this field of study is inconsistent, due in part to a wide variability in theories and definitions of intellectual giftedness and leadership throughout various studies (Lee et al. 2020; Matthews 2004). However, a few studies do provide useful insights into the relation between intellectual giftedness and adolescent leadership abilities. Lee and Olszewski-Kubilius (2006) found higher leadership scores in intellectually gifted participants, on average, compared to normative data (*d* = 0.67). The intellectually gifted group included 234 high school students from enrichment programs at an American summer camp. In this study, the identification of intellectual giftedness was in fact based on the adolescents’ academic performance (SAT), which is not consistent with recommendations for intellectual giftedness. Paradoxically, small but significant negative correlations were observed between SAT math and SAT combined (SAT math and verbal) scores and leadership scores, suggesting that higher levels of academic ability were associated with lower levels of leadership; this is relatively inconsistent with the idea that gifted participants have better leadership abilities. To our knowledge, this is the only study reporting direct measures of leadership abilities in intellectually gifted adolescents; and no similar study is available in children. However, a study carried out among 34 intellectually gifted elementary school students (grades 4 through 6) suggests that gifted children would perceive themselves as leaders as much as their non-gifted peers on average (based on normative data; Riley and Karnes 1994).

Some hypotheses were proposed to understand the possible relationship between giftedness and leadership. First, research on this subject mostly addresses the development of leadership abilities in intellectually gifted adolescents as “a predisposition”, “an aspiration to”, or “a potential” (Bronk et al. 2010; Lee and Olszewski-Kubilius 2006; Lee et al. 2020; and Peairs et al. 2019), suggesting leadership is a talent inherently present in each intellectually gifted individual from birth to be developed over a lifetime. In this view, intellectually gifted adolescents would be likely to develop their leadership skills more quickly, to take up more challenges, and to assert themselves more easily as leaders within a group, due to their high cognitive abilities (Antonakis et al. 2019; Pérez et al. 2020). For example, Muammar (2013) found that intellectually gifted students had better planning skills than their non-gifted peers, which should benefit developing leadership skills. For Antonakis et al. (2019), achievement is a mediating variable linking intellectual abilities to leadership abilities. Since intellectually gifted students generally have high levels of achievement relative to their non-gifted peers (cf. objective indicators of achievement and quality of life), the authors suggest that they could develop leadership abilities more easily as a consequence. Furthermore, reflecting this common belief that high abilities lead to high leadership potential, some authors (Peairs et al. 2019) report in their research that intellectually gifted adolescents are more likely than nongifted adolescents to be identified as leaders by their peers.

Another way of looking at this issue is to investigate the dispositional and motivational aspects of leadership in giftedness. Empirical studies put forth that gifted individuals could demonstrate advanced social responsibility, and high levels of civic awareness and engagement, which would foster the development of leadership skills (Lee et al. 2020). In the latter study, including over 400 academically high-achieving high school students, the authors concluded through their own investigation that a higher proportion of participants within the gifted group expressed motivation to become a leader in society. Recently, the focus also shifted from quantitative differences to qualitative differences in the leadership styles of intellectually gifted and non-gifted adolescents. This issue was addressed by Lee et al. (2020); significant differences are found between the groups in terms of leadership style preferences. Intellectually gifted adolescents reported a preference for task and performance-based leadership (*r* = 0.15), which depends on a leader’s ability to make decisions for the group (*r* = 0.24). On the other hand, non-gifted peers reported a preference for people-focused leadership, relying on the ability of followers to make decisions within the limits imposed by the leader. Another recent study (Peairs et al. 2019) involving 202 gifted adolescents, addressed the issue of qualitative differences in leadership between gifted and non-gifted youth, but this time with an experimental design based on teachers’, peers’, and the gifted students’ own views. A first result was that teachers perceived intellectually gifted adolescents as more directive leaders than their non-gifted peers in general. With regard to leaders in particular, the results show that peers viewed gifted leaders as more pro-social in their leadership behaviors than non-gifted peers, and gifted leaders described themselves as more influential and more intimidating leaders than others.

Although there seem to be differences in the leadership style preferences and perceptions of gifted and non-gifted youth, the limited literature on this field is not sufficient to claim that leadership is a defining characteristic of intellectually gifted individuals. Apart from qualitative differences, there is no sufficient evidence to conclude that all gifted youth have greater leadership potential or abilities than non-gifted youth, although it may be the case for some.

### 3.10. Emotional Intelligence

There are two major approaches to studying emotional intelligence (Zeidner et al. 2005), depending on whether emotional intelligence is considered a personality trait (as in the work of Goleman 1995) or an aptitude, as traditional intelligence (as in the works of Salovey and Mayer 1990, or Mayer et al. 2000).

Emotional intelligence viewed as a trait is mostly assessed by subjective self-report questionnaires. This approach focuses on the perceptions of individuals in emotional contexts, meaning on what they feel and how they react, rather than on what they should do according to known norms. In this view, intellectually gifted children and adolescents can be considered as having an atypical emotional development, an atypical emotional profile, or specific needs on the emotional level, which would benefit from the implementation of support programs to prevent possible socio-emotional difficulties or under-achievement (Brasseur and Grégoire 2010; Casino-García et al. 2021; Schwean et al. 2006; Zeidner 2017; and Zeidner and Matthews 2017).

By contrast, emotional intelligence viewed as an ability is closer to the canonical view of general intelligence in the sense that it is structured as a hierarchical set of cognitive abilities ranging from the ability to perceive and express our emotions to the ability to manage the emotions of others and regulate our own emotions (Li et al. 2017). Emotional intelligence is assessed through performance measures. The subdimensions of emotional intelligence vary across measurement tools, but some subscales are common (for example, stress and emotion management, social skills, and adaptability subscales are present in both the TEIQue and the Bar-On EQi-Young version). In this view, intellectually gifted children and adolescents may have superior abilities to process emotional information, in line with their generally high cognitive efficiency, which would improve their understanding of their own and others’ emotions (Chan 2003; Guignard and Zenasni 2004; and Matthews et al. 2018). In this case, emotional ability development programs could be beneficial to intellectually gifted children and adolescents in ways similar to acceleration or leadership programs.

There are few empirical studies specifically interested in the emotional intelligence of intellectually gifted children and adolescents (Brasseur and Grégoire 2010; Guignard and Zenasni 2004), and these different views of emotional intelligence led to wide variability across methods and results, which makes these studies particularly difficult to compare.

#### 3.10.1. Emotional Intelligence as a Trait: General Intelligence

Total number of studies: *N* = 7 studies, including 4 meta-analyses, selected from 203 articles.

Shared results with emotional intelligence as an ability: general intelligence.

Some authors investigated the relationship between academic performance and an emotional intelligence trait. Several meta-analyses report a modest to moderate overall correlation (*r* = 0.10 self-rated measures and *r* = 0.13 for mixed measures, including assessments of emotion-related abilities and traits, in MacCann et al. 2020; *r* = 0.20 in Perera and DiGiacomo 2013; *r* = 0.26 for self-rated measures; and *r* = 0.24 for mixed measures in Sánchez-Álvarez et al. 2020). Similar findings on the relationship between emotional intelligence traits and academic performance are still emerging (from *r* = 0.05 to *r* = 0.19 according to school domain in Li and Shi 2019). Studies concerning the relation between general intelligence and emotional intelligence are more rare. A recent study (Li et al. 2017) assessed general intelligence using Cattell’s Culture Fair Test and the emotional intelligence trait using the TEIQue in children and found a correlation of *r* = 0.28 in a control sample. It is important to note, however, that this weak positive correlation between the general intelligence and emotional intelligence trait was not found in a sample of gifted children despite the use of similar psychometric measures (Raven’s Standard Progressive Matrices as a measure of nonverbal reasoning ability instead of Cattell’s Culture Fair Test in Li and Shi 2019; *r* = −0.18). Overall, it seems that, on average, youth with high academic success and high cognitive abilities demonstrate higher emotional intelligence scores up to a point.

#### 3.10.2. Emotional Intelligence as a Trait: Specific Case of Giftedness

Total number of studies: *N* = 10 studies, including 2 meta-analyses, selected from 29 articles.

Shared results with emotional intelligence as an ability: specific case of giftedness.

A number of studies tested trait emotional intelligence in gifted youth, with some identifying giftedness based on IQ scores (IQ ≥ 125 in Brasseur and Grégoire 2010; IQ ≥ 130 in Schwean et al. 2006) and comparing the results with a group of non-gifted peers, and others identifying giftedness based on academic achievement and comparing gifted scores to normative data (Corso 2001; Lee and Olszewski-Kubilius 2006). The majority of studies found no general difference between gifted participants and their peers (Brasseur and Grégoire 2010; Lee and Olszewski-Kubilius 2006; and Schwean et al. 2006), with the exception of one study reporting higher trait emotional intelligence among gifted participants identified based on achievement (Corso 2001). The results can be heterogenous regarding subscales: for the adaptability and stress management subscale in particular, studies tended to find better scores for gifted participants, with the exception of the Lee and Olszewski-Kubilius (2006) study, in which intellectually gifted adolescents scored below the norm on stress management.

Some differences can be observed between the studies with respect to the subscales, in part because they use different tools to measure the emotional intelligence trait (TEIQue in Brasseur and Grégoire 2010; EQi in Corso 2001; Lee and Olszewski-Kubilius 2006; and Schwean et al. 2006). Nevertheless, when differences are observed across subscales, they are almost always in favor of the gifted participants. One study reports higher scores for gifted youth on different empathy subscales (Brasseur and Grégoire 2010), and another one finds that intellectually gifted participants scored higher on the interpersonal dimension (Schwean et al. 2006). Only the study by Lee and Olszewski-Kubilius (2006) indicates a lower score in the gifted group compared to the normative data for the impulse control subscale.

In summary, existing studies show no differences between gifted and non-gifted youth on measures of the emotional intelligence trait in general (see Supplementary Table S15). Differences can be observed at a subscale level, but these differences are not consistent across studies and do not show a general trend, except that gifted youths are about as emotionally adjusted as their peers. Based on these findings, no specific emotional style or profile of intellectually gifted children and adolescents emerges in terms of emotional intelligence traits (Brasseur and Grégoire 2010; Matthews et al. 2018).

#### 3.10.3. Emotional Intelligence as an Ability: General Intelligence

Total number of studies: *N* = 7 studies, including 4 meta-analyses, selected from 203 articles.

Shared results with emotional intelligence as a trait: general intelligence.

Two recent meta-analyses report moderate relations between emotional intelligence ability and academic performance (*r* = 0.24 in MacCann et al. 2020; *r* = 0.31 in Sánchez-Álvarez et al. 2020). A more recent study shows a weak relationship between these two variables (*r* = 0.19 for achievement in language and *r* = 0.04 for achievement in mathematics in Perpiñà Martí et al. 2023). Focusing specifically on intellectual abilities as measured by intelligence tests, studies reached the same conclusion of a moderate relation: the meta-analysis of Kong (2014) found correlations between trait emotional intelligence and general intelligence (*r* = 0.33), verbal intelligence (*r* = 0.26), and nonverbal intelligence (*r* = 0.27) scores. Overall, the relationship of intellectual abilities with the ability for emotional intelligence seems to be stronger than with trait emotional intelligence.

#### 3.10.4. Emotional Intelligence as an Ability: Specific Case of Giftedness

Total number of studies: *N* = 10 studies, including 2 meta-analyses, selected from 29 articles.

Shared results with emotional intelligence as an ability: specific case of giftedness.

The ability approach of emotional intelligence has more theoretical support according to some authors (Zeidner 2017), but it is generally less studied in the context of intellectual giftedness. One well-known study on the subject is that of Mayer et al. (2001) and collaborators exploring the characteristics of “emotional giftedness” defined as high emotional abilities in 11 adolescents. The study is built on a multiple case study design that required each participant to describe a social situation in which a friend wanted them to do something they were uncomfortable with, as well as to answer questions about their own emotions and those of their parents. Emotional intelligence was assessed by ability scores. The authors found that, in difficult social contexts, participants with higher emotional intelligence were better able to identify their own and others’ emotions, including complex emotions, and used them wisely in developing an appropriate behavioral response. This study is not informative regarding the relation between “emotional giftedness” and intellectual giftedness, however. Another study (Woitaszewski and Aalsma 2004) found no difference between 39 intellectually gifted participants and normative data, with no correlation between general intelligence and emotional intelligence measured as an ability. More recently, a study of young women only (Sharifi and Sharifi 2014) reported a significant difference between the emotional intelligence scores of 60 intellectually gifted and 60 non-gifted adolescents, in favor of the gifted group.

Of particular interest, one study (Zeidner et al. 2005) attempted to bridge the gap between the two approaches of emotional intelligence by comparing the scores of intellectually gifted (*n* = 83) and non-gifted (*n* = 125) adolescents on measures of emotional intelligence treated both as a trait and as an ability, also controlling for participants’ vocabulary. The results show that adolescents in the intellectually gifted group scored significantly higher on tests measuring ability for emotional intelligence (*d* = 0.39), but significantly lower than their non-gifted peers on trait emotional intelligence (*d* = −0.57). Overall, intellectually gifted youth had higher scores on aptitude tests than on self-assessment tasks, which may be explained in part by the fact that total emotional intelligence ability scores are correlated with participants’ vocabulary level (closely related to crystallized intelligence). There was also little correlation between trait emotional intelligence and ability tests, which supports the idea that comparing studies requires careful consideration of the definitions and the measures they use for emotional intelligence.

These results are supported by two recent meta-analyses, mainly including studies conducted in intellectually gifted children and adolescents (*g* = 0.23 in Abdulla Alabbasi et al. 2020 with 21 out of 25 studies involving gifted participants and control groups in the children/adolescent age range, and *g* = 0.12 in Ogurlu 2020 with 11 out of 16 studies involving gifted participants and control groups in the same age range). Both studies also found a moderating effect of the measurement tool and theory of emotional intelligence being used, with higher scores for gifted youth and a larger difference with controls in studies treating emotional intelligence as an ability (see Supplementary Table S16).

In sum, findings regarding emotional intelligence in gifted youth are mixed, but sufficient to suggest that intellectually gifted youth do not display specific emotional difficulties (Matthews et al. 2018). The plurality of emotional intelligence concepts and measurement tools make studies on emotional intelligence a still controversial concept (for critical reviews, see Schulte et al. 2004; Waterhouse 2006; Zeidner et al. 2005; and Zeidner and Matthews 2017) in need of further research for definitive conclusions.

### 3.11. Overexcitabilities

Total number of studies: *N* = 17 studies, including 1 meta-analyses, selected from 31 articles.

The concept of overexcitability is that each individual could experience extreme psychological intensity and sensitivity in behavior that would be expressed through one or more of the following five forms of overexcitabilities (Mendaglio 2008, 2012; Piechowski and Colangelo 1984): psychomotor (organic excess of energy), sensual (increased experience of sensory pleasures), intellectual (intensified activity of the mind, to be differentiated from the cognitive domain of intelligence), imaginative (rich inventiveness, to be differentiated from the cognitive domain of creativity), and emotional (intense emotional experiences). The construct of overexcitability is a component of Dabrowski’s theory of positive disintegration, in which overexcitabilities would play a role in the moral and emotional development of individuals. However, the theory of positive disintegration is not a giftedness theory nor an overexcitability-centered theory (Ackerman and Heggestad 1997; Harper et al. 2017; Mendaglio 2008, 2012; and Vuyk et al. 2016). Yet, it is often considered in both clinical and research settings that overexcitabilities are particularly relevant to intellectually gifted youth, consistent with the idea of an asynchronous development (see Bailey 2011; Guthrie 2019; Matthews et al. 2018; and Ogurlu 2020). In this section, we differentiate overexcitabilities from sensory processing and sensory discrimination (e.g., see Gere et al. 2009), two domains for which there are no clear results in gifted youth.

There are still few empirical studies on overexcitabilities in intellectually gifted children and adolescents: this field of research was guided primarily by clinical observations (Guignard and Zenasni 2004). Nevertheless, a recent meta-analysis (Winkler and Voight 2016) offers a valuable synthesis. All the studies included in the meta-analysis had a control group, and 10 out of a total of 12 studies included concern intellectually gifted children (Bouchard 2004; Breard 1995; Siu 2010; and Tieso 2007) and intellectually gifted adolescents (Ackerman and Heggestad 1997; Harrison and Haneghan 2011; Limont et al. 2014; Piirto and Fraas 2012; Wirthwein et al. 2011; and Yakmaci-Guzel and Akarsu 2006), defined based on high intellectual performance. The multiple results considered in this meta-analysis indicate that the overexcitability levels of intellectually gifted children and adolescents are overall significantly higher than those of their non-gifted peers. The scores on the overexcitability subscales show that intellectual overexcitability appears to be the most discriminating domain between the groups of intellectually gifted and non-gifted participants across studies, with a weighted mean effect size described as medium by the authors (effect size of 0.55 based on the unbiased standardized mean differences). Differences observed between gifted and non-gifted groups in imaginative overexcitability scores are small to moderate (weighted mean effect size of 0.36), and differences in emotional and sensual overexcitabilities are small (weighted mean effects sizes of 0.19 and 0.22, respectively). Differences in psychomotor overexcitability are not statistically significant on average (for a summary, see Supplementary Table S17).

The literature claims (Bouchet and Falk 2001; Gross et al. 2007; and Tieso 2007) that females that are intellectually gifted score higher than their male peers on sensual and emotional overexcitabilities, while intellectually gifted boys may exhibit higher intellectual and psychomotor overexcitabilities. However, results from Tieso (2007) indicate that this gap between female and male participants on emotional and sensual overexcitabilities is similar or stronger among non-gifted participants than among gifted ones, so this is not a specificity of the gifted youth. In addition, this pattern may reflect gender stereotyping, which requires particular caution in interpreting the reported results (Bouchet and Falk 2001).

Overexcitabilities were studied mainly with the idea of finding another criterion to identify intellectual giftedness. Yet, the available evidence is not supportive. For example, one study (Bouchard 2004) reports that similar overexcitabilities profiles were obtained by 76% of children in the intellectually gifted group and 42% of children in the control group. Other authors (Rinn et al. 2010) found in their study involving 379 intellectually gifted adolescents from a summer university program for gifted children (with a criterion for inclusion of an IQ score ≥ 125) that there were four different overexcitabilities profiles among these young people. Overexcitabilities are therefore not a homogeneous identification criterion. Finally, this work was supplemented in a study using the same measurement instrument (Alias et al. 2013) that noticed that there is a profile of intellectually gifted adolescents who do not experience overexcitability at all (22% of the gifted participants’ sample).

In sum, overexcitabilities are not a discriminating criterion in identifying giftedness. In addition to the wide heterogeneity of overexcitabilities profiles reported for gifted youth, it appears that similar high levels of intelligence can be associated with distinct overexcitability profiles or no overexcitability at all. There are also many methodological issues to be raised with regard to this field of study. First, overexcitabilities are distorted from their original purpose in being used as descriptive personality traits (for a critical review, see Vuyk et al. 2016). Overexcitabilities are also too close to other concepts (e.g., the big five model in Wirthwein et al. 2011); their conceptual proximity with cognitive activity (as in the case of intellectual overexcitability) can contribute to observed correlations with giftedness. Another problem is that the measurement tools available (OEQ-I, OEQ-II, ElemenOE) suffer from poor theoretical validity (Warne 2011). In the case of other report measures filled out by parents or teachers (such as the ElemenOE), there is a significant risk of a halo effect, which is a major concern in popular domains such as overexcitabilities and giftedness (Winkler and Voight 2016). Finally, beyond all these issues, overexcitabilities can lead to poor care strategies. Indeed, some symptoms associated with disorders such as ADHD, anxiety, or depression may be recognized as manifestations of overexcitability when they require disorder-specific care.

## 4. Discussion: Critical Review and Recommendations

### 4.1. Summary of the Findings

Research in psychology and education centered on intellectual giftedness greatly expanded in recent years, with a particular interest in related non-cognitive characteristics. A detailed summary of current findings (methodologies, effect sizes, and their interpretation) is presented in Supplementary Tables S1–S17.

Briefly, intellectually gifted children and adolescents are not significantly above their peers in terms of pathological and non-pathological anxiety as measured by overall scores, worry, intolerance of uncertainty, test anxiety, unhealthy perfectionism, or mood disorders such as depression and suicidal ideation. Increased vulnerability to bipolar and schizophrenic disorders was reported on occasion but the results need to be replicated due to insufficient data. High cognitive abilities appear to be a protective and resource factor regarding mental and physical health, objective life quality, and subjective well-being. With regard to the social sphere, results lead to the conclusion that intellectual giftedness is not an obstacle to development of successful social abilities, as assessed in terms of skills and as perceived by gifted youth themselves and peers. Mixed results are noted when parents and teachers are asked about the socialization of gifted youth, but such heterogeneity can be linked to the stigma of giftedness, briefly mentioned above. Specific difficulties may appear for highly gifted youth, but given the lack of quantitative research on the subject, no conclusions can be drawn at this time. Moreover, gifted youth do not seem to differ from their non-gifted peers when it comes to self-esteem.

Humor as a research topic does not yet lead to clear conclusions; data are almost only available for adolescents due to developmental considerations. Some studies conclude to similar or possibly higher humor abilities in intellectually gifted than non-gifted youth, especially regarding irony. This is possibly related to the fact that humor also requires effective cognitive processing. High cognitive abilities, as experienced in intellectual giftedness, may also support faster moral development and better leadership skills, but this does not seem systematic. Furthermore, although intellectually gifted youth may demonstrate a high level of moral development in complex situations, this does not necessarily mean that they are more likely to engage in everyday life. Finally, research on emotional intelligence and overexcitabilities does not support the idea that intellectually gifted children and adolescents would have a specific emotional profile or even particular emotional needs in daily life.

In sum, based on works carried out to date on children and adolescents, none of the characteristics addressed in this literature review can be considered as a diagnostic factor for intellectual giftedness. Research conducted in this field has the advantage of focusing attention on non-cognitive characteristics that may be problematic for intellectually gifted individuals in a clinical setting. By promoting a broad perspective on the specificities of an individual, these characteristics enhance our understanding of issues that may be raised by gifted and non-gifted children and adolescents.

### 4.2. Methodological Issues

#### 4.2.1. Inclusion Criteria

A major limitation of existing literature on giftedness is the wide heterogeneity in definitions and inclusion criteria for giftedness, which makes it particularly difficult to compare studies. In this literature review, we focused only on studies based on a definition of giftedness as high intellectual ability. We made this choice because we believe it is critical for inclusion criteria in gifted groups to be distinguished depending on whether one is talking about intellectual giftedness, creative giftedness, talent, or leadership, since what applies to intellectual giftedness does not necessarily apply to other types of giftedness.

Even when focusing on intellectual giftedness, the articles selected for this literature review used different inclusion criteria for gifted groups, as can be seen in the summary tables. Some issues are important to discuss. First, most studies reviewed here relied solely or partly on achievement test scores and/or academic achievement (Carman 2013), presuming that high academic scores necessarily reflect intellectual giftedness. Although intelligence level does correlate with academic achievement, it is misleading to systematically associate potential with achievement. This approach fails to detect intellectually gifted children and adolescents who are under-achieving, either as a coping strategy (Winner 2000) or as a consequence of disengagement from school. Conversely, the socio-cultural context of a child (e.g., income of parents) strongly impacts their academic results, which is a major limitation of the use of achievement-based criteria regarding intellectual giftedness (Hanushek et al. 2019). Such an inclusion criterion leads to a truncated representation of the gifted samples by focusing on a part of the population that may have particular profiles with regard to numerous variables, such as, for example, the relationship between leadership and achievement. 

Another inclusion criterion sometimes applied to gifted groups is teacher nomination. This method considers that teachers are able to identify intellectually gifted students within their classes based on a range of academic as well as psychological and socio-emotional criteria. However, the results from the present literature review show that non-cognitive characteristics commonly associated with intellectual giftedness cannot be used as diagnostic elements; and even if they were, it is doubtful whether teachers would be very reliable judges. A risk is that gifted samples based on teacher nominations may reflect stereotypes rather than objective intellectual giftedness (Deku 2013; Siegle and Powell 2004). In essence, this method suffers from the same limitation of being a result of academic achievement, although in a subtler way than with the direct use of school grades.

The use of psychometric tests to assess intellectual abilities is one of the most widely used criteria for inclusion in gifted groups and, in our opinion, the most relevant. However, the threshold for recognizing intellectual giftedness differs from one study to another and from one specialized gifted program to another. An IQ threshold of 130 and above is theoretically accepted (Carman 2013; to go further, McBee and Makel 2019) and is in line with current criteria used to identify intellectual disability (two standard deviations above or below the mean). Yet unfortunately, the criterion of IQ ≥ 130 is rarely applied within studies on intellectual giftedness and associated non-cognitive characteristics. 

A related criterion used in some studies (Alnabhan (2011) and Tirri and Pehkonen (2002) for moral development; Li et al. (2017) for emotional intelligence; Guez et al. (2018) for success in academic examination) is to refer to a test assessing fluid intelligence, i.e., abstract reasoning abilities, as a criterion for inclusion within gifted groups. This idea is motivated by the fact that fluid intelligence and general intelligence (*g*) are very strongly correlated (Kovacs and Conway 2016). This approach is valuable because it provides a measure of intellectual abilities that is less biased by culture and verbal abilities highly dependent on socio-cultural and economic levels (Neff 1938). Nonverbal intelligence tests provide particularly useful information in fields of study where there is high verbal input, such as the study of moral development and emotional intelligence. In addition, non-cognitive abilities are sometimes assessed using tasks that are closely related to verbal comprehension and expression skills. To ensure the validity of studies of giftedness using these types of measures, the verbal level of participants must be carefully controlled to improve confidence in the conclusions that are drawn. Observed differences may then be related to the variable being measured, such as high intellectual ability or high moral skills, rather than to differences in verbal ability.

Finally, some of the guidelines for identifying gifted students suggest that performance and non-performance identification methods can be treated as interchangeable. However, these gifted identification methods with and without performance tend to identify different students. For this reason, methods of gifted identification using ability tests or not cannot replace each other and should be used simultaneously (Acar et al. 2016).

#### 4.2.2. Sampling Methods and Sample Sizes

In a research field such as that of non-cognitive characteristics related to intellectual giftedness, including psychological and socio-emotional dimensions, it seems particularly relevant to be careful regarding sampling methods and their consequences: socio-emotional features of a child may be determinant in whether they are academically successful and whether they are identified as gifted or not, in turn creating the possibility of a severe sampling bias.

Unfortunately, when conducting this literature review, it became apparent that the vast majority of participants included in studies of gifted samples were from gifted academic programs or summer camps for the gifted. This severely limits the extent to which findings can be generalized to the general population of gifted children and adolescents. Only certain profiles of gifted youth may be selected to participate in such programs—presumably those who are academically successful and socially well-adjusted. It could also be the case that these particular settings influence gifted children’s and adolescents’ development by changing their experience of being gifted and their self- and peer representations.

Ideally, in order to capture the heterogeneous profiles of intellectually gifted youth, samples should be proportionally composed of participants actually representative of the intellectually gifted child and adolescent population. In particular, this would require broad inclusion of intellectually gifted participants not identified as intellectually gifted, and inclusion of highly gifted participants. Strictly adhering to these expectations would be overly ambitious, making it very difficult to conduct research on intellectual giftedness. A more realistic perspective in our opinion would be to favor feasible sampling methods that depend on the objective of the study (to report a general phenomenon common to all intellectually gifted youth, or a context-dependent phenomenon?). For studies of non-cognitive characteristics associated with intellectual giftedness, a good solution regarding expectations and feasibility would be to use a mixed sample design. For example, it would be optimal to replicate the same results with participants from specialized programs for gifted students (specialized classes for gifted students, summer university, etc.), participants from regular classes not yet identified as gifted (and tested through screening), and/or participants from associative and/or clinical settings.

It is fair to note, however, that access to some sampling methods strongly depends on which country the study is conducted in. For example, in the United States or Israel, specialized programs for the education of the gifted are widespread, but this is uncommon in other areas, such as West Europe. Systematic testing of children and systematic inclusion of gifted youth in specialized programs obviously facilitates the constitution of larger samples of gifted students (for example, see Lee and Olszewski-Kubilius 2006; Scholwinski and Reynolds 1985; Shechtman and Silektor 2012; and Zeidner and Schleyer 1999). Indeed, a recurring concern in research on giftedness is the difficulty in constituting large samples of gifted participants. In this literature review, we find a median gifted sample size of 97 participants (min = 15; max = 1062). Based on studies included in this paper, it would be reasonable to recommend sample sizes of at least 100 participants for future studies as a way to encourage greater robustness and representativeness of the results.

Another critical challenge for future studies in the field of non-cognitive characteristics in giftedness that stems from sampling considerations would be to fully consider the implications of cross-cultural comparisons (Hanushek 2021). Perceptions of giftedness can be vastly different from one place to another. In France for example, giftedness tends to carry a strong stigma and gifted children are often treated as nerds, i.e., as outsiders from the main group. The reverse can be true in other countries, potentially leading to very different socio-emotional consequences (e.g., a disadvantage versus an advantage in terms of socialization), and explaining inconsistent results in the literature.

Some studies examined the impact of cultural differences (including participants’ family, educational, and social backgrounds) on the overall experience of giftedness (Levy and Plucker 2003; Rodgers 2008; Thomas 2008; and Yoon and Gentry 2009), and some studies focused on giftedness representations in various countries (Chan 2002; Coleman and Cross 2014; Lee-Hammond 1999; Neumeister et al. 2007; and Tavani et al. 2009). It would be helpful to complement these findings with cross-cultural studies focusing on the different elements addressed in this literature review (see e.g., Lee et al. 2020), which would provide more robust conclusions than the comparison of results from very different cultures as we conducted here.

#### 4.2.3. Effect Sizes

Effect sizes are needed to correctly interpret statistical results (Pek and Flora 2018), and are a requirement of APA norms (7th edition). This indicator is very often missing within this field of literature. In the present study, 39% of included studies did not provide effect sizes. It is important to recall here that an effect can be statistically significant while being of very small magnitude. This is critical to correctly interpreting differences between gifted and non-gifted youth: a significant difference between gifted children and their peers does not have the same implications for clinical practice depending on whether their scores are on average 1% or 50% higher than their peers. Observing large effects should not be an absolute prerequisite for publication (small effects can also hint at meaningful specificities of giftedness), but the level of transparency involved in reporting effect sizes in the results is strictly necessary and useful for future research.

When effect sizes are available in this literature, they tend to be systematically very small. For the results synthesized in this literature review, the median percentage of variance explained by giftedness was 4.23% (min = 0%; max = 65.61%). As it stands, such results suggest negligible or no effect of giftedness in most of the domains investigated in this paper. This contributes to the conclusion that none of the domains considered here can be used as a diagnostic criterion for giftedness: apart from the fact that results tend to be unstable, giftedness generally does not explain enough variation in the measures to create a meaningful separation between gifted and non-gifted groups. It is also worth recalling that statistical power in a study depends on sample size and effect size. Considering the pre-existing findings on the topic and the generally very low effect sizes, future studies on non-cognitive characteristics of giftedness should favor large sample sizes to put forth robust findings.

#### 4.2.4. Measurement Tools

A weakness in research on non-cognitive characteristics of the intellectually gifted is the overall lack of consistency when comparing results from different studies. Part of the problem may stem from the use of various measures and instruments that do not assess the same facets/components of a given concept. This statement applies to meta-analytic studies as well as to integrative reviews of works on anxiety, humor, moral development, and emotional intelligence.

Another issue is that measurement tools sometimes display poor psychometric qualities. The extent to which results can be generalized to a larger group of individuals depends on the reliability and validity of the measurement (Scholwinski and Reynolds 1985). Moreover, the psychometric qualities of a measure depend on its context of use: reliability and validity are the properties of a particular scale in a particular population, not a property of the scale itself. In principle, the psychometric qualities of tests initially designed to assess non-gifted children and adolescents should therefore be examined (either before or after performing the study of interest) in a sample of intellectually gifted participants to verify that the structure and the qualities of the instrument are the same for the gifted and non-gifted groups. In practice, even researchers who do not want to engage in a full psychometric analysis (e.g., examination of measurement invariance across populations) can at least check the factor structure and the internal consistency of their instrument (e.g., Cronbach’s alpha) systematically and with little effort.

It is also worth mentioning the difference between self-report and other-report measures. Some studies rely exclusively on parents and teacher-rated assessments. These methods can provide valuable information, but they are not suitable for all situations. Halo effects and confirmation bias are likely in this field of study, with many teachers and parents having firmly entrenched preconceptions of what gifted children are like (Lee-Hammond 1999; Neumeister et al. 2007; and Tavani et al. 2009). One way to overcome this difficulty is to use mixed-design studies whenever possible, including both self-report and other-report data (e.g., see Schuler 2000).

#### 4.2.5. Study Designs

A major interest of research exploring the non-cognitive characteristics of intellectually gifted children and adolescents is to identify specificities of intellectual giftedness. Two comparison methods were used in the majority of studies covered in the present review: comparison to a control group or to normative data. The use of large control groups to investigate differences between intellectually gifted and non-gifted participants is, in our opinion, always the preferred alternative because it enables comparisons between participants experiencing similar environments (culture, socioeconomic level, education, etc.) and sharing a common research setting. It also appears to be the most commonly used method: in this literature review, nearly 70% of studies (excluding meta-analyses) included a control group in their study design, with a median size of 117 participants (min = 20; max = 10,096). A few studies in the field compared the outcomes of gifted participants to pre-existing norms (22% of included studies approximately), which has the disadvantage of allowing for more confounding variables to bias the difference between gifted participants and the norm (such as the context in which the study is performed). Finally, a little more than 8% of the studies synthesized in this literature review did not include a control or comparison group, particularly in the field of overexcitability studies. While case studies are always useful to provide the impetus for further research, they should be treated very carefully in the context of gifted youth: generalization of case study results are difficult given the wide heterogeneity of contexts, designs, and profiles of gifted participants. Thus, there is a definite need for large-scale quantitative studies.

### 4.3. Strengths and Limitations of This Review

This review is the first integrative review of the literature focusing on the non-cognitive specificities of intellectual giftedness. It includes a large panel of 240 studies covering a total of 17 topics. This substantial number of research papers provides a solid groundwork for future research in the field. The cross-sectional nature of this literature review also benefits clinical practice and support for gifted youth. It provides an objective overview of the non-cognitive characteristics associated with intellectual giftedness. This paper contributes to debunking the myth of identifying intellectual giftedness based on the non-cognitive characteristics associated with it. There is a widespread belief that intellectual giftedness may be a vulnerability in many areas, such as mental health, socialization, and emotional management (Barbier et al. 2022; Baudson 2016; Bergold et al. 2021; Carman 2011; Manaster et al. 1994; Subotnik et al. 2011; Vialle et al. 2007; and Weyns et al. 2021). However, this is not in line with the scientific literature. These misconceptions are damaging because they could have a self-fulfilling effect (also referred to as the Pygmalion effect, Rosenthal 2010) throughout the development of children and adolescents, who could ultimately embrace these stereotypes unconsciously (Baudson and Preckel 2016; Bergold et al. 2021; and Freeman 2013). This work provides support to limit this deleterious effect as much as possible. Finally, another advantage of this review is that it covers the specialized literature on giftedness, but also makes links with the literature on intelligence in general, an approach not systematically adopted in this type of article.

Several methodological limitations inherent to the field of giftedness literature were already mentioned. Comparison between the different studies is therefore limited. Other limitations specific to this literature review should also be highlighted. The first is that we did not include grey literature (i.e., dissertations, conference proceedings, book chapters, and other unpublished data) in this literature review. A second element to consider is that there are also old and not very accessible studies on giftedness, which may not be well represented in this literature review despite our attempts not to limit our article searches to a defined period of time. Finally, the keyword searches carried out for this review are relatively limited by the sets of keywords considered. Indeed, we potentially missed some articles relevant to our research objective because they used different terms from ours to refer to the same idea. This bias would mainly affect subjects for which multiple meanings were reported, such as the use of a range of terms to refer to self-esteem (n.b. in such cases, studies may also use the same terms but not refer to exactly the same concept, considering two different terms to be interchangeable when this is not necessarily the case). The sets of keywords used could also be limited to address fields of literature that are very broad and difficult to define, such as the socialization issue. Indeed, the social sphere can be approached from different points of view (social abilities, socialization skills perceived by others, the appropriateness of social responses, etc.). For these reasons, this review is not totally exhaustive but succeeds in providing a valuable overview of the literature.

## 5. Conclusions and Directions for Future Studies

This review of the literature examined various non-cognitive psychological dimensions that contribute to the stereotype of intellectual giftedness in the general public, and that could play a role in the clinical and educational support of gifted young people. Despite preconceptions common in public opinion, the results gathered in this paper do not show very substantial differences between gifted and non-gifted youth, and when differences do exist, there is little homogeneity across subjects and studies. In other words, intellectually gifted children and adolescents can present average differences in certain domains to a certain extent, but these differences do not form a coherent profile that would be discriminating or stable from one gifted individual to another. The scientific literature on this subject is based on small effect sizes that suggest that there are few major differences between the non-cognitive characteristics of gifted and non-gifted youth. When specificities are observed, they are predominantly positive. This does not mean that all intellectually gifted youth are fully protected from psychological, social and emotional difficulties, but it serves to show that the common view of all gifted individuals having socio-emotional difficulties is largely misguided. 

Existing research is, however, plagued by methodological weaknesses that should be carefully considered in future research. Firstly, this systematic review of the literature highlights the importance of gradually bringing coherence to the scientific approach to giftedness. The multiple, and sometimes subjective, definitions of giftedness make it difficult to compare results, lest the characteristics of gifted subpopulations be overlooked. In the field of intellectual giftedness, the use of measures of fluid intelligence (i.e., the estimation of the general intelligence factor) appears to be a valuable response to this problem. Indeed, it offers an alternative approach that is relatively free from socio-economic and cultural influences, reliable, valid, and easy to reproduce from one study to the next. The replication of studies in different educational and living contexts also seems to be a crucial issue for the generalization of future results to the population of gifted children and adolescents. Cross-cultural studies could make a major contribution to giftedness research, insofar as they could highlight the potential effects of context as a confounding variable. Indeed, it is essential to address in the future the persistent discrepancies between the findings in the literature on non-cognitive characteristics associated with giftedness. Taking into account the variability of environments in which gifted youth evolve appears to be a potential key to better understanding this discrepancy between studies. Ultimately, a more coherent picture of giftedness and its associated characteristics should emerge.

## Figures and Tables

**Table 1:** Summary of the different keywords used in the search procedure for each section of the literature review.

Sections	Keywords
Anxiety	(*anxiety* [Title]) NOT (*test* [Title]) (*test anxiety* [Title]) (*perfectionism* [Title])
Mood disorders	(*depression* [Title] OR *depressive disorder* [Title]) (*suicidal ideation* [Title]) (*mood disorders* [Title] OR *schizophrenia* [Title] OR *bipolar disorder* [Title] OR *mental health* [Title])
Well-being and quality of life	(*academic performance* [Title] OR *achievement* [Title] OR *school performance* [Title] OR *success* [Title] OR *social status* [Title]) (*life expectancy* [Title] OR *medical disorder* [Title] OR *physical health* [Title]) (*life satisfaction* [Title] OR *well-being* [Title] OR *happiness* [Title])
Social relationships	(*socialization* [Title] OR *social skills* [Title] OR *social abilities* [Title])
Self-esteem	(*self-esteem* [Title] OR *self-worth* [Title])
Humor	(*humor* [Title] OR *humour* [Title] OR *jokes* [Title])
Interests/hobbies	(*interests* [Title] OR *hobbies* [Title] OR *spare time* [Title])
Moral development	(*moral development* [Title] OR *moral judgement* [Title] OR *moral reasoning* [Title])
Leadership	(*leadership* [Title])
Emotional intelligence	(*emotional intelligence* [Title])
Overexcitabilities	(*overexcitabilities* [Title])

## Data Availability

No new data were created or analyzed in this study. Data sharing is not applicable to this article.

## Supplementary Materials

The following supporting information can be downloaded at: https://www.mdpi.com/article/10.3390/jintelligence11070141/s1, Tables S1–S17: Summary tables of methodological aspects and effect sizes for each section.

## References

[B1-jintelligence-11-00141] Abdulla Alabbasi Ahmed M., Ayoub Alaa Eldin A., Ziegler Albert (2020). Are gifted students more emotionally intelligent than their non-gifted peers? A meta-analysis. High Ability Studies.

[B2-jintelligence-11-00141] Acar Selcuk, Sen Sedat, Cayirdag Nur (2016). Consistency of the performance and nonperformance methods in gifted identification: A multilevel meta-analytic review. Gifted Child Quarterly.

[B3-jintelligence-11-00141] Ackerman Phillip L., Heggestad Eric D. (1997). Intelligence, personality, and interests: Evidence for overlapping traits. Psychological Bulletin.

[B4-jintelligence-11-00141] Adams-Byers Jan, Whitsell Sara Squiller, Moon Sidney M. (2004). Gifted students’ perceptions of the academic and social/emotional effects of homogeneous and heterogeneous grouping. Gifted Child Quarterly.

[B5-jintelligence-11-00141] Affrunti Nicholas W., Woodruff-Borden Janet (2014). Perfectionism in Pediatric Anxiety and Depressive Disorders. Clinical Child and Family Psychology Review.

[B6-jintelligence-11-00141] Alexopoulou Arhondoula, Batsou Alexandra, Drigas Athanasios (2019). Resilience and Academic Underachievement in Gifted Students: Causes, Consequences and Strategic Methods of Prevention and Intervention. International Journal of Online & Biomedical Engineering.

[B7-jintelligence-11-00141] Alias Aliza, Rahman Saemah, Majid Rosadah Abd, Yassin Siti Fatimah Mohd (2013). Dabrowski’s overexcitabilities profile among gifted students. Asian Social Science.

[B8-jintelligence-11-00141] Alnabhan Mousa (2011). How does Moral Judgement Change with Age and Giftedness?. Gifted and Talented International.

[B9-jintelligence-11-00141] Alsaker Françoise D. (1989). School Achievement, Perceived Academic Competence and Global Self-esteem 1. School Psychology International.

[B10-jintelligence-11-00141] Ambrose Don, Cross Tracy. L. (2009). Morality, Ethics, and Gifted Minds.

[B11-jintelligence-11-00141] Anderson Brittany N. (2020). “See me, see us”: Understanding the intersections and continued marginalization of adolescent gifted black girls in US classrooms. Gifted Child Today.

[B12-jintelligence-11-00141] Antonakis John, Simonton Dean Keith, Wai Jonathan (2019). Intelligence and leadership. Leader Thinking Skills.

[B13-jintelligence-11-00141] American Psychological Association (2021). Anxiety. https://dictionary.apa.org/anxiety.

[B14-jintelligence-11-00141] Asendorpf Jens B., van Aken Marcel A. G. (1994). Traits and Relationship Status: Stranger versus. Child Development.

[B15-jintelligence-11-00141] Ash Chris, Huebner E. Scott (1998). Life satisfaction reports of gifted middle-school children. School Psychology Quarterly.

[B16-jintelligence-11-00141] Aubry Alexandre, Gonthier Corentin, Bourdin Béatrice (2021). Explaining the high working memory capacity of gifted children: Contributions of processing skills and executive control. Acta Psychologica.

[B17-jintelligence-11-00141] Bailey Carrie Lynn (2011). An examination of the relationships between ego development, Dabrowski’s theory of positive disintegration, and the behavioral characteristics of gifted adolescents. Gifted Child Quarterly.

[B18-jintelligence-11-00141] Bain Sherry K., Bell Sherry Mee (2004). Social self-concept, social attributions, and peer relationships in fourth, fifth, and sixth graders who are gifted compared to high achievers. Gifted Child Quarterly.

[B19-jintelligence-11-00141] Bakar Abu Yazid Abu (2020). Effects of character education program on gifted and talented students’ self-esteem. Journal of Gifted Education and Creativity.

[B20-jintelligence-11-00141] Baker Jean A. (1995). Depression and Suicidal Ideation Among Academically Gifted Adolescents. Gifted Child Quarterly.

[B21-jintelligence-11-00141] Ball Christopher, Mann Leon, Stamm Cecily (1994). Decision-making abilities of intellectually gifted and non-gifted children. Australian Journal of Psychology.

[B22-jintelligence-11-00141] Barbier Katelijne, Struyf Elke, Donche Vincent (2022). Teachers’ beliefs about and educational practices with high-ability students. Teaching and Teacher Education.

[B23-jintelligence-11-00141] Barnett Lynn A., Fiscella Joan (1985). A Child By Any Other Name… A Comparison of the Playfulness of Gifted and Nongifted Children. Gifted Child Quarterly.

[B24-jintelligence-11-00141] Barone Diane, Barone Rebecca (2018). What is justice? Gifted students’ meaning making. The Reading Teacher.

[B25-jintelligence-11-00141] Bartell Nina P., Reynolds William M. (1986). Depression and self-esteem in academically gifted and nongifted children: A comparison study. Journal of School Psychology.

[B26-jintelligence-11-00141] Battle James, Blowers Tom (1982). A longitudinal comparative study of the self-esteem of students in regular and special education classes. Journal of Learning Disabilities.

[B27-jintelligence-11-00141] Baudson Tanja G. (2016). The mad genius stereotype: Still alive and well. Frontiers in Psychology.

[B28-jintelligence-11-00141] Baudson Tanja G., Preckel Franzis (2016). Teachers’ conceptions of gifted and average-ability students on achievement-relevant dimensions. Gifted Child Quarterly.

[B29-jintelligence-11-00141] Baumeister Roy F. (1997). Identity, self-concept, and self-esteem: The self lost and found. Handbook of Personality Psychology.

[B30-jintelligence-11-00141] Baumeister Roy F., Campbell Jennifer D., Krueger Joachim I., Vohs Kathleen D. (2003). Does high self-esteem cause better performance, interpersonal success, happiness, or healthier lifestyles?. Psychological Science in the Public Interest.

[B31-jintelligence-11-00141] Beer John (1991). Depression, General Anxiety, Test Anxiety, and Rigidity of Gifted Junior High and High School Children. Psychological Reports.

[B32-jintelligence-11-00141] Beißert Hanna M., Hasselhorn Marcus (2016). Individual differences in moral development: Does intelligence really affect children’s moral reasoning and moral emotions?. Frontiers in Psychology.

[B33-jintelligence-11-00141] Bergen Doris (2009). Gifted children’s humor preferences, sense of humor, and Comprehension of riddles. International Journal of Humor Research.

[B34-jintelligence-11-00141] Bergold Sebastian, Hastall Matthias Ricarda, Steinmayr Ricarda (2021). Do mass media shape stereotypes about intellectually gifted individuals? Two experiments on stigmatization effects from biased newspaper reports. Gifted Child Quarterly.

[B35-jintelligence-11-00141] Bergold Sebastian, Wirthwein Linda, Rost Detlef H., Steinmayr Ricarda (2015). Are gifted adolescents more satisfied with their lives than their non-gifted peers?. Frontiers in Psychology.

[B36-jintelligence-11-00141] Bergold Sebastian, Wirthwein Linda, Steinmayr Ricarda (2020). Similarities and Differences Between Intellectually Gifted and Average-Ability Students in School Performance, Motivation, and Subjective Well-Being. Gifted Child Quarterly.

[B37-jintelligence-11-00141] Bénony Hervé, Van DeElst Delphine, Chahraoui Khadija, Bénony Christelle, Marnier Jean-Paul (2007). Lien entre dépression et estime de soi scolaire chez les enfants intellectuellement précoces. L’Encéphale.

[B38-jintelligence-11-00141] Bianchi Ivana, Canestrari Carla, Roncoroni Anna Maria, Burro Roberto, Branchini Erika, Savardi Ugo (2017). The effects of modulating contrast in verbal irony as a cue for giftedness. Humor.

[B39-jintelligence-11-00141] Bieling Peter J., Israeli Anne L., Antony Martin M. (2004). Is Perfectionism Good, Bad, Or Both? Examining Models of the Perfectionism Construct. Personality and Individual Differences.

[B40-jintelligence-11-00141] Blyth Dale A., Traeger Carol M. (1983). The self-concept and self-esteem of early adolescents. Theory into Practice.

[B41-jintelligence-11-00141] Bouchard Lorraine L. (2004). An instrument for the measure of Dabrowskian overexcitabilities to identify gifted elementary students. Gifted Child Quarterly.

[B42-jintelligence-11-00141] Bouchet Nicole, Falk R. Frank (2001). The relationship among giftedness, gender, and overexcitability. Gifted Child Quarterly.

[B43-jintelligence-11-00141] Brasseur Sophie, Grégoire Jacques (2010). L’intelligence émotionnelle–trait chez les adolescents à haut potentiel: Spécificités et liens avec la réussite scolaire et les compétences sociales. Enfance.

[B44-jintelligence-11-00141] Breard Nancy Starr (1995). Exploring a Different Way to Identify Gifted African American Students. Ph.D. thesis.

[B45-jintelligence-11-00141] Brody Linda E., Benbow Camilla P. (1986). Social and emotional adjustment of adolescents extremely talented in verbal or mathematical reasoning. Journal of Youth and Adolescence.

[B46-jintelligence-11-00141] Bronk Kendall Cotton, Finch W. Holmes, Talib Tasneem L. (2010). Purpose in life among high ability adolescents. High Ability Studies.

[B47-jintelligence-11-00141] Bücker Susanne, Nuraydin Sevim, Simonsmeier Bianca A., Schneider Michael, Luhmann Maike (2018). Subjective well-being and academic achievement: A meta-analysis. Journal of Research in Personality.

[B48-jintelligence-11-00141] Bull Catherine Anne (1997). Perfectionism and Self-Esteem in Early Adolescence. Ph.D. Thesis.

[B49-jintelligence-11-00141] Calero M. Dolores, Belen Garcia-Martin-M, Robles M. Auxiliadora (2011). Learning Potential in high IQ children: The contribution of dynamic assessment to the identification of gifted children. Learning and Individual Differences.

[B50-jintelligence-11-00141] Carman Carol A. (2011). Stereotypes of giftedness in current and future educators. Journal for the Education of the Gifted.

[B51-jintelligence-11-00141] Carman Carol A. (2013). Comparing apples and oranges: Fifteen years of definitions of giftedness in research. Journal of Advanced Academics.

[B52-jintelligence-11-00141] Casino-García Anna Maria, García-Pérez Josefa, Llinares-Insa Lucía Immaculada (2019). Subjective Emotional Well-Being, Emotional Intelligence, and Mood of Gifted vs. Unidentified Students: A Relationship Model. International Journal of Environmental Research and Public Health.

[B53-jintelligence-11-00141] Casino-García Anna Maria, Llopis-Bueno Maria José, Llinares-Insa Lucía Immaculada (2021). Emotional intelligence profiles and self-esteem/self-concept: An analysis of relationships in gifted students. International Journal of Environmental Research and Public Health.

[B54-jintelligence-11-00141] Ceci Stephe J., Williams Wendy M. (1997). Schooling, intelligence, and income. American Psychologist.

[B55-jintelligence-11-00141] Cernova Lubova (2005). Aggression and anxiety of intellectually gifted Russian adolescents in Latvia. Baltic Journal of Psychology.

[B56-jintelligence-11-00141] Chan Lorna K. S. (1988). The perceived competence of intellectually talented students. Gifted Child Quarterly.

[B57-jintelligence-11-00141] Chan David W. (2002). Perceptions of giftedness and self-concepts among junior secondary students in Hong Kong. Journal of Youth and Adolescence.

[B58-jintelligence-11-00141] Chan David W. (2003). Dimensions of emotional intelligence and their relationships with social coping among gifted adolescents in Hong Kong. Journal of Youth and Adolescence.

[B59-jintelligence-11-00141] Chan David W. (2008). Perfectionism and the Striving for Excellence. Educational Research Journal.

[B60-jintelligence-11-00141] Chan David W. (2010). Healthy and unhealthy perfectionists among academically gifted Chinese students in Hong Kong: Do different classification schemes make a difference?. Roeper Review.

[B61-jintelligence-11-00141] Cheng Helen, Furnham Adrian (2014). The associations between parental socio-economic conditions, childhood intelligence, adult personality traits, social status and mental well-being. Social Indicators Research.

[B62-jintelligence-11-00141] Chiu Lian-Hwang (1990). Self-Esteem of gifted, normal, and mild mentally handicapped children. Psychology in the Schools.

[B63-jintelligence-11-00141] Chmiel Magda, Brunner Martin, Keller Ulrich, Schalke Daniela, Wrulich Marius, Martin Romain (2012). Does childhood general cognitive ability at age 12 predict subjective well-being at age 52?. Journal of Research in Personality.

[B64-jintelligence-11-00141] Chovan William, Freeman Nancy L. (1993). Moral reasoning and personality components in gifted and average students. Perceptual and Motor Skills.

[B65-jintelligence-11-00141] Christensen Alexander P., Silvia Paul J., Nusbaum Emily C., Beaty Roger E. (2018). Clever people: Intelligence and humor production ability. Psychology of Aesthetics, Creativity, and the Arts.

[B66-jintelligence-11-00141] Cohen LeoNora M. (1989). Understanding the interests and themes of the very young gifted child. Gifted Child Today Magazine.

[B67-jintelligence-11-00141] Cohen Robert, Duncan Melissa, Cohen Sheila L. (1994). Classroom Peer Relations of Children Participating in a Pull-Out Enrichment Program. Gifted Child Quarterly.

[B68-jintelligence-11-00141] Colangelo Nicholas, Kelly Kevin R., Schrepfer Ray M. (1987). A comparison of gifted, general, and special learning needs students on academic and social self-concept. Journal of Counseling & Development.

[B69-jintelligence-11-00141] Coleman Laurence J., Cross Tracy L. (1988). Is being gifted a social handicap?. Journal for the Education of the Gifted.

[B70-jintelligence-11-00141] Coleman Laurence J., Cross Tracy L. (2014). Is Being Gifted a Social Handicap?. Journal for the Education of the Gifted.

[B71-jintelligence-11-00141] Cook Fallon, Hippmann Danielle, Omerovic Emina (2020). The sleep and mental health of gifted children: A prospective, longitudinal, community cohort study. Gifted and Talented International.

[B72-jintelligence-11-00141] Coopersmith Stanley (1967). The Antecedents of Self-Esteem.

[B73-jintelligence-11-00141] Cornell Dewey G., Grossberg Ingrid W. (1987). Family environment and personality adjustment in gifted program children. Gifted Child Quarterly.

[B74-jintelligence-11-00141] Corso Sean (2001). Emotional Intelligence in Adolescents: How it Relates to Giftedness. Ph.D. Thesis.

[B75-jintelligence-11-00141] Courtinat-Camps Amélie, Massé Line, Léonardis Myriam de (2012). Self-portraits and self-esteem in French gifted students. Handbook on Psychology of Self-Esteem.

[B76-jintelligence-11-00141] Cross Tracy (1996). Social/emotional needs: Examining claims about gifted children and suicide. Gifted Child Today.

[B77-jintelligence-11-00141] Cross Jennifer Riedl, Cross Tracy L. (2015). Clinical and Mental Health Issues in Counseling the Gifted Individual. Journal of Counseling & Development.

[B78-jintelligence-11-00141] Cross Tracy L., Swiatek Mary Ann (2009). Social coping among academically gifted adolescents in a residential setting: A longitudinal study. Gifted Child Quarterly.

[B79-jintelligence-11-00141] Cross Tracy L., Cassady Jerrell C., Miller Kimberly A. (2006). Suicidal ideation and psychological type in gifted adolescents. Gifted Child Quarterly.

[B80-jintelligence-11-00141] Cross Tracy L., Cassady Jerrell C., Dixon Felicia A., Adams Cheryll M. (2008). The Psychology of Gifted Adolescents as Measured by the MMPI-A. Gifted Child Quarterly.

[B81-jintelligence-11-00141] Cunningham Anne (1962). Relation of sense of humor to intelligence. The Journal of Social Psychology.

[B82-jintelligence-11-00141] Czeschlik Tatiana, Rost Detlef H. (1994). Socio-emotional adjustment in elementary school boys and girls: Does giftedness make a difference?. Roeper Review.

[B83-jintelligence-11-00141] Dai David Yun, Rinn Anne N. (2008). The Big-Fish-Little-Pond Effect: What Do We Know and Where Do We Go from Here?. Educational Psychology Review.

[B84-jintelligence-11-00141] Daly Michael, Egan Mark, O’Reilly Fionnuala (2015). Childhood general cognitive ability predicts leadership role occupancy across life: Evidence from 17,000 cohort study participants. The Leadership Quarterly.

[B85-jintelligence-11-00141] D’Amico Antonella, Cardaci Maurizio (2003). Relations among perceived self-efficacy, self-esteem, and school achievement. Psychological Reports.

[B86-jintelligence-11-00141] Dauber Susan L., Benbow Camilla P. (1990). Aspects of Personality and Peer Relations of Extremely Talented Adolescents. Gifted Child Quarterly.

[B87-jintelligence-11-00141] Dean Raymond S. (1977). Effects of self-concept on learning with gifted children. The Journal of Educational Research.

[B88-jintelligence-11-00141] Deary Ian J., Batty G. David, Pattie Alison, Gale Catharine R. (2008). More Intelligent, More Dependable Children Live Longer. Psychological Science.

[B89-jintelligence-11-00141] Deary Ian J., Strand Steve, Smith Pauline, Fernandes Crs (2007). Intelligence and educational achievement. Intelligence.

[B90-jintelligence-11-00141] Deku Prosper (2013). Teacher nomination of gifted and talented children: A study of basic and senior high schools in the Central Region of Ghana. Intelligence.

[B91-jintelligence-11-00141] Demetriou Andreas, Kazi Smaragda, Makris Nikolaos, Spanoudis George (2020). Cognitive ability, cognitive self-awareness, and school performance: From childhood to adolescence. Intelligence.

[B92-jintelligence-11-00141] Derryberry W. Pitt, Barger Brian (2008). Do contributors to intellect explain the moral judgment abilities of gifted youth?. Gifted Child Quarterly.

[B93-jintelligence-11-00141] Derryberry W. Pitt, Wilson Travis, Snyder Hannah, Norman Tony, Barger Brian (2005). Moral judgment developmental differences between gifted youth and college students. Journal of Secondary Gifted Education.

[B94-jintelligence-11-00141] Di Giunta Laura, Alessandri Guido, Gerbino Maria, Kanacri Paula Luengo, Zuffiano Antonio, Caprara Gian Vittorio (2013). The determinants of scholastic achievement: The contribution of personality traits, self-esteem, and academic self-efficacy. Learning and Individual Differences.

[B95-jintelligence-11-00141] Diseth Åge, Meland Eivind, Breidablik Hans Johan (2014). Self-beliefs among students: Grade level and gender differences in self-esteem, self-efficacy and implicit theories of intelligence. Learning and Individual Differences.

[B96-jintelligence-11-00141] Eklund Katie, Tanner Nick, Stoll Katie, Anway Leslie (2015). Identifying emotional and behavioral risk among gifted and nongifted children: A multi-gate, multi-informant approach. School Psychology Quarterly.

[B97-jintelligence-11-00141] Fanaj Naim, Mustafa Sevim (2021). Examining self-esteem and family characteristics (birth order, family satisfaction, educational level, socio-economic status) among nominated children as gifted in Kosovo. Studime Sociale.

[B98-jintelligence-11-00141] Fang Junyan, Huang Xitong, Zhang Minqiang, Huang Feifei, Li Zhe, Yuan Qiting (2018). The big-fish-little-pond effect on academic self-concept: A meta-analysis. Frontiers in Psychology.

[B99-jintelligence-11-00141] Faouri Faizeh Abdel-Kaveem (1998). The Impact of Learning Disabilities and Giftedness on the Self-Esteem of Students. Ph.D. Thesis.

[B100-jintelligence-11-00141] Feldhusen John F., Klausmeier Herbert J. (1962). Anxiety, Intelligence, and Achievement in Children of Low, Average, and High Intelligence. Child Development.

[B101-jintelligence-11-00141] Field Tiffany, Harding Jeff, Yando Regina, Gonzalez Ketty, Lasko David, Bendell Debra, Marks Carol (1998). Feeling and attitude of gifted students. Adolescence.

[B102-jintelligence-11-00141] Filosa Lorenzo, Alessandri Guido, Robins Richard W., Pastorelli Concetta (2022). Self-esteem development during the transition to work: A 14-year longitudinal study from adolescence to young adulthood. Journal of Personality.

[B103-jintelligence-11-00141] Foley-Nicpon Megan, Rickels Heather, Assouline Susan G., Richards Allison (2012). Self-esteem and self-concept examination among gifted students with ADHD. Journal for the Education of the Gifted.

[B104-jintelligence-11-00141] Forsyth Patricia (1987). A study of self-concept, anxiety, and security of children in gifted, French immersion, and regular classes. Canadian Journal of Counselling and Psychotherapy/Revue Canadienne de Counseling et de Psychothérapie.

[B105-jintelligence-11-00141] França-Freitas Maria Luiza P., Del Prette Amir, Del Prette Zilda A. P. (2014). Social skills of gifted and talented children. Estudos de Psicologia.

[B106-jintelligence-11-00141] Francis Rosanna, Hawes David J., Abbott Maree (2015). Intellectual Giftedness and Psychopathology in Children and Adolescents. Exceptional Children.

[B107-jintelligence-11-00141] Francis Rosanna, Hawes David J., Abbott Maree J., Costa Daniel S. J. (2018). Cognitive mechanisms for worry in early adolescence: Re-examining the role of high verbal intelligence. Personality and Individual Differences.

[B108-jintelligence-11-00141] Freeman Joan (2013). Gifted Children Grown Up.

[B109-jintelligence-11-00141] Gagné François (2005). From gifts to talents. Conceptions of Giftedness.

[B110-jintelligence-11-00141] Galloway Briar, Porath Marion (1997). Parent and teacher views of gifted children’s social abilities. Roeper Review.

[B111-jintelligence-11-00141] Gallucci Nicholas T. (1988). Emotional Adjustment of Gifted Children. Gifted Child Quarterly.

[B112-jintelligence-11-00141] Gallucci Nicholas T., Middleton George, Kline Adam (1999). Intellectually superior children and behavioral problems and competence. Roeper Review.

[B113-jintelligence-11-00141] Garland Ann F., Zigler Dward (1999). Emotional and behavioral problems among highly intellectually gifted youth. Roeper Review.

[B114-jintelligence-11-00141] Gauvrit Nicolas (2014). Précocité intellectuelle: Un champ de recherche miné. ANAE. Approche Neuropsychologique des Apprentissages chez l’Enfant.

[B115-jintelligence-11-00141] Geake John G. (2008). High abilities at fluid analogizing: A cognitive neuroscience construct of giftedness. Roeper Review.

[B116-jintelligence-11-00141] Gere Douglas R., Capps Steve C., Mitchell D. Wayne, Grubbs Erin (2009). Sensory sensitivities of gifted children. American Journal of Occupational Therapy.

[B117-jintelligence-11-00141] Ghobary Bagher, Hejazi Masoud (2007). Assertiveness, self-esteem and academic achievement in gifted and normal students. Psychological Science: Research, Theory and Future Directions.

[B118-jintelligence-11-00141] Giofrè David, Borella Erika, Mammarella Irene Cristina (2017). The relationship between intelligence, working memory, academic self-esteem, and academic achievement. Journal of Cognitive psychology.

[B119-jintelligence-11-00141] Goetz Thomas, Preckel Franzis, Zeidner Moshe, Schleyer Esther (2008). Big fish in big ponds: A multilevel analysis of test anxiety and achievement in special gifted classes. Anxiety, Stress & Coping.

[B120-jintelligence-11-00141] Goleman Daniel (1995). Emotional Intelligence.

[B121-jintelligence-11-00141] Greenberg Jeff, Solomon Sheldon, Pyszczynski Tom, Rosenblatt Abram, Burling John, Lyon Deborah, Simon Linda, Pinel Elizabeth (1992). Why do people need self-esteem? Converging evidence that self-esteem serves an anxiety-buffering function. Journal of Personality and Social Psychology.

[B122-jintelligence-11-00141] Greengross Gil, Miller Geoffrey (2011). Humor ability reveals intelligence, predicts mating success, and is higher in males. Intelligence.

[B123-jintelligence-11-00141] Gross Candace M., Rinn Anne N., Jamieson Kelly M. (2007). Gifted adolescents’ overexcitabilities and self-concepts: An analysis of gender and grade level. Roeper Review.

[B124-jintelligence-11-00141] Guez Ava, Peyre Hugo, Cam Marion Le, Gauvrit Nicolas, Ramus Franck (2018). Are high-IQ students more at risk of school failure?. Intelligence.

[B125-jintelligence-11-00141] Guénolé Fabian, Louis Jacqueline, Creveuil Christian, Montlahuc Claire, Baleyte J-M., Fourneret Pierre, Revol Olivier (2013). Étude transversale de l’anxiété trait dans un groupe de 111 enfants intellectuellement surdoués. L’Encéphale.

[B126-jintelligence-11-00141] Guignard Jacques-Henri, Jacquet Anne-Yvonne, Lubart Todd I. (2012). Perfectionism and Anxiety: A Paradox in Intellectual Giftedness?. PLoS ONE.

[B127-jintelligence-11-00141] Guignard Jacques-Henri, Bacro Fabien, Guimard Philippe (2021). School life satisfaction and peer connectedness of intellectually gifted adolescents in France: Is there a labeling effect?. New Directions for Child and Adolescent Development.

[B128-jintelligence-11-00141] Guignard Jacques-Henri, Zenasni Franck F. (2004). Les caractéristiques émotionnelles des enfants à haut potentiel. Psychologie Française.

[B129-jintelligence-11-00141] Guthrie Kate H. (2019). “ Nothing Is Ever Easy”: Parent Perceptions of Intensity in Their Gifted Adolescent Children. The Qualitative Report.

[B130-jintelligence-11-00141] Hansford Brian C., Hattie John A. (1982). The relationship between self and achievement/performance measures. Review of Educational Research.

[B131-jintelligence-11-00141] Hanushek Eric A. (2021). Addressing cross-national generalizability in educational impact evaluation. International Journal of Educational Development.

[B132-jintelligence-11-00141] Hanushek Eric A., Piopiunik Marc, Wiederhold Simon (2019). The value of smarter teachers international evidence on teacher cognitive skills and student performance. Journal of Human Resources.

[B133-jintelligence-11-00141] Harper Amanda, Cornish Linley, Smith Susen, Merrotsy Peter (2017). Through the Dąbrowski lens: A fresh examination of the Theory of Positive Disintegration. Roeper Review.

[B134-jintelligence-11-00141] Harrison Gregory E., Van Haneghan James P. (2011). The gifted and the shadow of the night: Dabrowski’s overexcitabilities and their correlation to insomnia, death anxiety, and fear of the unknown. Journal for the Education of the Gifted.

[B135-jintelligence-11-00141] Harter Susan (1993). Causes and consequences of low self-esteem in children and adolescents. Self-Esteem: The Puzzle of Low Self-Regard.

[B136-jintelligence-11-00141] Hauck William E., Thomas John W. (1972). The Relationship of Humor to Intelligence, Creativity, and Intentional and Incidental Learning. The Journal of Experimental Education.

[B137-jintelligence-11-00141] Hembree Ray (1988). Correlates, Causes, Effects, and Treatment of Test Anxiety. Review of Educational Research.

[B138-jintelligence-11-00141] Hewitt Paul L., Flett Gordon L. (1991). Perfectionism in the self and social contexts: Conceptualization, assessment, and association with psychopathology. Journal of Personality and Social Psychology.

[B139-jintelligence-11-00141] Hill Andrew P., Curran Thomas (2015). Multidimensional Perfectionism and Burnout. Personality and Social Psychology Review.

[B140-jintelligence-11-00141] Hoffman Seymour (1977). Intelligence and the development of moral judgment in children. The Journal of Genetic Psychology.

[B141-jintelligence-11-00141] Hoffman Brian J., Woehr David J., Maldagen-Youngjohn Robyn, Lyons Brian D. (2011). Great man or great myth? A quantitative review of the relationship between individual differences and leader effectiveness. Journal of Occupational and Organizational Psychology.

[B142-jintelligence-11-00141] Hollingworth Leta S. (1942). Children above 180 IQ, Stanford-Binet: Origin and Development.

[B143-jintelligence-11-00141] Holt Dan G., Willard-Holt Colleen (1995). An exploration of the relationship between humor and giftedness in students. Humor: International Journal of Humor Research.

[B144-jintelligence-11-00141] Howard-Hamilton Mary, Franks Bridget A. (1995). Gifted adolescents: Psychological behaviors, values, and developmental implications. Roeper Review.

[B145-jintelligence-11-00141] Huebner E. Scott, Alderman Gary L. (1993). Convergent and discriminant validation of a children’s life satisfaction scale: Its relationship to self- and teacher-reported psychological problems and school functioning. Social Indicators Research.

[B146-jintelligence-11-00141] Jackson P. Susan, Peterson Jean (2003). Depressive Disorder in Highly Gifted Adolescents. Journal of Secondary Gifted Education.

[B147-jintelligence-11-00141] Janos Paul M., Robinson Nancy M., Horowitz Frances Degen, O’Brien Marion (1985). Psychosocial development in intellectually gifted children. The Gifted and Talented: Developmental Perspectives.

[B148-jintelligence-11-00141] Janos Paul M., Fung Hellen C., Robinson Nancy M. (1985). Self-concept, self-esteem, and peer relations among gifted children who feel different. Gifted Child Quarterly.

[B149-jintelligence-11-00141] Judge Timothy A., Colbert Amy E., Ilies Remus (2004). Intelligence and leadership: A quantitative review and test of theoretical propositions. Journal of Applied Psychology.

[B150-jintelligence-11-00141] Kaiser Charles F., Berndt David J. (1985). Predictors of loneliness in the gifted adolescent. Gifted Child Quarterly.

[B151-jintelligence-11-00141] Karnes Frances A., Brown K. Eliot (1980). Moral development and the gifted: An initial investigation. Roeper Review.

[B152-jintelligence-11-00141] Kaya Fatih, Ogurlu Üzeyir (2015). The relationship among self-esteem, intelligence, and academic achievement. Journal of Human Sciences.

[B153-jintelligence-11-00141] Kellner Raphaela, Benedek Mathias (2016). The role of creative potential and intelligence for humor production. Psychology of Aesthetics, Creativity, and the Arts.

[B154-jintelligence-11-00141] Kendall Philip C., Norris Lesley A., Rabner Jonathan C., Crane Margaret E., Rifkin Lara S. (2020). Intolerance of Uncertainty and Parental Accommodation: Promising Targets for Personalized Intervention for Youth Anxiety. Current Psychiatry Reports.

[B155-jintelligence-11-00141] Kertz Sarah J., Peyton Joseph S. Bigda, Rosmarin David H., Björgvinsson Thröstur (2012). The importance of worry across diagnostic presentations: Prevalence, severity and associated symptoms in a partial hospital setting. Journal of Anxiety Disorders.

[B156-jintelligence-11-00141] Koenen Karestan C., Moffitt Terrie E., Roberts Andrea L., Martin Laurie T., Kubzansky Laura, Harrington HonaLee, Poulton Richie, Caspi Avshalom (2009). Childhood IQ and Adult Mental Disorders: A Test of the Cognitive Reserve Hypothesis. American Journal of Psychiatry.

[B157-jintelligence-11-00141] Kohlberg Lawrence, Hoffman Lois Wladis, Hoffman Martin L. (1964). Development of moral character and moral ideology. Review of Child Development Research.

[B158-jintelligence-11-00141] Kong Dejun Tony (2014). Mayer–Salovey–Caruso Emotional Intelligence Test (MSCEIT/MEIS) and overall, verbal, and nonverbal intelligence: Meta-analytic evidence and critical contingencies. Personality and Individual Differences.

[B159-jintelligence-11-00141] Kornblum Michelle, Ainley Mary (2005). Perfectionism and the Gifted: A Study of an Australian School Sample. International Education Journal.

[B160-jintelligence-11-00141] Košir Katja, Horvat Marina, Aram Urška, Jurinec Nina (2015). Is being gifted always an advantage? Peer relations and self-concept of gifted students. High Ability Studies.

[B161-jintelligence-11-00141] Kovacs Kristof, Conway Andrew R. A. (2016). Process overlap theory: A unified account of the general factor of intelligence. Psychological Inquiry.

[B162-jintelligence-11-00141] Kulik James A., Kulik Chen Lin C. (1992). Meta-analytic findings on grouping programs. Gifted Child Quarterly.

[B163-jintelligence-11-00141] Lea-Wood Sandra S., Clunies-Ross Graham (1995). Self-esteem of gifted adolescent girls in Australian schools. Roeper Review.

[B164-jintelligence-11-00141] Lee Seon-Young, Olszewski-Kubilius Paula (2006). The emotional intelligence, moral judgment, and leadership of academically gifted adolescents. Journal for the Education of the Gifted.

[B165-jintelligence-11-00141] Lee Seon-Young, Matthews Michael, Shin Jongho, Kim Myung-Seop (2020). Academically gifted adolescents’ social purpose. High Ability Studies.

[B166-jintelligence-11-00141] Lee-Hammond Libby (1999). Teachers’ conceptions of gifted and talented young children. High Ability Studies.

[B167-jintelligence-11-00141] Lehman Elyse Brauch, Erdwins Carol J. (1981). The Social and Emotional Adjustment of Young, Intellectually Gifted Children. Gifted Child Quarterly.

[B168-jintelligence-11-00141] Lehman H. Christian, Witty Paul A. (1927). The play behavior of fifty gifted children. Journal of Educational Psychology.

[B169-jintelligence-11-00141] Levy Jacob J., Plucker Jonathan A. (2003). Theory and practice: Assessing the psychological presentation of gifted and talented clients: A multicultural perspective. Counselling Psychology Quarterly.

[B170-jintelligence-11-00141] Lewis John, Adank Richard (1975). Intercorrelations Among Measures of Intelligence, Achievement, Self-Esteem, and Anxiety in Two Groups of Elementary School Pupils Exposed To Two Different Models of Instruction1. Educational and Psychological Measurement.

[B171-jintelligence-11-00141] Li Danfeng, Shi Jiannong (2019). Fluid intelligence, trait emotional intelligence and academic performance in children with different intellectual levels. High Ability Studies.

[B172-jintelligence-11-00141] Li Danfeng, Liu Tongran, Zhang Xingli, Wang Mingyi, Wang Di, Shi Jiannong (2017). Fluid intelligence, emotional intelligence, and the Iowa Gambling Task in children. Intelligence.

[B173-jintelligence-11-00141] Limont Wiesława, Dreszer-Drogorób Joanna, Bedyńska Sylwia, Śliwińska Katarzyna, Jastrzębska Dominika (2014). ‘Old wine in new bottles’? Relationships between overexcitabilities, the Big Five personality traits and giftedness in adolescents. Personality and Individual Differences.

[B174-jintelligence-11-00141] Liu Xiaoru, Kaplan Howard B., Risser Will (1992). Decomposing the reciprocal relationships between academic achievement and general self-esteem. Youth & Society.

[B175-jintelligence-11-00141] LoCicero Kenneth A., Ashby Jeffrey S. (2000). Multidimensional perfectionism in middle school age gifted students: A comparison to peers from the general cohort. Roeper Review.

[B176-jintelligence-11-00141] Lord Robert G., de Vader Christy L., Alliger George M. (1986). A meta-analysis of the relation between personality traits and leadership perceptions: An application of validity generalization procedures. Journal of Applied Psychology.

[B177-jintelligence-11-00141] Lovecky Deirdre V. (1992). Exploring social and emotional aspects of giftedness in children. Roeper Review.

[B178-jintelligence-11-00141] López Verónica, Sotillo Maria (2009). Giftedness and social adjustment: Evidence supporting the resilience approach in Spanish-speaking children and adolescents. High Ability Studies.

[B179-jintelligence-11-00141] MacCabe James H., Lambe Mats P., Cnattingius Sven, Sham Pak C., David Anthony S., Reichenberg Abraham, Murray Robin M., Hultman Christina M. (2010). Excellent school performance at age 16 and risk of adult bipolar disorder: National cohort study. British Journal of Psychiatry.

[B180-jintelligence-11-00141] MacCann Carolyn, Jiang Yixin, Brown Luke. E. R., Double Kit S., Bucich Micaela, Minbashian Amirali (2020). Emotional intelligence predicts academic performance: A meta-analysis. Psychological Bulletin.

[B181-jintelligence-11-00141] Manaster Guy J., Chan Jason C., Watt Celia, Wiehe James (1994). Gifted adolescents’ attitudes toward their giftedness: A partial replication. Gifted Child Quarterly.

[B182-jintelligence-11-00141] del Mar Ferradás Maria D. M., Freire Carlos, Núñez José Carlos, Regueiro Bibiana (2020). The relationship between self-esteem and achievement goals in university students: The mediating and moderating role of defensive pessimism. Sustainability.

[B183-jintelligence-11-00141] Marsh Herbert W., Craven Rhonda G. (2006). Reciprocal effects of self-concept and performance from a multidimensional perspective: Beyond seductive pleasure and unidimensional perspectives. Perspectives on Psychological Science.

[B184-jintelligence-11-00141] Marsh Herbert W., O’Mara Alison (2008). Reciprocal effects between academic self-concept, self-esteem, achievement, and attainment over seven adolescent years: Unidimensional and multidimensional perspectives of self-concept. Personality and Social Psychology Bulletin.

[B185-jintelligence-11-00141] Martin Laurie T., Burns Rachel M., Schonlau Matthias (2010). Mental Disorders Among Gifted and Nongifted Youth: A Selected Review of the Epidemiologic Literature. Gifted Child Quarterly.

[B186-jintelligence-11-00141] Masden Catherin A., Leung Olivia N., Shore Bruce M., Schneider Barry H., Udvari Stephen J. (2015). Social-perspective coordination and gifted adolescents’ friendship quality. High Ability Studies.

[B187-jintelligence-11-00141] Masten Ann S. (1986). Humor and competence in school-aged children. Child Development.

[B188-jintelligence-11-00141] Matthews Michael S. (2004). Leadership education for gifted and talented youth: A review of the literature. Journal for the Education of the Gifted.

[B189-jintelligence-11-00141] Matthews Gerald, Lin Jinchao, Zeidner Moshe, Roberts Richard D., Pfeiffer Steven I., Shaunessy-Dedrick Elizabeth, Foley-Nicpon Megan (2018). Emotional intelligence and giftedness. APA Handbooks in Psychology. APA Handbook of Giftedness and Talent.

[B190-jintelligence-11-00141] Mayer John D., Caruso David, Salovey Peter, Bar-On Reuven, Parker James D. A. (2000). Selecting a measure of emotional intelligence: The case for ability scales. The Handbook of Emotional Intelligence: Theory, Development, Assessment, and Application at Home, School, and in the Workplace.

[B191-jintelligence-11-00141] Mayer John D., Perkins M., Caruso Donna R., Salovey Peter (2001). Emotional intelligence and giftedness. Roeper Review.

[B192-jintelligence-11-00141] McBee Matthew T., Makel Matthew C. (2019). The quantitative implications of definitions of giftedness. AERA Open.

[B193-jintelligence-11-00141] McEwin C. Kenneth, Cross Arthur H. (1982). A comparative study of perceived victimization, perceived anonymity, self-esteem, and preferred teacher characteristics of gifted and talented and non-labeled early adolescents. The Journal of Early Adolescence.

[B194-jintelligence-11-00141] Mendaglio Sal (2008). Dabrowski’s Theory of Positive Disintegration.

[B195-jintelligence-11-00141] Mendaglio Sal (2012). Overexcitabilities and giftedness research: A call for a paradigm shift. Journal for the Education of the Gifted.

[B196-jintelligence-11-00141] Metha Arlene, McWhirter Ellen H. (1997). Suicide Ideation, Depression, and Stressful Life Events among Gifted Adolescents. Journal for the Education of the Gifted.

[B197-jintelligence-11-00141] Milgram Roberta M., Milgram Norman A. (1976). Personality Characteristics of Gifted Israeli Children. The Journal of Genetic Psychology.

[B198-jintelligence-11-00141] Mills Lane B. (2009). A meta-analysis of the relationship between emotional intelligence and effective leadership. Journal of Curriculum and Instruction.

[B199-jintelligence-11-00141] Missett Tracy C. (2013). Exploring the Relationship Between Mood Disorders and Gifted Individuals. Roeper Review.

[B200-jintelligence-11-00141] Mouchiroud Christophe (2004). Haut potentiel intellectuel et développement social. Psychologie Française.

[B201-jintelligence-11-00141] Moyano Nieves, Quílez-Robres Alberto, Pascual Alejandra Cortés (2020). Self-esteem and motivation for learning in academic achievement: The mediating role of reasoning and verbal fluidity. Sustainability.

[B202-jintelligence-11-00141] Muammar Omar M. (2013). The differences between intellectually gifted and average students on a set of leadership competencies. Gifted Education International.

[B203-jintelligence-11-00141] Mueller Christian E., Winsor Denise L. (2018). Depression, Suicide, and Giftedness: Disentangling Risk Factors, Protective Factors, and Implications for Optimal Growth. Handbook of Giftedness in Children.

[B204-jintelligence-11-00141] Narváez Darcia (1993). High achieving students and moral judgment. Journal for the Education of the Gifted.

[B205-jintelligence-11-00141] Neff Walter S. (1938). Socioeconomic status and intelligence: A critical survey. Psychological Bulletin.

[B206-jintelligence-11-00141] Neisser Ulric, Boodoo Gwyneth, Bouchard Thomas J., Boykin A. Wade, Brody Nathan, Ceci Stephen J., Halpern Diane F., Loehlin John C., Perloff Robert, Sternberg Robert J. (1996). Intelligence: Knowns and unknowns. American Psychologist.

[B207-jintelligence-11-00141] Neumeister Kristie L. Spiers, Adams Cheryll M., Pierce Rebecca L., Cassady Jerrell C., Dixon Felicia A. (2007). Fourth-grade teachers’ perceptions of giftedness: Implications for identifying and serving diverse gifted students. Journal for the Education of the Gifted.

[B208-jintelligence-11-00141] Norman Antony D., Ramsay Shula G., Martray Carl R., Roberts Julia L. (1999). Relationship between levels of giftedness and psychosocial adjustment. Roeper Review.

[B209-jintelligence-11-00141] Ogurlu Uzeyir (2020). Are Gifted Students Perfectionistic? A Meta-Analysis. Journal for the Education of the Gifted.

[B210-jintelligence-11-00141] Olszewski-Kubilius Paula, Subotnik Rena F., Worrell Frank C. (2016). The role of domains in the conceptualization of talent. Giftedness and Talent in the 21st Century.

[B211-jintelligence-11-00141] Orth Ulrich, Robins Richard W. (2022). Is high self-esteem beneficial? Revisiting a classic question. American Psychologist.

[B212-jintelligence-11-00141] Osmanağaoğlu Nihan, Creswell Cathy, Dodd Helen F. (2018). Intolerance of Uncertainty, anxiety, and worry in children and adolescents: A meta-analysis. Journal of Affective Disorders.

[B213-jintelligence-11-00141] Papadopoulos Dimitrios (2021). Examining the relationships among cognitive ability, domain-specific self-concept, and behavioral self-esteem of gifted children aged 5–6 years: A cross-sectional Study. Behavioral Sciences.

[B214-jintelligence-11-00141] Parker Wayne D. (2000). Healthy perfectionism in the gifted. Journal of Secondary Gifted Education.

[B215-jintelligence-11-00141] Parker Wayne D., Portesová Sarka, Stumpf Heinrich (2001). Perfectionism in mathematically gifted and typical Czech students. Journal for the Education of the Gifted.

[B216-jintelligence-11-00141] Peairs Krsiten F., Putallaz Martha, Costanzo Philip R. (2019). From A (Aggression) to V (Victimization): Peer Status and Adjustment Among Academically Gifted Students in Early Adolescence. Gifted Child Quarterly.

[B217-jintelligence-11-00141] Pearson Marie, Beer John (1990). Self-consciousness, self-esteem and depression of gifted school children. Psychological Reports.

[B218-jintelligence-11-00141] Pek Jolynn, Flora David B. (2018). Reporting effect sizes in original psychological research: A discussion and tutorial. Psychological Methods.

[B219-jintelligence-11-00141] Perera Harsha N., Di Giacomo Michelle (2013). The relationship of trait emotional intelligence with academic performance: A meta-analytic review. Learning and Individual Differences.

[B220-jintelligence-11-00141] Perpiñà Martí Georgina, Sidera Francesc, Morera Fernando Senar, Sellabona Elisabet Serrat (2023). Executive functions are important for academic achievement, but emotional intelligence too. Scandinavian Journal of Psychology.

[B221-jintelligence-11-00141] Peyre Hugo, Ramus Franck, Melchior Maria, Forhan Anne, Heude Barbara, Gauvrit Nicolas (2016). Emotional, behavioral and social difficulties among high-IQ children during the preschool period: Results of the EDEN mother–child cohort. Personality and Individual Differences.

[B222-jintelligence-11-00141] Pérez Josué, Aperribai Leire, Cortabarría Lorea, Borges Africa (2020). Examining the most and least changeable elements of the social representation of giftedness. Sustainability.

[B223-jintelligence-11-00141] Piechowski Michael M., Colangelo Nicholas (1984). Developmental potential of the gifted. Gifted Child Quarterly.

[B224-jintelligence-11-00141] Piirto Jane, Fraas John (2012). A mixed-methods comparison of vocational and identified-gifted high school students on the Overexcitability Questionnaire. Journal for the Education of the Gifted.

[B225-jintelligence-11-00141] Pontes de France-Freitas Maria Luiza, Prette Almir Del, Del Prette Zilda A. P. (2019). Bem estar subjetivo: Um estudo comparativo entre crianças dotadas e talentosas e crianças não dotadas. Boletim-Academia Paulista de Psicologia.

[B226-jintelligence-11-00141] Pottebaum Sheila M., Keith Timothy Z., Ehly Stewart W. (1986). Is there a causal relation between self-concept and academic achievement?. The Journal of Educational Research.

[B227-jintelligence-11-00141] Preckel Franzis, Rach Hannah, Scherrer Vsevolod (2016). Self-concept changes in multiple self-concept domains of gifted students participating in a summer residential school. Gifted and Talented International.

[B228-jintelligence-11-00141] Pufal-Struzik Irena (1999). Self-actualization and other personality dimensions as predictors of mental health of intellectually gifted students. Roeper Review.

[B229-jintelligence-11-00141] Pullmann Helle, Allik Jüri (2008). Relations of academic and general self-esteem to school achievement. Personality and Individual Differences.

[B230-jintelligence-11-00141] Rabner Jonathan, Mian Nicholas D., Langer David A., Comer Jonathan S., Pincus Donna (2016). The Relationship Between Worry and Dimensions of Anxiety Symptoms in Children and Adolescents. Behavioural and Cognitive Psychotherapy.

[B231-jintelligence-11-00141] Ree Malcom James, Earles James A. (1992). Intelligence Is the Best Predictor of Job Performance. Current Directions in Psychological Science.

[B232-jintelligence-11-00141] Reis Sally M., McCoach D. Besty (2000). The underachievement of gifted students: What do we know and where do we go?. Gifted Child Quarterly.

[B233-jintelligence-11-00141] Renzulli Joseph S., Sternberg Robert J., Davidson Janet E. (2005). The three-ring conception of giftedness. Conceptions of Giftedness.

[B234-jintelligence-11-00141] Reser Kristen M. (2016). Perfectionism and Anxiety: Is There a Difference between High-Ability Students and Their Peers?. Ph.D. Thesis.

[B235-jintelligence-11-00141] Reynolds Cecil R., Bradley Michael (1983). Emotional stability of intellectually superior children versus nongifted peers as estimated by chronic anxiety levels. School Psychology Review.

[B236-jintelligence-11-00141] Richards Jane, Encel Jason, Shute Rosalyn (2003). The emotional and behavioural adjustment of intellectually gifted adolescents: A multi-dimensional, multi-informant approach. High Ability Studies.

[B237-jintelligence-11-00141] Riley Tracy L., Karnes Frances A. (1994). Intellectually gifted elementary students’ perceptions of leadership. Perceptual and Motor Skills.

[B238-jintelligence-11-00141] Rinn Anne N., Mendaglio Sal, Rudasill Kathleen Moritz, McQueen Kand S. (2010). Examining the relationship between the overexcitabilities and self-concepts of gifted adolescents via multivariate cluster analysis. Gifted Child Quarterly.

[B239-jintelligence-11-00141] Roberts Shawn M., Lovett Suzanne B. (1994). Examining the “F” in Gifted: Academically Gifted Adolescents’ Physiological and Affective Responses to Scholastic Failure. Journal for the Education of the Gifted.

[B240-jintelligence-11-00141] Robinson Nancy M., Pfeiffer Steven I. (2008). The social world of gifted children and youth. Handbook of Giftedness in Children: Psychoeducational Theory, Research, and Best Practices.

[B241-jintelligence-11-00141] Rodgers Kelly A. (2008). Racial identity, centrality and giftedness: An expectancy-value application of motivation in gifted African American students. Roeper Review.

[B242-jintelligence-11-00141] Rodríguez Naveiras Elena, Borges Emilio Verche, Lastiri Pablo Hernández, López Rubens Montero, Rosal M. África Borges del (2019). Differences in working memory between gifted or talented students and community samples: A meta-analysis. Psicothema.

[B243-jintelligence-11-00141] Roeper Annemarie, Silverman Linda K. (2009). Giftedness and moral promise. Morality, Ethics, and Gifted Minds.

[B244-jintelligence-11-00141] Rosenberg Morris, Schooler Carmi, Schoenbach Carrie (1989). Self-esteem and adolescent problems: Modeling reciprocal effects. American Sociological Review.

[B245-jintelligence-11-00141] Rosenthal Robert (2010). Pygmalion effect. The Corsini Encyclopedia of Psychology.

[B246-jintelligence-11-00141] Roskam Isabelle, Nils Frederic (2007). Predicting intra-individual academic achievement trajectories of adolescents nested in class environment: Influence of motivation, implicit theory of intelligence, self-esteem and parenting. Psychologica Belgica.

[B247-jintelligence-11-00141] Ross Catherine E., Broh Beckett A. (2000). The roles of self-esteem and the sense of personal control in the academic achievement process. Sociology of Education.

[B248-jintelligence-11-00141] Roznowski Mary, Hong Sehee, Reith Janet (2000). A further look at youth intellectual giftedness and its correlates: Values, interests, performance, and behavior. Intelligence.

[B249-jintelligence-11-00141] Ryoo Ji Hoon, Wang Cixin, Swearer Susan M., Park Sunhee (2017). Investigation of Transitions in Bullying/Victimization Statuses of Gifted and General Education Students. Exceptional Children.

[B250-jintelligence-11-00141] Ryum Truls, Kennair Leif Edward, Hjemdal Odin, Hagen Roger, Halvorsen Joar Øveraas, Solem Stian (2017). Worry and Metacognitions as Predictors of Anxiety Symptoms: A Prospective Study. Frontiers in Psychology.

[B251-jintelligence-11-00141] Salovey Peter, Mayer John D. (1990). Emotional intelligence. Imagination, Cognition and Personality.

[B252-jintelligence-11-00141] Sanders Cheryl E., Lubinski David, Benbow Camilla P. (1995). Does the Defining Issues Test measure psychological phenomena distinct from verbal ability?. Journal of Personality and Social Psychology.

[B253-jintelligence-11-00141] Sarouphim Ketty M. (2011). Gifted and non-gifted Lebanese adolescents: Gender differences in self-concept, self-esteem and depression. International Education.

[B254-jintelligence-11-00141] Sánchez-Álvarez Nicolás, Martos Maria Pilar Berrios, Extremera Natalio (2020). A Meta-Analysis of the Relationship Between Emotional Intelligence and Academic Performance in Secondary Education: A Multi-Stream Comparison. Frontiers in Psychology.

[B255-jintelligence-11-00141] Schack Gina D., Starko Alane J. (1990). Identification of gifted students: An analysis of criteria preferred by preservice teachers, classroom teachers, and teachers of the gifted. Journal for the Education of the Gifted.

[B256-jintelligence-11-00141] Schmidt Jennifer A., Padilla Brenda (2003). Self-esteem and family challenge: An investigation of their effects on achievement. Journal of Youth and Adolescence.

[B257-jintelligence-11-00141] Scholwinski Ed, Reynolds Cecile R. (1985). Dimensions of Anxiety Among High IQ Children. Gifted Child Quarterly.

[B258-jintelligence-11-00141] Schuler Patricia A. (2000). Perfectionism and gifted adolescents. Journal of Secondary Gifted Education.

[B259-jintelligence-11-00141] Schulte Melanie J., Ree Malcolm James, Carretta Thomas R. (2004). Emotional intelligence: Not much more than g and personality. Personality and Individual Differences.

[B260-jintelligence-11-00141] Schwean Vicki L., Saklofske Donald H., Widdifield-Konkin Leslie, Parker James D. A., Kloosterman Patricia (2006). Emotional intelligence and gifted children. E-Journal of Applied Psychology.

[B261-jintelligence-11-00141] Sekowski Andrzej (1995). Self-esteem and achievements of gifted students. Gifted Education International.

[B262-jintelligence-11-00141] Shade Rick (1991). Verbal Humor in Gifted Students and Students in the General Population: A Comparison of Spontaneous Mirth and Comprehension. Journal for the Education of the Gifted.

[B263-jintelligence-11-00141] Sharifi Hamzeh, Sharifi Mandana (2014). Comparing emotional intelligence and humor in gifted and nongifted students. Indian Journal of Scientific Research.

[B264-jintelligence-11-00141] Shaunessy Elizabeth, Suldo Shannon M., Hardesty Robin B., Shaffer Emily J. (2006). School Functioning and Psychological Well-Being of International Baccalaureate and General Education Students A Preliminary Examination. Journal of Secondary Gifted Education.

[B265-jintelligence-11-00141] Shechtman Zipora, Silektor Anat (2012). Social Competencies and Difficulties of Gifted Children Compared to Nongifted Peers. Roeper Review.

[B266-jintelligence-11-00141] Siegle Del, Powell Teri (2004). Exploring Teacher Biases When Nominating Students for Gifted Programs. Gifted Child Quarterly.

[B267-jintelligence-11-00141] Simon William E., Simon Marilyn G. (1975). Self-esteem, intelligence and standardized academic achievement. Psychology in the Schools.

[B268-jintelligence-11-00141] Simmons Carolyn H., Zumpf Connie (1986). The gifted child: Perceived competence, prosocial moral reasoning, and charitable donations. The Journal of Genetic Psychology.

[B269-jintelligence-11-00141] Simoes Loureiro Isabelle, Lowenthal Francis, Lefebvre Laurent, Vaivre-Douret L.aurence (2010). Étude des caractéristiques psychologiques et psychobiologiques des enfants à haut potentiel. Enfance.

[B270-jintelligence-11-00141] Sirois Fuschia M., Molnar Danielle S., Hirsch Jameson K. (2017). A Meta-analytic and Conceptual Update on the Associations Between Procrastination and Multidimensional Perfectionism. European Journal of Personality.

[B271-jintelligence-11-00141] Siu Angela F. Y. (2010). Comparing overexcitabilities of gifted and non-gifted school children in Hong Kong: Does culture make a difference?. Asia Pacific Journal of Education.

[B272-jintelligence-11-00141] Skaalvik Einar M., Hagtvet Knut A. (1990). Academic achievement and self-concept: An analysis of causal predominance in a developmental perspective. Journal of Personality and Social Psychology.

[B273-jintelligence-11-00141] Steiner Hillary Hettinger, Carr Martha (2003). Cognitive Development in Gifted Children: Toward a More Precise Understanding of Emerging Differences in Intelligence. Educational Psychology Review.

[B274-jintelligence-11-00141] Sternberg Robert J., Davidson Janet E. (2005). Conceptions of Giftedness.

[B275-jintelligence-11-00141] Stornelli Deborah, Flett Gordon L., Hewitt Paul L. (2009). Perfectionism, achievement, and affect in children: A comparison of students from gifted, arts, and regular programs. Canadian Journal of School Psychology.

[B276-jintelligence-11-00141] Strenze Tarmo (2007). Intelligence and socioeconomic success: A meta-analytic review of longitudinal research. Intelligence.

[B277-jintelligence-11-00141] Stricker Johannes, Preckel Franzis (2022). Global self-esteem differentially predicts multidimensional perfectionism in early adolescents two years later. The Journal of Early Adolescence.

[B278-jintelligence-11-00141] Stricker Johannes, Buecker Susanne, Schneider Michael, Preckel Franzis (2019). Intellectual Giftedness and Multidimensional Perfectionism: A Meta-Analytic Review. Educational Psychology Review.

[B279-jintelligence-11-00141] Subotnik Rena F., Olszewski-Kubilius Paula, Worrell Frank C. (2011). Rethinking giftedness and gifted education: A proposed direction forward based on psychological science. Psychological Science in the Public Interest.

[B280-jintelligence-11-00141] Swiatek Mary Ann (1995). An Empirical Investigation of the Social Coping Strategies Used by Gifted Adolescents. Gifted Child Quarterly.

[B281-jintelligence-11-00141] Swiatek Mary Ann (2001). Social Coping Among Gifted High School Students and its Relationship to Self-Concept. Journal of Youth and Adolescence.

[B282-jintelligence-11-00141] Tan-Willman Conchita, Gutteridge D. (1981). Creative thinking and moral reasoning of academically gifted secondary school adolescents. Gifted Child Quarterly.

[B283-jintelligence-11-00141] Tavani Jean Louis, Zenasni Franck, Pereira-Fradin Maria (2009). Social representation of gifted children: A preliminary study in France. Gifted and Talented International.

[B284-jintelligence-11-00141] Tetzner Julia, Becker Michael, Maaz Kai (2017). Development in multiple areas of life in adolescence: Interrelations between academic achievement, perceived peer acceptance, and self-esteem. International Journal of Behavioral Development.

[B285-jintelligence-11-00141] Thomas Jerald A. (2008). Reviving Perry: An analysis of epistemological change by gender and ethnicity among gifted high school students. Gifted Child Quarterly.

[B286-jintelligence-11-00141] Thorson James A., Powell F. C. (1993). Development and validation of a multidimensional sense of humor scale. Journal of Clinical Psychology.

[B287-jintelligence-11-00141] Tidwell Romeria (1980). A psycho-educational profile of 1593 gifted high school students. Gifted Child Quarterly.

[B288-jintelligence-11-00141] Tieso Carol L. (2007). Patterns of overexcitabilities in identified gifted students and their parents: A hierarchical model. Gifted Child Quarterly.

[B289-jintelligence-11-00141] Tirri Kirsi (2010). Combining excellence and ethics: Implications for moral education for the gifted. Roeper Review.

[B290-jintelligence-11-00141] Tirri Kirsi, Nokelainen Petri (2007). Comparison of academically average and gifted students’ self-rated ethical sensitivity. Educational Research and Evaluation.

[B291-jintelligence-11-00141] Tirri Kirsi, Pehkonen Leila (2002). The moral reasoning and scientific argumentation of gifted adolescents. Journal of Secondary Gifted Education.

[B292-jintelligence-11-00141] Topçu Sevgi, Leana-Taşcılar Marilena Z. (2018). The role of motivation and self-esteem in the academic achievement of Turkish gifted students. Gifted Education International.

[B293-jintelligence-11-00141] Trowbridge Norma (1974). Self-concept and IQ in elementary school children. California Journal of Educational Research.

[B294-jintelligence-11-00141] Van Tassel-Baska Joyce, Olszewski-Kubilius Paula, Kulieke Marilyn (1994). A study of self-concept and social support in advantaged and disadvantaged seventh and eighth grade gifted students. Roeper Review.

[B295-jintelligence-11-00141] Vialle Wilma, Heaven Patrick C. L., Ciarrochi Joseph (2005). The relationship between self-esteem and academic achievement in high ability students: Evidence from the Wollongong Youth Study. Australasian Journal of Gifted Education.

[B296-jintelligence-11-00141] Vialle Wilma, Heaven Patrick C. L., Ciarrochi Joseph (2007). On Being Gifted, but Sad and Misunderstood: Social, emotional, and academic outcomes of gifted students in the Wollongong Youth Study. Educational Research and Evaluation.

[B297-jintelligence-11-00141] Vrticka Pascal, Black Jessica M., Neely Michelle, Shelly Elizabeth Walter, Reiss Allan L. (2013). Humor processing in children: Influence of temperament, age and IQ. Neuropsychologia.

[B298-jintelligence-11-00141] Vuyk M. Alexandra (2010). Relating Perfectionism, Overexcitabilities and Depressive Symptoms among Gifted Adolescents in Paraguay. Ph.D. Thesis.

[B299-jintelligence-11-00141] Vuyk M. Alexandra, Kerr Barbara A., Krieshok Thomas S. (2016). From overexcitabilities to openness: Informing gifted education with psychological science. Gifted and Talented International.

[B300-jintelligence-11-00141] Warne Russel T. (2011). A reliability generalization of the Overexcitability Questionnaire–Two. Journal of Advanced Academics.

[B301-jintelligence-11-00141] Waterhouse Lynn (2006). Multiple intelligences, the Mozart effect, and emotional intelligence: A critical review. Educational Psychologist.

[B302-jintelligence-11-00141] Watkins Marley W., Lei Pui-Wa, Canivez Gary L. (2007). Psychometric intelligence and achievement: A cross-lagged panel analysis. Intelligence.

[B303-jintelligence-11-00141] Weyns Tessa, Colpin Hilde, Verschueren Karine (2021). The role of school-based relationships for school well-being: How different are high-and average-ability students?. British Journal of Educational Psychology.

[B304-jintelligence-11-00141] Wigfield Allan, Eccles Jacquelynne S. (1994). Children’s competence beliefs, achievement values, and general self-esteem: Change across elementary and middle school. The Journal of Early Adolescence.

[B305-jintelligence-11-00141] Willinger Ulrike, Hergovich Andreas, Schmoeger Michaela, Deckert Matthias, Stoettner Susanne, Bunda Iris, Witting Andrea, Seidler Melanie, Moser Reinhilde, Kacena Stefanie (2017). Cognitive and emotional demands of black humour processing: The role of intelligence, aggressiveness and mood. Cognitive Processing.

[B306-jintelligence-11-00141] Wills Thomas Ashby (1994). Self-esteem and perceived control in adolescent substance use: Comparative tests in concurrent and prospective analyses. Psychology of Addictive Behaviors.

[B307-jintelligence-11-00141] Winkler Daniel, Voight Adam (2016). Giftedness and overexcitability: Investigating the relationship using meta-analysis. Gifted Child Quarterly.

[B308-jintelligence-11-00141] Winne Philip H., Woodlands Margaret J., Wong Bernice Y. L. (1982). Comparability of self-concept among learning disabled, normal, and gifted students. Journal of Learning Disabilities.

[B309-jintelligence-11-00141] Winner Ellen (2000). The origins and ends of giftedness. American Psychologist.

[B310-jintelligence-11-00141] Wirthwein Linda, Becker Carolin V., Loehr Eva Maria, Rost Detlef H. (2011). Overexcitabilities in gifted and non-gifted adults: Does sex matter?. High Ability Studies.

[B311-jintelligence-11-00141] Wirthwein Linda, Bergold Sebastian, Preckel Franzis, Steinmayr Ricarda (2019). Personality and school functioning of intellectually gifted and nongifted adolescents: Self-perceptions and parents’ assessments. Learning and Individual Differences.

[B312-jintelligence-11-00141] Woitaszewski Scott A., Aalsma Matthew C. (2004). The contribution of emotional intelligence to the social and academic success of gifted adolescents as measured by the multifactor emotional intelligence scale-adolescent version. Roeper Review.

[B313-jintelligence-11-00141] Wood Vanessa R., Laycraft Krystyna C. (2020). How can we better understand, identify, and support highly gifted and profoundly gifted students? A literature review of the psychological development of highly-profoundly gifted individuals and overexcitabilities. Annals of Cognitive Science.

[B314-jintelligence-11-00141] Yakmaci-Guzel Buket, Akarsu Fusun (2006). Comparing overexcitabilities of gifted and non-gifted 10th grade students in Turkey. High Ability Studies.

[B315-jintelligence-11-00141] Yang Qian, Tian Lili, Huebner E. Scott, Zhu Xinxin (2019). Relations among academic achievement, self-esteem, and subjective well-being in school among elementary school students: A longitudinal mediation model. School Psychology.

[B316-jintelligence-11-00141] Yi Soohyun, Gentry Marica (2021). Academic perfectionism of high-ability and high-achieving students in mathematics and science: Differential relations by identification criteria of giftedness. Roeper Review.

[B317-jintelligence-11-00141] Yong Fung L., McIntyre John D. (1991). Comparison of Self-Concepts of Students Identified as Gifted and Regular Students. Perceptual and Motor Skills.

[B318-jintelligence-11-00141] Yoon So Yoon, Gentry Marcia (2009). Racial and ethnic representation in gifted programs: Current status of and implications for gifted Asian American students. Gifted Child Quarterly.

[B319-jintelligence-11-00141] Zeidner Moshe (2017). Tentative guidelines for the development of an ability-based emotional intelligence intervention program for gifted students. High Ability Studies.

[B320-jintelligence-11-00141] Zeidner Moshe (2021). “Don’t worry—Be happy”: The sad state of happiness research in gifted students. High Ability Studies.

[B321-jintelligence-11-00141] Zeidner Moshe, Matthews Gerald (2017). Emotional intelligence in gifted students. Gifted Education International.

[B322-jintelligence-11-00141] Zeidner Moshe, Schleyer Esther Jane (1999). Test Anxiety in intellectually gifted school students. Anxiety, Stress and Coping.

[B323-jintelligence-11-00141] Zeidner Moshe, Shani-Zinovich Inbal (2011). Do academically gifted and nongifted students differ on the Big-Five and adaptive status? Some recent data and conclusions. Personality and Individual Differences.

[B324-jintelligence-11-00141] Zeidner Moshe, Shani-Zinovich Inbal (2015). A comparison of multiple facets of self-concept in gifted vs. non-identified Israeli students. High Ability Studies.

[B325-jintelligence-11-00141] Zeidner Moshe, Shani-Zinovich Inbal, Matthews Gerald, Roberts Richard D. (2005). Assessing emotional intelligence in gifted and non-gifted high school students: Outcomes depend on the measure. Intelligence.

[B326-jintelligence-11-00141] Ziv Avner (1990). Humor and Giftedness. Gifted International.

[B327-jintelligence-11-00141] Zschaler Andrea (2019). “Help! I Am Gifted”: The Development of Self-Concept (Self-Theory) and Self-Esteem Amongst Gifted Students in English Secondary Schools and the Influence of School Culture. Ph.D. Thesis.

[B328-jintelligence-11-00141] Zuffianò Antonio, Alessandri Guido, Gerbino Maria, Kanacri Bernadette Paula Luengo, Giunta Laura Di, Milioni Michela, Caprara Gian Vittorio (2013). Academic achievement: The unique contribution of self-efficacy beliefs in self-regulated learning beyond intelligence, personality traits, and self-esteem. Learning and Individual Differences.

