# Supplementary material for: Non-Cognitive Specificities of Intellectually Gifted Children and Adolescents: A Systematic Review of the Literature
Source: J Intell. 2023 Jul 15;11(7):141. doi: 10.3390/jintelligence11070141 (PMC10382067; doi:10.3390/jintelligence11070141)
Supplement: Supplementary file 1 [file jintelligence-11-00141-s001.zip › jintelligence-2398675-supplementary.pdf]

Table S1

Summary table of methodological aspects and effect sizes from articles on general anxiety.

Articles	Age Range	Sampling Methodology	Sample Size Gifted Group	Sample Size Non-Gifted Group	Effect Size and % of Variance Explained	Methodological Remarks
Francis et al., 2018	9 - 15 years	Intelligence Test<br>- Verbal IQ<br>- Performance IQ<br>- Fulll-Scale IQ<br>Gr. 1: IQ ≥ 130<br>Gr. 2: 115 - 129<br>Gr. 3: IQ ≤ 114	Participants: $N = 312$<br>- Verbal IQ<br>Gr. 1: n =67<br>Gr. 2: n = 127<br>Gr. 3: n = 118<br>- Performance IQ<br>Gr. 1: n = 41<br>Gr. 2: n = 106<br>Gr. 3: n = 165<br>- Full-Scale IQ<br>Gr. 1: n =63<br>Gr. 2: n = 137<br>Gr. 3: n = 112		Verbal IQ / Intolerance of Uncertainty<br>$r = -.17, p < 0.01$<br>3.89% var. explained	3 Intelligence Indicators:<br>- Verbal IQ<br>- Performance IQ<br>- Full Scale IQ
Peyre et al., 2016	5 – 6 years	EDEN Mother-Child Cohort<br>Gr. 1: 70 > IQ<br>Gr. 2: 70 < IQ ≤ 120<br>Gr. 3: IQ > 130	Gr. 3: n = 23	Gr. 1: n =19<br>Gr. 2: n = 1058	Comparison between Group 2 and 3 / Emotional Symptoms<br>$d = .43, p = .045$<br>4.41 % var. explained	—

Articles	Age Range	Sampling Methodology	Sample Size Gifted Group	Sample Size Non-Gifted Group	Effect Size and % of Variance Explained	Methodological Remarks
Francis et al., 2015<br>(M)	5 - 19 years	18 Studies,<br>6 covering Anxiety<br>- IQ > 120 (90 ≤ IQ ≤ 110 in the Non-Gifted Group)<br>OR<br>- IQ used as a continuous variable from 90 to 125+	—	—	No Overall Effect Available	—
Gauvrit, 2014<br>(M)	—	13 Studies<br>Mixed Designs<br>- IQ<br>- Academic Achievement<br>- Unknown Methodology	—	—	—	—
Guenolé et al., 2013	8 - 12 years	Clinical Population<br>IQ ≥ 130	*n* = 106	Normative data	—<br>n.s	No control group.

Articles	Age Range	Sampling Methodology	Sample Size Gifted Group	Sample Size Non-Gifted Group	Effect Size and % of Variance Explained	Methodological Remarks
Zeidner & Shani-Zinovich, 2011	Grades 10-12	Gifted Program<br>- IQ<br>- Academic Achievement	*n* = 374	*n* = 428	Group / Anxiety Scale<br>η2 partial = .02<br>2 % var. explained	—
Martin et al., 2010<br><br>(M)	5 - 18 years	4 Studies devoted to Anxiety<br>No Criteria regarding Giftedness	—	—	Group / Anxiety<br>Overall *d* = - .72<br>11.49 % var. explained	—
Cross et al., 2008	Grade 11-12	Gifted Program	*n* = 567	Normative data	Group / Anxiety Subscale<br>*d* = .40<br>3.84 % var. explained	No control group.
Cernova, 2005	11 - 15 years	IQ > 129	*n* = 62	*n* = 104	—	Unspecified methodology.
Pufal-Struzik, 1999	16 – 17 years	- Teacher Nomination<br>- Academic Achievement	*n* = 65	*n* = 75	—<br>n.s	—

Articles	Age Range	Sampling Methodology	Sample Size Gifted Group	Sample Size Non-Gifted Group	Effect Size and % of Variance Explained	Methodological Remarks
Czeschlik & Rost, 1994	—	$121 \leq IQ \leq 134$	$n = 50$	$n = 50$	—<br>n.s	Mixed design with three data sources (children, parents, and teachers) on the children's personality and socio-emotional behavior.
Beer, 1991	- Junior High School Students<br>- High School Students	Gifted Programs:<br>- Intelligence Scores at the 97th percentile<br>- Academic Scores at the 95th percentile	$n = 27$	Normative data	—	No control group.
Scholwinski & Reynolds, 1985	7 - 18 years	Gifted Program<br>$IQ \geq 130$	$n = 584$	$n = 4\,923$	—	—
Reynolds & Bradley, 1983	Grades 1 - 12	Intelligence Test<br>$IQ \geq 129$	$n = 465$	$n = 329$	—	—
Milgram & Milgram, 1976	Grades 4 - 8	After School Classes<br>$IQ \geq 135$	$n = 182$	$n = 310$	Gifted scores more favorable than control scores, $p < .01$	Unspecified methodology.

Articles	Age Range	Sampling Methodology	Sample Size Gifted Group	Sample Size Non-Gifted Group	Effect Size and % of Variance Explained	Methodological Remarks
Feldhusen & Klausmeier, 1962	—	Gr. 1: IQ 56 to 81<br>Gr.2: IQ 90 to 110<br>Gr.3: IQ 120 to 146	*n* = 40 per group	*n* = 40 per group	IQ / Anxiety<br>- Gr. 1<br>*r* = - .28, n.s<br>7.84 % var. explained<br>- Gr. 2<br>*r* = - .35, *p* = .05<br>12.25 % var. explained<br>- Gr. 3<br>*r* = - .07, n.s<br>0.49 % var. explained	—

Note. __ indicates that the information is not available in the article; (M) indicates that the study is a meta-analysis.

Table S2

Summary table of methodological aspects and effect sizes from articles on test anxiety.

Articles	Age Range	Sampling Methodology	Sample Size Gifted Group	Sample Size Non-Gifted Group	Effect Size and % of Variance Explained	Methodological Remarks
Zeidner & Schleyer, 1999 — Study 1	Grades 4 - 9	Gifted Program<br>- IQ<br>- Academic Achievement	*n* = 772	*n* = 716	Non Gifted scored Higher by the order of .28 Standard Deviations	More information regarding educational style is available in Study 2.
Ackerman & Heggestad, 1997<br><br>(M)	10 years<br>and Above	Data Available for each Indicator<br><br>*g*<br>21 studies,<br>*N* = 3 027<br><br>*Gc*<br>21 studies,<br>*N* = 4 714<br><br>*Gf*<br>4 studies,<br>*N* = 784	—	—	- g / Test Anxiety<br>ρ = - .33, $p < .05$<br>10.89 % var. explained<br>- Gc / Test Anxiety<br>ρ = - .24, $p < .05$<br>5.76 % var. explained<br>- Gf / Test Anxiety<br>ρ = - .25, $p < .05$<br>6.25 % var. explained	- Data are available for each cognitive ability, in addition to general intelligence, Gf and Gc scores.<br><br>- Studies only assessing ability as creativity or psychomotor abilities were excluded.
Beer, 1991	- Junior High School Students<br>- High School Students	Gifted Programs:<br>- Intelligence Scores at the 97th percentile<br>- Academic Scores at the 95th percentile	*n* = 27	Normative data	—	No control group.

Articles	Age Range	Sampling Methodology	Sample Size Gifted Group		Sample Size Non-Gifted Group	Effect Size and % of Variance Explained
Hembree, 1988<br><br>(M)	—	66 Studies<br>Giftedness based on IQ	$N = 9430$		- Grades 1 and 2<br>$r = -.10, p < .01$<br>1 % var. explained<br><br>- Grades 3 to postsecondary<br>$r = -.23, p < .01$<br>5.29 % var. explained	Data available for IQ, aptitude, achievement (562 studies included overall).
Milgram & Milgram, 1976	Grades 4 - 8	After School Classes<br>IQ ≥ 135	n =182	n = 310	Gifted scores more favorable than control scores, $p < .001$	Unspecified methodology.

Note. __ indicates that the information is not available in the article; (M) indicates that the study is a meta-analysis.

Table S3

Summary table of methodological aspects and effect sizes from articles on perfectionism.

Articles	Age Range	Sampling Methodology	Sample Size Gifted Group	Sample Size Non-Gifted Group	Effect Size and % of Variance Explained	Methodological Remarks
Yi & Gentry, 2021	Grades 10-11	Gifted program based on aptitude tests and creative problem-solving tests in math and science	*n* = 180	*n* = 263	Academic perfectionism / Giftedness<br>NS, especially if the intellectual ability was emphasized for the gifted identification	—
Ogurlu, 2020<br><br>(M)	—	14 studies<br>Being Identified as Intellectually Gifted	—	—	- Group/Perfectionistic Concerns<br>*g* = - .13, n.s<br>0.42 % var. explained<br><br>- Group/Perfectionistic Strivings<br>*g* = .19, n.s<br>0.90 % var. explained	All the studies include control groups.
Stricker et al., 2019<br><br>(M)	—	10 studies<br>Being Identified as Intellectually Gifted	*n* = 1 902	*n* = 2 438	- Group/Perfectionistic Concerns<br>*g* = - .12, n.s<br>0.36 % var.explained<br><br>- Group/Perfectionistic Strivings<br>*g* = .33, $p < .05$<br>2.66 % var. explained	All the studies include control groups.

Articles	Age Range	Sampling Methodology	Sample Size Gifted Group	Sample Size Non-Gifted Group	Effect Size and % of Variance Explained	Methodological Remarks
Guignard et al., 2012	Grades 5 and 6	Intelligence Test<br>IQ > 130 or equivalent	*n* = 61	*n* = 71	- Total Scores on CAPS / RCMAS<br>$r = .35, p < .01$<br>12.25 % var. explained<br><br>- Self-Oriented Perfectionism / RCMAS Total<br>$r = .25, p < .01$<br>6.25 % var. explained<br><br>- Socially Prescribed Perfectionism / RCMAS<br>$r = .36, p < .01$<br>12.96 % var. explained	- One of the schools in the sample relies on text comprehension and Cattel's Culture Fair Test.<br><br>- Participants are matched by grade level and age.
Stornelli et al., 2009	Grade 4 or 7		Gifted:<br>*n* = 86<br><br>(Fine art programs, *n* = 33)	*n* = 162	Group / Self-oriented perfectionism<br>NS<br><br>Perfectionnism / Reading<br>Gifted: $r = .04$, ns<br>Control: $r = - .09$, ns<br><br>Perfectionnism / Mathematics<br>Gifted: $r = .09$, ns<br>Control: $r = .26, p < .05$	No information regarding giftedness identification or integration into a special program.
Roberts & Lovett, 1994	Grades 7 and 8	- Pull-out Program for Gifted<br>- Academic Achievement	Gifted:<br>*n* = 20<br><br>Achievers:<br>*n* = 20	*n* = 20	—	Achievers are not identified as gifted or enrolled in special programs

Note. __ indicates that the information is not available in the article; (M) indicates that the study is a meta-analysis.

Table S4

Summary table of methodological aspects and effect sizes from articles on depression.

Articles	Age Range	Sampling Methodology	Sample Size Gifted Group	Sample Size Non-Gifted Group	Effect Size and % of Variance Explained	Methodological Remarks
Francis et al., 2015<br>(M)	5 - 19 years	18 Studies Overall, 3 covering Depression<br>- IQ > 120 (90 ≤ IQ ≤ 110 in the Non-Gifted Group)<br>OR<br>- IQ used as a continuous variable from 90 to 125+	—	__	No Overall Effect Available	__
Martin et al., 2010<br>(M)	5 - 18 years	9 Studies Overall, 6 covering Depression<br>No criteria regarding Giftedness	—	—	Group / Depression<br>Overall *d* = - .17, n.s<br>0.72 % var. explained	__
Cross et al., 2008	Grades 11 and 12	Gifted Program	*n* = 567	Normative data	Group / Depression Subscale<br>*d* = .49<br>5.66 % var. explained	No control group.
Benony, 2007	8 - 13 years	Gifted Program<br>IQ ≥ 130	*n* = 23	*n* = 23	—	__

Note. __ indicates that the information is not available in the article; (M) indicates that the study is a meta-analysis.

Table S5

Summary table of methodological aspects and effect sizes from articles on suicidal ideation.

Articles	Age Range	Sampling Methodology	Sample Size Gifted Group	Sample Size Non-Gifted Group	Effect Size and % of Variance Explained	Methodological Remarks
Cross et al., 2006	High School Students	Gifted Program	*n* = 152	Normative data	—<br>n.s	No control group.
Metha & McWhirter, 1997	Grades 7 and 8	Gifted Program	*n* = 34	*n* = 38	Group / Suicide Ideation<br>*d* = .20, n.s<br>1 % var. explained	The Gifted Program integrates high-achieving, creative and talented students.
Baker, 1995	Junior High School to 11th Grade	Exceptionally Gifted:<br>- Gifted Program based on SAT > 900<br>Gifted:<br>- Achievement<br>- Gifted Program based on SAT ≤ 600	Exceptionally Gifted: *n* = 32<br>Gifted: *n* = 58	*n* = 56	—<br>n.s	—

Note. __ indicates that the information is not available in the article; (M) indicates that the study is a meta-analysis.

Table S6

Summary table of methodological aspects and effect sizes from articles on other mood disorders.

Articles	Age Range	Sampling Methodology	Sample Size Gifted Group	Sample Size Non-Gifted Group	Effect Size and % of Variance Explained	Methodological Remarks
Cook et al., 2020	8 months - 11 years	Longitudinal Community Cohort<br><br>IQ ≥ 120	*n* = 192	*n* = 1 015	Group / Clinical Range<br>*d* = .59<br>8 % var. explained<br><br>IQ Score / Mental Health Difficulties Scores<br>*r* = - .14, *p* < .001<br>1.96 % var. explained<br>(Age 5 and 7)<br>To<br>*r* = - .17, *p* < .001<br>2.89 % var. explained<br>(Age 11)<br>1.96 % Median var. explained	Parents are not informed of child's IQ test results, limiting the gifted labeling influence.
MacCabe et al., 2010	—	National School Register<br><br>Academic Achievement (Grade A on National Examination, > 2 Deviations Above the Mean)	*n* = 9 427	*n* = 704 449	Grade / Risk Factor for Bipolar Disorder<br>Adjusted Hazard Ratio<br>3.34 (1.82–6.11)<br>9.92 % * var. explained	—

Articles	Age Range	Sampling Methodology	Sample Size Gifted Group	Sample Size Non-Gifted Group	Effect Size and % of Variance Explained	Methodological Remarks
Koenen et al., 2009	—	Longitudinale Cohort<br>IQ ≥ 115	n = 141	n = 794	Chilhood IQ / Prevalence of Psychiatric Disorders<br>d = .06, n.s<br>0.09 % var. explained<br>To<br>d = 1.28, p < .05<br>29.05 % var. explained<br>2.12 % Median var. explained	—
Cross et al., 2008	Grades 11 - 12	Gifted Program	n = 567	Normative data	Group / Mood Disorders<br>d = .32<br>2.5 % var. explained<br>To<br>d = .76<br>12.6 % var. explained<br>5.77 % Median var. explained	—

Note. __ indicates that the information is not available in the article; (M) indicates that the study is a meta-analysis.
* This score is approximate because it is based on an odds ratio conversion.

Table S7

Summary table of methodological aspects and effect sizes from articles on objective indicators of achievement and quality of life.

Articles	Age Range	Sampling Methodology	Sample Size Gifted Group	Sample Size Non-Gifted Group	Effect Size and % of Variance Explained	Methodological Remarks
Bergold et al., 2020	Grades 9 and 10	IQ > 130	$n = 50$	$n = 50$	- Gifted Group / Math Achievement Test<br>$r = .66$, $p < .001$<br>43.56 % var. explained<br><br>- Gifted Group / Reading Comprehension<br>$r = .23$, n.s<br>5.29 % var. explained<br><br>- Gifted Group / Math Grades<br>$r = .81$, $p < .001$<br>65.61 % var. explained<br><br>- Gifted Group / German Grades<br>$r = .25$, n.s<br>6.25 % var. explained	SES is considered as a covariable.
Demetriou et al., 2020	10 to 16 years old	Cognitive tasks:<br>- quantitative reasoning,<br>- causal reasoning,<br>- spatial reasoning,<br>- social reasoning	N= 408		Cognitive Performance /Academic performance, r between .35 and .42, $p < .001$	—

Articles	Age Range	Sampling Methodology	Sample Size Gifted Group	Sample Size Non-Gifted Group	Effect Size and % of Variance Explained	Methodological Remarks
Li & Shi, 2019	8 to 11 years old	- Gifted program<br>- Stanford-Binet, Weschler<br>- Raven's Standard Progressive Matrices	$n = 80$	$n = 104$	Academic performance:<br>Gifted group > Control group<br>Mathematics, $p < 0.001$<br>Chinese, $p < 0.001$<br>English, $p < 0.001$	—
Wirthwein et al., 2019	Grades 11 and 12	Standardized Intelligence Score > 120<br>(Equivalent of IQ > 130)	$n = 97$	$n = 97$	Gifted Group / School Performance<br>$d = -.63, p < .001$<br>9 % var. explained<br>To<br>$d = -1.02, p < .001$<br>20.62 % var. explained<br>Among Subjects<br>15.65 % Median var. explained	—
Guez et al., 2018	Grades 6 and 9	Longitudinal Study<br>Non-Verbal IQ ≥ 126	$n = 888$	$n = 29\ 601$	IQ group / Academic Examination<br>$d = .97$<br>19 % var. explained	Considering social background and family support.

Articles	Age Range	Sampling Methodology	Sample Size Gifted Group	Sample Size Non-Gifted Group	Effect Size and % of Variance Explained
Eklund et al., 2015	5 - 12 years	Teacher Nomination within Gifted Programs	N = 1 206	Group / Academic Performance<br>- Parents ratings<br>partial η^2 = .19, p = .00<br>19 % var. explained<br>- Teacher ratings<br>partial η^2 = .26, p = .00<br>26 % var. explained	Unspecified methodology regarding groups.
Deary et al., 2008	—	Longitudinal Study	—	Hazard ratio for IQ per SD and mortality of 0.80; 95% CI 5 0.65–0.99, p = .037<br>1.51 % * var. explained	—
Deary et al., 2007	11 years and 16 years	Longitudinal Study	CAT sample<br>n = 74 403<br>National Examination<br>n = 361 335	g / Educational Achievement<br>r = .81<br>65.61 % var. explained	—
Strenze, 2007<br>(M)	Age at testing from 3 to 23 years<br>Age at success from 20 to 78 years	- Academic performance (GPA or rank)<br>- Intelligence tests	Correlation Intelligence / occupation<br>N = 72,290<br>Correlation Intelligence / income<br>N = 58,758	Intelligence / occupation<br>r = .37 (sample size weighted average correlation r = .36)<br>Intelligence / income<br>r = .21 (sample size weighted average correlation r = .16)	—

Articles	Age Range	Sampling Methodology	Sample Size Gifted Group	Sample Size Non-Gifted Group	Effect Size and % of Variance Explained	Methodological Remarks
Neisser et al., 1996	—	—	—	—	- IQ scores / Grades<br>*r* = .50<br>25 % var. explained<br><br>- IQ scores / Years of Education<br>*r* = .55<br>30.25 % var. explained	—
Ree & Earles, 1992	—	Military Cohorts	College Graduate Lieutenants<br>*N* = 5 500<br><br>Airmens<br>*N* = 1 206		*g* / Job Performance<br><br>- College Graduate Lieutenants<br>*r* = .33<br>10.89 % var. explained<br><br>- Airmens<br>*r* = .44<br>19.36 % var. explained	—

Note. __ indicates that the information is not available in the article; (M) indicates that the study is a meta-analysis.
* This score is approximate because it is based on an odds ratio conversion.

Table S8

Summary table of methodological aspects and effect sizes from articles on life satisfaction and subjective well-being (SWB).

Articles	Age Range	Sampling Methodology	Sample Size Gifted Group	Sample Size Non-Gifted Group	Effect Size and % of Variance Explained	Methodological Remarks
Guignard et al., 2021	Grade 6-10	- Labeled as gifted<br>- General intelligence test D48 (Pichot)	*n* = 66	*n* = 426	Labelled gifted and non-labelled:<br>*NS* on school life satisfaction $t(490) = -.17$, $p = .87$<br>School life satisfaction/D48<br>$r = .089$, $p = .049$	Labelled as gifted and D48 failed to reach significance ($chi^2 = 3.82$, $p = .051$)
Bergold et al., 2020	Grade 9-10	Cultural Fair Intelligence Test Scale 2<br>Gifted IQ > 130<br>Average ability<br>85 < IQ < 115	*n* = 50	*n* = 50	Gifted > Control:<br>Mathematics achievement test, p < .001<br>*NS* in reading comprehension and German grade<br>Giftedness /<br>Subjective Well-Being<br>*NS*	—
Zeidner, 2020<br>(M)	Primary School to High School	6 Studies	—	—	Group / SWB<br>Summary effect $g = -.01$, (min $d = -.54$; max $d = -.03$)<br>0.0 % var. explained	All studies included control groups and allowed calculation of effect sizes.
Pontes de França-Freitas et al., 2019	8 - 12 years	Centers for the Development of Potential and Talent	*n* = 269	*n* = 125	—	—

Articles	Age Range	Sampling Methodology	Sample Size Gifted Group	Sample Size Non-Gifted Group	Effect Size and % of Variance Explained	Methodological Remarks
Bücker et al., 2018<br>(M)	—	47 Studies	$N = 38\ 946$		Academic Achievement / Subjective Well-Being<br>Overall $r = .16$,<br>(Coded Effect Sizes ranging from $r = -.47$ to $r = .68$)<br>2.6 % var. explained	—
Cheng & Furnham, 2014	Intelligence at age 11<br>Educational achievement and occupational prestige at age 50	General ability test<br>(40 verbal and 40 non-verbal items)	$N = 5{,}090$		Verbal Ability / Education, $r = .426$, $p < .05$<br>Non-verbal Ability / Education, $r = .402$, $p < .05$<br>Verbal Ability / Occupational prestige, $r = .32$, $p < .05$<br>Non-verbal Ability / Occupational prestige, $r = .305$, $p < .05$	—
Chmiel et al., 2012	End of Primary School, Mostly Grade 6	Longitudinal Study	$N = 738$		Prediction of SWB component / General Cognitive Ability<br>β = .04<br>0.16 % var. explained	—
Huebner & Alderman, 1993	Grades 2 - 9	- Initial Psychological Evaluations<br>OR<br>- 3-year reevaluations	$N = 53$		SLSS / IQ<br>$r = -.08$, n.s,<br>0.64 % var. explained	Include data about SES.

Note. __ indicates that the information is not available in the article; (M) indicates that the study is a meta-analysis.

Table S9

Summary table of methodological aspects and effect sizes from articles on socialization.

Articles	Age Range	Sampling Methodology	Sample Size Gifted Group	Sample Size Non-Gifted Group	Effect Size and % of Variance Explained	Methodological Remarks
Cook et al., 2020	8 months - 11 years	Longitudinal Cohort<br>IQ ≥ 120	*n* = 192	*n* = 1 015	—	Parents are not informed of child's IQ test results, limiting the gifted labeling influence.
Peairs et al., 2019	Grades 6 - 12	Academic Achievement	*n* = 141	*n* = 186	- Achievement / Behavioral Adjustment Measures<br>partial $r = -.04$, *n.s*<br>0.16 % var. explained<br>To<br>partial $r = -.24$, $p < .001$<br>5.76 % var. explained<br>3.62 % Median var. explained<br>- Achievement / Perceived Popularity<br>partial $r = -.08$, n.s<br>0.64 % var. explained	Socioeconomic status as controlled variable.
Ryoo et al., 2017	Grades 5 - 9	Longitudinal Study<br>IQ ≥ 130	*n* = 299	*n* = 689	—<br>n.s	Gifted students spent most of their time in general education classrooms.

Articles	Age Range	Sampling Methodology	Sample Size Gifted Group	Sample Size Non-Gifted Group	Effect Size and % of Variance Explained	Methodological Remarks
Peyre et al., 2016	5 - 6 years	EDEN mother–child Cohort<br>Gr. 1: 70 < IQ<br>Gr. 2 : 70 < IQ ≤120<br>Gr. 3 : IQ > 130	Gr. 3: *n* = 23	Gr. 1: *n* =19<br>Gr. 2: *n* = 1058	Groups 2 and 3 / Total SDQ<br>*d* = .43, *p* = .045<br>4.41 % var. explained	—
Eklund et al., 2015	5 years - 12 years	Teacher Nomination within Gifted Programs	*N* = 1 206		Group / EBR<br>- Parents<br>*r* = - .05, *p* = .04<br>0.25 % var. explained<br>- Teachers<br>*r* = - .13, *p* = .00<br>1.69 % var. explained	Unspecified methodology regarding groups.

Articles	Age Range	Sampling Methodology	Sample Size Gifted Group	Sample Size Non-Gifted Group	Effect Size and % of Variance Explained	Methodological Remarks
Košir et al., 2015	11 - 15 years	Gifted Program<br>- Teachers' ratings<br>- IQ > 120<br>- Creativtiy	$n = 85$	$n = 319$	Analyses comparing gifted / control groups<br>Group / Social Acceptance<br>Partial $\eta^2 = .032$, $p = .005$<br>3.22 % var. explained<br>Group / Negative nominations<br>Partial $\eta^2 = .016$, $p = .01$<br>1.6 % var. explained<br>Group / Social Impact<br>Partial $\eta^2 = .012$, $p = .031$<br>1.2 % var. explained<br>Group / Self-concept<br>Partial $\eta^2 = .107$, $p < .001$<br>10.7 % var. explained<br>- Academic self-concept<br>Partial $\eta^2 = .074$, $p = .074$<br>7.4 % var. explained<br>- General self-concept<br>Partial $\eta^2 = .015$, $p = .013$<br>1.5 % var. explained<br>- Peer relations self-concept *n.s.*	Inclusion criteria within gifted programs lead to 26% of students being identified as gifted in the country where the study was conducted.<br>52% of the gifted participants were identified only on the basis of teacher assessment.<br>Means and standard deviations are given for gifted participants identified on the basis of an intelligence test and those assessed by teachers.
Masden et al., 2015	Grades 7 and 8	60 Pairs of Friendship Dyads<br>Gifted Program<br>- Academic Achievement	$n = 81$	$n = 39$	- Group / Psychosocial Competency<br>$\beta = .23$,<br>Semi-Partial $r^2 = .05$, $p < .05$<br>5.29 % var. explained<br>- Group / Friendship Quality<br>$\beta = -.24$, $sr^2 = .03$, $p < .01$<br>5.76 % var. explained	—

Articles	Age Range	Sampling Methodology	Sample Size Gifted Group	Sample Size Non-Gifted Group	Effect Size and % of Variance Explained	Methodological Remarks
Zeidner & Shani-Zinovich, 2015	Grades 10 - 12	Gifted Programs<br>- IQ<br>- Academic Achievement	*n* = 374	*n* = 428	Group / Social Self-Concept<br>Partial η^2 = 02, *p* < .01<br>2 % var. explained	—
Shechtman & Silektor, 2012	Grades 5 - 12	Gifted Programs<br>- Segregated Classes<br>- Pull-out Programs	*n* = 508 including<br>- *n* = 330 in Segregated Classrooms<br>- *n* =178 in Pull-out Programs	*n* = 466	- Groups / School level / Total Score on ICQ<br>η^2 = .01, *p* < .05<br>1 % var. explained<br>- Groups / School level / Total score on Assertiveness<br>η^2 = .02, *p* < .05<br>2 % var. explained	—
Simoes Loureiro et al., 2010	7 - 11 years	Clinical population<br>IQ > 125	*n* = 45	*n* = 30	—	—
López et al., 2009	4 - 17 years	IQ ≥ 130	*n* = 50	*n* = 50	Group / Self-concept Subscale Level of anxiety<br>Partial η^2 = .048, *p* < .05<br>4.8 % var. explained	No information was provided regarding the sampling method.<br>The instrument used to measure IQ was an outdated version of the Wechsler scales (WISC-R).

Articles	Age Range	Sampling Methodology	Sample Size Gifted Group	Sample Size Non-Gifted Group	Effect Size and % of Variance Explained	Methodological Remarks
Vialle et al., 2007	Grades 7 and 8	Academic Achievement	*n* = 65	*n* = Unspecified	—	Unspecified methodology.
Bain & Bell, 2004	Grades 4 - 6	Gifted Program<br>- Academic achievement<br>- IQ<br>- Teachers' ratings	*n* = 26	*n* = 67	—	Participants in the control group were high achievers, including *n* = 38 enrolled in a gifted program.
Richards et al., 2003	Grades 7 - 10	IQ ≥ 127	*n* = 33	*n* = 25	—	Use different ability tests to calculate participants IQ scores in the same gifted group.
Gallucci et al., 1999	12 - 16 years	- Summer Program for the Gifted<br>- Scholar Gifted Program<br>IQ > 130	Gifted: *N* = 78<br>Gifted from the summer program: *n* = 44<br>Gifted from the scholar program: *n* = 34	*n* = 62	—<br>n.s	—

Articles	Age Range	Sampling Methodology	Sample Size Gifted Group	Sample Size Non-Gifted Group	Effect Size and % of Variance Explained	Methodological Remarks
Garland & Zigler, 1999	Mostly Grades 9	Summer Program for the Gifted, based on SAT	$n = 191$	Normative data	—	No control group.
Norman et al., 1999	Grades 6 - 8	Summer Program for the Gifted<br>- IQ ≥ 125<br>- Achievement Criterion<br><br>Highly Gifted: School Ability Index > 132<br>Gifted: 116 < SAI ≤ 132	Highly Gifted: $n = 74$<br><br>Moderately Gifted: $n = 163$	Normative data	—<br>n.s	No control group.
Field et al., 1998	High School Freshmen	Gifted Program based on IQ ≥ 132	$n = 62$	$n = 162$	—	—
Swiatek, 1995	Grades 7 - 10	Summer Program for the Gifted	$n = 210$	—	Ability level / Deny Giftedness Strategy<br>$d = .64$<br>9.30 % var. explained	No control group.

Articles	Age Range	Sampling Methodology	Sample Size Gifted Group	Sample Size Non-Gifted Group	Effect Size and % of Variance Explained	Methodological Remarks
Cohen et al., 1994	Grades 4 - 6	Gifted Pull-out Program<br>- Academic achievement<br>- IQ > 127	*n* = 53	*n* = 149	—	—
Czeschlik & Rost, 1994		121 ≤ IQ ≤134	*n* = 50	*n* = 50	Group / Perceived Socialization<br>*d* = .48<br>5.43 % var. explained	—
Daubert & Benbow, 1990	13 years	Achievement<br>Highly Gifted:<br>SAT-M ≥ 700<br>SAT-V ≥ 630<br>Moderately Gifted:<br>SAT-M + SAT-V ≤ 540	Highly Gifted:<br>*n* = 300<br>Moderately Gifted:<br>*n* = 111	—	Group / Social Abilities<br>$r = .58$, $p < .001$<br>33.64 % var. explained<br>To<br>$r = .67$, $p < .001$<br>44.89 % var. explained<br>36 % Median var. explained	No control group.
Gallucci, 1988	12 - 16 years	- Summer Program for the Gifted<br>- Scholar Gifted Program<br>Higlhy Gifted: IQ >150<br>Gifted: 135 < IQ ≤ 140	Highly Gifted:<br>*n* = 49<br>Moderately Gifted:<br>*n* = 34	Normative data	—<br>n.s	No control group.

Articles	Age Range	Sampling Methodology	Sample Size Gifted Group	Sample Size Non-Gifted Group	Effect Size and % of Variance Explained	Methodological Remarks
Brody & Benbow, 1986	Grades 7 and 8	Achievement<br>Highly Gifted: SAT-M ≥ 700 SAT-V ≥ 630<br>Moderately Gifted: SAT-M + SAT-V ≤ 540	Highly Gifted: $n = 300$<br>Moderately Gifted: $n = 111$	—	Group / Social Abilities<br>$f = .44$, $p < .001$<br>16.24 % var. explained	No control group.
Janos & Robinson, 1985	—	Newspapers Publicity and Self-Selected Volunteers<br>Highly Gifted: IQ > 163<br>Gifted: 120 < IQ ≤ 140	Highly Gifted: $n = 32$<br>Moderately Gifted: $n = 27$	—	—	No control group.
Lehman & Erdwins, 1981	Grades 3 (and Grades 6 for the Non-Gifted Group)	Gifted Program<br>141 ≤ IQ ≤ 165	$n = 16$	$n = 32$	—	Principals' nomination of participants included in the non-gifted groups.
Milgram & Milgram, 1976	Grades 4 - 8	Gifted Program - After School Classes<br>IQ ≥ 135	$n = 182$	$n = 310$	—	—

Note. __ indicates that the information is not available in the article; (M) indicates that the study is a meta-analysis.

Table S10

Summary table of methodological aspects and effect sizes from articles on self-esteem (SE).

Articles	Age Range	Sampling Methodology	Sample Size Gifted Group	Sample Size Non-Gifted Group	Effect Size and % of Variance Explained	Methodological Remarks
Casino-García et al., 2021	8-18 years	Gifted identified by licensed psychologist	$n = 118$	$n = 122$	Gifted students SE scores < non-identified peers scores, $p = .03$	—
Fanaj & Mustafa, 2021	13-19 years	Nominated as gifted by teachers	$n = 960$	$n = 649$	— n.s	—
Papadopoulos, 2021	5-6 years	IQ > 120	$N = 108$		IQ/global SE, $r = .201, p < .05$ 4.04% var. explained<br><br>Scholastic competence/global SE, $r = .214, p < .05$ 4.58% var. explained	—
Bakar, 2020	12-17 years	- National Gifted and Talented Center, University of Kebangsaan Malaysia	$N = 194$		— n.s	Gifted and Talented students have medium to high SE.

Articles	Age Range	Sampling Methodology	Sample Size Gifted Group	Sample Size Non-Gifted Group	Effect Size and % of Variance Explained	Methodological Remarks
Moyano et al, 2020	6-9 years	Academic performance was evaluated with the average Grades	*N* = 133		- SE is a predicator of academic performance β = 0.403, $p < 0.001$<br>- SE/academic Math $r = .32$, $p < .001$ 10.24% var. explained<br>- SE/academic language $r = .32$, $p < .001$ 10.24% var. explained<br>- SE/reasoning $r = .21$, $p < .05$ 4.41% var. explained	—
Li & Shi, 2019	8 to 11 years old	- Gifted program<br>- Stanford-Binet, Weschler<br>- Raven's Standard Progressive Matrices	$n = 80$	$n = 104$	Gifted SE scores<br>> Control group SE scores,<br>t = 2.50, p = 0.013	—
Yang et al., 2019	Grades 3-5	Academic achievement : scores in Chinese, Math, and English	N = 779		SE / achievement<br>$r = .23$ to $r = .30$<br>From 5.29% to 9% var. explained	—

Articles	Age Range	Sampling Methodology	Sample Size Gifted Group	Sample Size Non-Gifted Group	Effect Size and % of Variance Explained	Methodological Remarks
Giofrè et al., 2017	Grades 6-8	- Cattell Culture Fair Intelligence Test<br>- INVALSI test	*N* = 159		- Indirect effect of SE on mathematic β = .155, *p* = .033; overall effect of SE on mathematic β = .317, *p* < .001<br>- Indirect effect of SE on reading β = .118, *p* = .033; overall effect of SE on reading β = .588, *p* < .001	—
Tetzner et al., 2017	Grades 7–10	Scholastic achievement in Mathematics, English and physics	*N* = 7977		Academic achievement / SE<br>*r* between .23 and .41, *p* < .001<br>From 5.29% to 16.81% var. explained<br>Higher academic achievement predict higher SE, but not vice versa	Longitudinal study
Preckel et al., 2016	Grades 10-12	- Gifted summer school (based on scholastic achievement, motivation and engagement)	*n* = 177	—	Not significant evolution of SE aver time	No control group.
Topçu & Leana Tascilar, 2016	Grades 4-8	After-school program for gifted students or public special education school for gifted students.	*n* = 184	—	General SE explained 5% of achievement in fourth Grades and academic SE 9%.	No control group.

Articles	Age Range	Sampling Methodology	Sample Size Gifted Group	Sample Size Non-Gifted Group	Effect Size and % of Variance Explained	Methodological Remarks
Kaya & Ogurlu, 2015	Middle school students	- Raven Standard Progressive Matrices<br>- GPA	*N* = 127		No significant relationship between SE and intelligence	—
Diseth et al., 2014	Grades 6 and 8	Grades in mathematics, Norwegian and English	*N* = 2062		Accademic achievement level correlated positively with SE,<br>$r = .25, p < .01$<br>6.25% var. explained	—
Di Giunta et al., 2013	Junior and senior high school	- Academic achievement at the end of the junior high school (8th Grades) assessed by their teacher<br>- Academic performance at the end of the senior high school (self-report)	*N* = 426		Junior high-school Grades/SE<br>*r* between .16 and .20, $p < .05$<br>From 2.56% to 4 % var. explained<br>Senior high-school Grades/SE<br>*r* between .16 and .17, $p < .05$<br>From 2.56% to 2.89% var. explained	—
Foley-Nicpon, 2012	6-18	IQ > 120	*n* = 112<br>54 diagnostic criteria for ADHD	Normative data	Gifted with ADHD had lower scores on measures of SE	No control group.
Zuffianò et al., 2012	Grade 8	- Cultural-Fair Intelligence Test	*N* = 170		SE uncorrelated with academic achievement	—

Articles	Age Range	Sampling Methodology	Sample Size Gifted Group	Sample Size Non-Gifted Group	Effect Size and % of Variance Explained	Methodological Remarks
Courtinat-Camps et al., 2011	9-15 years	IQ > 130	*n* = 255<br>204 enrolled in homogeneous groups, 51 in heterogeneous groups	—	Gifted SE in homogeneous grouping < Gifted SE in heterogeneous grouping $p < .01$, $\eta^2 = .055$	—
Marsh & O'Mara, 2008	Grade 10 to post-graduation	Longitudinal study<br>National Youth in Transition database			—<br>n.s<br>only the path from T2 self-esteem to T3 school Grades (.07) is marginally significant	—
Pullmann & Allik, 2008	Grades 2-12 and university applicants	Academic achievement measured through GPA	*N* = 4572		SE / Academic achievement from r =.28 up to .42 ($p < .001$) from 2nd Grades to 6th Grades and decline rapidly afterwards to not significant for 12th Grades and university applicants<br>From 7.84 to 17.64% var. explained	—
Benony et al., 2007	8-13 years	IQ > 130	*n* = 23	*n* = 23	—	—

Articles	Age Range	Sampling Methodology	Sample Size Gifted Group	Sample Size Non-Gifted Group	Effect Size and % of Variance Explained	Methodological Remarks
Ghobary & Hejazi, 2007	Middle school	IQ > 130	*n* = 60	*n* = 60	SE / Academic achievement $r = .36$, $p < .005$ for gifted students 12.96 % var. explained<br>$r = .14$, *n.s* for regular students<br>Gifted group > control group, $p < .05$	For the authors, correlation coefficients are interpreted as large when they approximate .30.
Vialle et al., 2007	Secondary students	Top 10% of Student in the ELLA and SNAP scores	*n* = 65	*n* = ~ 800	SE unrelated to academic Grades for gifted students	—
Marsh & Craven, 2006						Review of the litterature
Vialle et al., 2005	Secondary student	Top 10% of Student in the ELLA and SNAP scores	*n* = 65	*n* = ~ 800	No differences in measured SE between gifted and non-gifted students.<br>No correlation between SE and academic achievement for the gifted group $r = .02$ (ns)	—

Articles	Age Range	Sampling Methodology	Sample Size Gifted Group	Sample Size Non-Gifted Group	Effect Size and % of Variance Explained	Methodological Remarks
Adams-Byers et al., 2004	Grades 5-11	- Summer programs for gifted and talented youth<br>- IQ > 124 (Grades 4-6)<br>- SAT verbal > 430, SAT Math > 500 (Grades 6-8)<br>- SAT Verbal and Math > 500 (Grades 9 – 12)	*n* = 44	—	—	Qualitative study<br>No control group.
Baumeister et al., 2003						Review of the litteratue
D'Amico & Cardaci, 2003	Mean age 13.4 years old (SD=1.1)	- Teachers rates (Linguistic-Literary, Logical-Mathematical, and Technical-Practical scores)	*N* = 151		SE/Academic performance<br>—<br>n.s	—
Schmidt & Padilla, 2003	Grades 10-12	self-reported Grades in school	*N* = 330		SE 10th Grades/Aca Grades 10th Grades, $r = .15$, $p < .01$<br>SE 12th Grades/Aca Grades 10th Grades, $r = .15$, $p < .01$<br>SE 10th Grades/Aca Grades 12th Grades, *NS*<br>SE 12th Grades/Aca Grades12h Grades, $r = .17$, $p < .01$<br>From 2.25% to 2.89 % var. explained	—

Articles	Age Range	Sampling Methodology	Sample Size Gifted Group	Sample Size Non-Gifted Group	Effect Size and % of Variance Explained	Methodological Remarks
Ross & Broh, 2000	Grades 8-12	Curriculum-based achievement tests in mathematics, science, reading, and history	*N* = 8802		Covariance structure model: academic achievement / SE r =.191 3.65 % var. explained SE does not significantly improve Grades	—
Roznowski et al., 2000	Grade 10	Longitudinal Study	*n* = 640	*n* = 10 096	Group / SE Gifted group score > Average group score	No information on signiticativity or effect size.
Faouri, 1998	Grades 3-7	Gifted program	*n* = 20 including 10 students with learning disabilities	*n* = 20 including 10 students with learning disabilities	Gifted group SE scores > Control group SE scores, *p* = .028	—
Field et al., 1998	Grades 5-9	Gifted Program IQ ≥ 132	*n* = 62	*n* = 162	— n.s	—

Articles	Age Range	Sampling Methodology	Sample Size Gifted Group	Sample Size Non-Gifted Group	Effect Size and % of Variance Explained	Methodological Remarks
Lea-Wood & Clunies-Ross, 1995	Grades 7-9	Ability test and/or nomination by teacher	*n* = 81	*n* = 77	Non-gifted girls obtained higher Total Self-esteem means $F(1,152) = 12.013$, $p = .001$ The difference between the means of the giftedness groups was negligible at year 7 but was large at years 8 and 9.	Only girls were included in the sample.
Ball et al., 1994	12 to 15	IQ > 135	*n* = 61	*n* = 122	Group / SE as a decision maker $F(1,170) = 19.98$, $p < .001$	—
Van Tassel-Baska et al., 1994	Grades 7-8	IQ > 120, > 95th percentile in either mathematical of verbal areas	*n* = 147	—	High SE among gifted students > average score	No control group.
Kulik & Kulik, 1992 (M)		13 of the 56 studies described effects of grouping on student SE Eleven of the 13 studies also reported results separately by ability level.			average overall effect of grouping: decrease self-esteem scores by 0.03 SD Effect sizes by ability level: High: - .15 Medium: - .09 Low: .19	Unspecified methodology.

Articles	Age Range	Sampling Methodology	Sample Size Gifted Group	Sample Size Non-Gifted Group	Effect Size and % of Variance Explained	Methodological Remarks
Chiu, 1990	Grades 4-5	- Otis-Lennon IQ score (from 115 to 148, average 129)<br>- teacher nomination<br>- Achievement test scores (Iowa Tests of Basic Skills) > 95 th percentile or above	*n* = 136	*n* = 196<br>*n* = 118 mild mentally handicapped	Main effect of Academic ability<br>$F(2,444) = 18.87$, $p < .01$<br>No difference between gifted and normal children Self-Esteem > mild mentally handicapped	—
Pearson & Beer, 1990	Elementary children	- intelligence scores at the 97th percentile<br>- academic scores at the 95th percentile on individually administered tests	*n* = 38	Normative data	—<br>n.s	No control group.
Alsaker, 1989	Grades 6-9	Grades in Norwegian, English and Mathematics	*N* = 2309		General SE/average Grades<br>*r* from -.10 to -.30<br>from 1% to 9 % var. explained	—
Rosenberg et al., 1989	Grade 10	School performance measured by student's self-reported Grades point average	*N* = 1886		Correlation SE/Grades between *r* = .24 and .25<br>From 6.25% to 5.76 % var. explained<br>Significant effect of Grades on SE +.15. effect of SE on Grades Ns (t = 1.90)	All-boys sample.

Articles	Age Range	Sampling Methodology	Sample Size Gifted Group	Sample Size Non-Gifted Group	Effect Size and % of Variance Explained	Methodological Remarks
Chan, 1988	Grades 5-7	- Gifted programs and teacher / parent nomination<br>- Raven's Progressive Matrices<br>- Test of learning ability	*n* = 117	*n* = 261	$F(2,182) = 9.50$, $p < .0001$	—
Colangelo et al., 1987	Grades 7-9	- composite scores WISC,<br>- Iowa Test Basic Skills<br>- Grades point average	*n* = 61	*n* = 162<br>*n* = 20 students with special learning needs	—<br>n.s	—
Cornell & Grossberg, 1987	7 to 11	- Gifted program<br>- Wechsler or Stanford-Binet scale<br>mean 139.9 (SD 11.22)	*n* = 83	Normative data	Group / SE<br>t value = 12.04, $p < .01$	No control group.
Bartell & Reynolds, 1986	Grades 4-5		*n* = 111	*n* = 34	—<br>n.s	Unspecified methodology.
Brody & Benbow, 1986	Grades 7 and 8	Achievement<br>Highly Gifted:<br>SAT-M ≥ 700<br>SAT-V ≥ 630<br>Moderately Gifted:<br>SAT-M + SAT-V ≤ 540	Highly Gifted:<br>*n* = 300<br>Moderately Gifted:<br>*n* = 111	—	Group / SE<br>n.s between gifted students and normative data<br>Highly gifted SE scores > Gifted SE scores<br>$f = .19$, $p < .01$<br>3.48% var. explained	No control group.

Articles	Age Range	Sampling Methodology	Sample Size Gifted Group	Sample Size Non-Gifted Group	Effect Size and % of Variance Explained	Methodological Remarks
Hansford & Hattie, 1982<br>(M)	School age	128 studies	N =202,823		SE / Achievement<br>Average correlation .21<br>From -.77 to .96<br>4.41 % var. explained	Data base of 1,136 correlations.<br>No information about age or Grades.
McEwin & Cross, 1982	Grades 5-8	- IQ > 120 +<br>- teacher identification as exhibiting outstanding leadership and/or talent	n = 115	n = 260	—<br>n.s	—
Winne et al., 1982	Grades 4-7	- general ability as reflected by the Peabody Picture Vocabulary Test<br>- reading comprehension subtest of the Canadian Test of Basic Skills<br>- teachers' identification.	n = 58	n = 60 average students<br>n = 52 learning disabled students	—<br>n.s	—
Lehman & Edwins, 1981	Grade 3<br>(and Grades 6 for the Non-Gifted Group)	Gifted Program<br>141 ≤ IQ ≤ 165	n = 16	n = 32	Gifted group SE scores > Control group SE scores,<br>$p < .05$	Principals nomination of participants included in the non-gifted groups.

Articles	Age Range	Sampling Methodology	Sample Size Gifted Group	Sample Size Non-Gifted Group	Effect Size and % of Variance Explained	Methodological Remarks
Tidwell, 1980	Grade 10<br>14 to 17	- Identified by district school psychologists as gifted (to 2% of all student)<br>Mean IQ: 136.99 (SD=15.25)	*n* = 1593	Normative data	—<br>n.s	No control group.
Dean, 1977	Grades 7-8	- Enrichisment program for the gifted<br>- Lorge-Thorndike Intelligence test<br>IQ means:<br>147.9, sd = 13.4 for girls;<br>138.5, sd = 12.6 for boys	*n* = 48	Normative data	—<br>n.s	No control group.
Lewis & Adank, 1975	Grades 4-6	-IQ scores from the SRA Tests of General Ability<br>-Achievement with SAT	*N* = 219		IQ/SE<br>*r* between .24 and .34, p <0.05<br>From 5.76% to 11.56 % var. explained<br>SAT/SE<br>*r* between .30 and .42, p <0.01<br>From 9% to 17,64% var. explained	—

Articles	Age Range	Sampling Methodology	Sample Size Gifted Group	Sample Size Non-Gifted Group	Effect Size and % of Variance Explained	Methodological Remarks
Simon & Simon, 1975	Grade 5<br>10 to 12 years	- Verbal and nonverbal IQ<br>- SRA Achievement Series	*N* = 87		SE/Verbal IQ<br>*r* =.0.30, *p* <.01<br>9 % var. explained<br>SE/Non verbal IQ<br>*r* = 0.23, *p* <.05<br>5.29 % var. explained<br>SE/achievement<br>*r* =.33, *p* <.01<br>10.89 % var. explained	—
Coopersmith, 1967					SE/ intelligence<br>*r* = .28, *p* < .05<br>7.84 % var. explained<br>SE/ achievement<br>*r* = .30, *p* < .05<br>9 % var. explained	—

Note. __ indicates that the information is not available in the article; (M) indicates that the study is a meta-analysis.

Table S11

Summary table of methodological aspects and effect sizes from articles on humor.

Articles	Age Range	Sampling Methodology	Sample Size Gifted Group	Sample Size Non-Gifted Group	Effect Size and % of Variance Explained	Methodological Remarks
Bianchi et al., 2017	12 - 15 years	98th percentile<br>- WISC-IV<br>AND<br>- Progressive Matrices of Raven	*n* = 23	*n* = 78	- Irony Understanding<br>*d* = .58, *p* = .011<br>7.78 % var. explained<br><br>- Irony Production<br>*d* = .66, *p* = 0.015<br>9.80 % var. explained	—
Willinger et al., 2017	Mean age = 33.4 years (SD = 11.9)	Verbal and Nonverbal intelligence:<br>Vocabulary test and Number-Connection-Test respectively	*N* = 156		Black humor / intelligence<br>*NS*	
Christensen et al., 2016	College Students	—	*N* = 270		- Humor / g<br>β = .51, *p* < .001<br>26.01 % var. explained<br><br>- Humor / Gc<br>*r* = .49, *p* < .001<br>24.01 % var. explained<br><br>- Humor / Gf<br>*r* = .22, *p* = .016<br>4.84 % var. explained	Distinction between Gf and Gc in the analyses.

Articles	Age Range	Sampling Methodology	Sample Size Gifted Group	Sample Size Non-Gifted Group	Effect Size and % of Variance Explained	Methodological Remarks
Kellner & Benedek, 2016	College Students	Mailing and Announcements on University Campus	$N = 151$		- Humor / Intelligence<br>$r = .30$, $p < .01$<br>9 % var. explained<br><br>- Humor / Gf<br>$r = .13$<br>$\beta = .00$, $p = .56$<br>1.69 % var. explained<br><br>- Humor / Gc<br>$r = .37$<br>$\beta = .29$, $p = .001$<br>13.69 % var. explained	Distinction between Gf and Gc in the analyses.
Sharifi & Sharifi, 2014	Grades 10 - 12	__	$n = 60$	$n = 60$	__	Unspecified methodology.<br><br>Only females participants.
Vrticka et al., 2013	6 - 13 years	94 ≤ IQ ≤ 140, mean IQ 121,6	$N = 22$		__	__
Bergen, 2009	Grades 4 - 6	__	$n = 74$	__	__	Structured Interview Method.<br><br>No control group.

Articles	Age Range	Sampling Methodology	Sample Size Gifted Group	Sample Size Non-Gifted Group	Effect Size and % of Variance Explained	Methodological Remarks
Shade, 1991	Grades 4, 6 and 8	Gifted Program<br>IQ ≥ 130	*n* = 60	*n* = 60	—	—
Ziv, 1990	Grade 8 - 12	IQ > 130	*n* = 30	*n* = 62	—	Unspecified methodology.
Barnett & Fiscella, 1985	Preschool children	IQ ≥ 130	*n* = 15	*n* = 20	—<br>n.s	—
Hauck & Thomas, 1972	Grades 4 - 6	107 ≤ IQ ≤ 144	*N* = 80		Humor / IQ<br>$r = .91, p < .005$<br>82.81 % var. explained	Unspecified methodology.

Note. __ indicates that the information is not available in the article; (M) indicates that the study is a meta-analysis.

Table S12

Summary table of methodological aspects and effect sizes from articles on interests.

Articles	Age Range	Sampling Methodology	Sample Size Gifted Group	Sample Size Non-Gifted Group	Effect Size and % of Variance Explained	Methodological Remarks
Roznowski et al., 2000	Grade 10	Longitudinal Study	$n = 640$	$n = 10\ 096$	Group / Hobby<br>$h = .30$<br>2.19 % var. explained<br>To<br>$h = .52$<br>6.35 % var. explained<br>4.62 % Median var. explained	__
Lehman & Witty, 1927	Grades 3 - 7	Gifted Group:<br>IQ ≥ 140<br>Non-Gifted Group:<br>90 ≤ IQ ≤ 110	$n = 50$	$n = 50$	__	__

Note. __ indicates that the information is not available in the article; (M) indicates that the study is a meta-analysis.

Table S13

Summary table of methodological aspects and effect sizes from articles on moral development.

Articles	Age Range	Sampling Methodology	Sample Size Gifted Group	Sample Size Non-Gifted Group	Effect Size and % of Variance Explained	Methodological Remarks
Lee et al., 2020	Junior High School Students	Achievement	*n* = 1 062	*n* = 614	Groups / Social Purpose<br>$\eta^2 = .03$, $p < .001$<br>3 % var. explained	Participants are Korean and American.<br><br>SES was controlled in some analyses.
Beißert & Hasselhorn, 2016	6 - 8 years	Gifted Program<br>- Teacher Nomination	*n* = 62	*n* =67	- Correlation Intelligence / Moral Reasoning<br>$r = .01$, n.s<br>0,01 % var. explained<br>To<br>$r = - .12$, n.s<br>1.44 % var. explained<br>0.52 % Median var. explained<br><br>- Intelligence as a Covariate in Moral Reasoning<br>$\eta^2 = 00$, n.s<br>0 % var. explained<br>To<br>$\eta^2 = 02$, n.s<br>2 % var. explained<br>0.5 % Median var. explained	—

Articles	Age Range	Sampling Methodology	Sample Size Gifted Group	Sample Size Non-Gifted Group	Effect Size and % of Variance Explained	Methodological Remarks
Alnabhan, 2011	Grades 8 and 11	≥ 90% on Ravens Matrices Test	n = 73	n = 159	- Non-verbal IQ / Moral Judgement<br>β = .02, n.s<br>0.04 % var. explained<br><br>- Achievement / Moral Judgement<br>β = .10, n.s<br>1 % var. explained	—
Derryberry & Barger, 2008	- Grades 7 – 10<br>- College Students	Achievement	n = 30	n = 30	- Achievement / DIT<br>r = .15, n.s<br>2.25 % var. explained<br><br>- Group / DIT<br>η^2 =.29, 29 % var. explained<br>d = 1.07, p < .001<br>22.28 % var. explained	—
Tiri & Nokelainen, 2007	Grades 7 - 9	Achievement<br>8,5 ≤ GPA ≤10	n = 130	n = 114	—	—

Articles	Age Range	Sampling Methodology	Sample Size Gifted Group	Sample Size Non-Gifted Group	Effect Size and % of Variance Explained	Methodological Remarks
Lee & Olszewski-Kubilius, 2006	Grades 10 - 12	Summer Camp for Gifted based on Achievement (SAT)	*n* = 234	Normative data	- SAT / DIT<br>*r* = - .05, n.s<br>0.25 % var. explained<br>To<br>*r* = .06, n.s<br>0.36 % var. explained<br>0.36 % Median var. explained<br><br>- Group / DIT<br>*d* = .04, n.s<br>0.04 % var. explained<br>To<br>*d* = .50, n.s<br>5.9 % var. explained<br>0.30 % Median var. explained	No control group.<br><br>121 gifted participants come from the civic leadership institue.
Derryberry et al., 2005	- Grades 7 – 10<br>- College Students	Gifted Program<br>- Achievement (SAT-M and V > 500)	*n* = 97	*n* = 140	Group / DIT<br>η^2 =.05, *p* = .005<br>5 % var. explained	—
Tiri & Pehkonen, 2002	Grades 8 and 9	After School Gifted Program<br>- Teacher Nomination<br>- Ravens Matrices Test	*n* = 31	Normative data	Raven Test / DIT<br>*r* near 0<br>0 % var. explained	No control group.<br>The study is semi-qualitative and based in part on interviews.

Articles	Age Range	Sampling Methodology	Sample Size Gifted Group	Sample Size Non-Gifted Group	Effect Size and % of Variance Explained	Methodological Remarks
Howard-Hamilton & Franks, 1995	Senior High School Students	Summer Camp for Gifted and Talented based on Achievement	*n* = 167	Normative data	—	—
Chovan & Freeman, 1993	Grades 5, 8 and 10	—	*n* = 51	*n* = 50	—	Unspecified methodology.
Narvaez, 1993	Grade 8	Achievement	Private Preparatory School: *n* = 69<br>Suburban School: *n* = 53	—	Scholastic Scores / DIT<br>*r* = .28<br>7.84 % var. explained<br>To<br>*r* = .36<br>12.96 % var. explained<br>10.93 % Median var. explained	No control group.<br>Mostly descriptive analyses.
Simmons & Zumpf, 1986	4 - 7 years	Intelligence Test	*n* = 38	Normative data	—	No control group.
Tan-Willman & Gutteridge, 1981	Secondary School Students	- Acceleration Program<br>- Academic Achievement	*n* = 115	Empirical studies	—	No control group.<br>Unspecified methodology.

Articles	Age Range	Sampling Methodology	Sample Size Gifted Group	Sample Size Non-Gifted Group	Effect Size and % of Variance Explained	Methodological Remarks
Karnes & Brown, 1980	Grades 5 - 9	Gifted Program<br>- IQ ≥ 120	$n = 233$	—	- Intelligence Measures / DIT<br>$r = -.06$, n.s<br>0.36 % var. explained<br>To<br>$r = .31$, $p < .01$<br>9.61 % var. explained<br>4.10 % Median var. explained	No control group.
Kohlberg, 1964	—	—	—	—	IQ / moral judgement<br>$r = .31$<br>9.61 % var. explained	—

Note. __ indicates that the information is not available in the article; (M) indicates that the study is a meta-analysis.

Table S14

Summary table of methodological aspects and effect sizes from articles on leadership.

Articles	Age Range	Sampling Methodology	Sample Size Gifted Group	Sample Size Non-Gifted Group	Effect Size and % of Variance Explained	Methodological Remarks
Lee et al., 2020	Secondary Students	- Academic achievement<br>- Talent development center	*n* = 440	*n* = 303	- Group / Type of leadership<br>$r = .15$, $p = .001$<br>2.25 % var. explained<br><br>- Group / Leader's ability<br>$r = .24$, $p < .001$<br>5.76 % var. explained	Participants are Korean and American adolescents.<br><br>The design includes a focus group interview.
Peairs et al., 2019	Grade 7	Achievement	*n* = 202	*n* = 272	—	—
Muammar, 2015	College Students	Ability Test<br>GAT ≥ 80	*n* = 56	*n* = 120	Group / Planning skills<br>$d = .31$, $p = .04$<br>2.34 % var. explained	—
Hoffman et al., 2011<br>(M)	—	187 Studies	*N* = 146 851		- Leader Effectiveness / Cognitive Abilities<br>$\rho = .17$<br>2.89 % var. explained<br><br>- Leader Effectiveness / Problem-Solving Skills<br>$\rho = .39$<br>15.21 % var. explained	—

Articles	Age Range	Sampling Methodology	Sample Size Gifted Group	Sample Size Non-Gifted Group	Effect Size and % of Variance Explained	Methodological Remarks
Mills, 2009<br>(M)	—	48 Studies	*N* = 7 343		Leadership / Emotional Intelligence<br>Combined Effect $r = .38$, with r ranging from.00 to .90<br>14.44 % var. explained	Unpublished dissertations (48%) and theses (8%) were included in the meta analysis.<br>Lack of information regarding ages of the participants.
Lee & Olszewski-Kubilius, 2006	Grades 10 - 12	Summer Program for the Gifted based on Achievement (SAT)	$n = 234$	Normative data	- SAT-M / Leadership<br>$r = -.19$, $p = .05$<br>3.61 % var. explained<br>- SAT-Combined / Leadership<br>$r = -.20$, $p = .04$<br>4 % var. explained<br>- Group / Leadership<br>$d = .67$<br>10.11 % var. explained	No control group.<br>121 gifted participants come from the civic leadership institue.

Articles	Age Range	Sampling Methodology	Sample Size Gifted Group	Sample Size Non-Gifted Group	Effect Size and % of Variance Explained	Methodological Remarks
Judge et al., 2004<br>(M)	—	151 Studies	*N* = 40 652		- Leadership / Intelligence<br>Average *r* = .17<br>2.89 % var. explained<br><br>- Leadership / Perceived Assessments of Intelligence<br>*k*-weighted average of .60<br><br>- Leadership / Pencil-and-Paper Assessments of Intelligence<br>*k*-weighted average of .18	—
Lord et al., 1986<br>(M)	High School Students to Adults	18 Studies	*N* = 2 239		Intelligence / Leadership<br>Corrected *r* = .50<br>25 % var. explained	—

Note. __ indicates that the information is not available in the article; (M) indicates that the study is a meta-analysis.

Table S15

Summary table of methodological aspects and effect sizes from articles on emotional intelligence trait.

Articles	Age Range	Sampling Methodology	Sample Size Gifted Group	Sample Size Non-Gifted Group	Effect Size and % of Variance Explained	Methodological Remarks
MacCann et al., 2020<br><br>(M)	Children to Adults	162 Studies<br>Academic Performance	—		- EI Self-rated Measures / Academic Performance<br>*r* = .10, *p* < .001<br>1.44 % var. explained<br><br>- EI Mixed Measures / Academic Performance<br>*r* = .13, *p* < .001<br>3.61 % var. explained	All studies included report effect sizes.
Sánchez-Álvarez et al., 2020<br><br>(M)	Secondary Students	44 Studies<br>Academic Performance	*N* = 19 861		- EI Self-rated Measures / Academic Performance<br>*r* = .26<br>6.76 % var. explained<br><br>- EI Mixed Measures / Academic Performance<br>*r* = .24<br>5.76 % var. explained	—
Li & Shi, 2019	8 to 11 years old	- Gifted program<br>- Stanford-Binet, Weschler<br>- Raven's Standard Progressive Matrices	*n* = 80	*n* = 104	Gifted EI scores / Control group EI scores, *NS*<br>RSPM / EI in gifted group<br>*r* = - .18<br>RSPM / EI in control group<br>*r* = .07	—

Articles	Age Range	Sampling Methodology	Sample Size Gifted Group	Sample Size Non-Gifted Group	Effect Size and % of Variance Explained	Methodological Remarks
Li et al., 2017	7 - 11 years	Gifted Program<br>33 ≤ IQ ≤ 43 on Cattel's Culture Fair Intelligence Test	*n* = 98	*n* = 125	- EI Score / Gifted Group<br>$r = .22, p < .05$<br>4.84 % var. explained<br>- EI Score / Non-Gifted Group<br>$r = .28, p < .01$<br>7.84 % var. explained	—
Perera & DiGiacomo, 2013<br>(M)	Children to Young Adults	40 Studies<br>Academic Performance	*N* = 10 292		EI / Academic Performance<br>Summary effect $r = .20$<br>4 % var. explained	12 unpublished dissertations were included in the meta-analysis.
Brasseur & Grégoire, 2010	11 - 19 years	Clinical Population<br>- IQ > 125<br>OR<br>- Verbal IQ > 130	*n* = 90	*n* = 90	- EI / Academic Achievement<br>R^2 adjusted = .07, $p < .00$<br>7.92 % var. explained<br>- EI / Group<br>_<br>n.s	—
Lee & Olszewski-Kubilus, 2006	Grades 10 - 12	Summer Program for the Gifted based on Achievement (SAT)	*n* = 234	Normative data	EI Score / Group<br>- Males<br>$d = - .12$, n.s<br>0.36 % var. explained<br>- Females<br>$d = - .42, p = .00$<br>4.24 % var. explained	No control group.<br>121 gifted participants come from the civic leadership institue.

Articles	Age Range	- Sampling Methodology	Sample Size Gifted Group	Sample Size Non-Gifted Group	Effect Size and % of Variance Explained	Methodological Remarks
Schwean et al., 2006	Grades 4 – 8	- Gifted Program<br>- Regular Classes<br>IQ ≥ 130	*n* = 169	*n* = 1200	__	__
Corso, 2001<br>Masters thesis	12 - 16 years	Summer Program for the Gifted based on Achievement	*n* = 100	Normative data	__	No control group.

Note. __ indicates that the information is not available in the article; (M) indicates that the study is a meta-analysis.

Table S16

Summary table of methodological aspects and effect sizes from articles on emotional intelligence ability.

Articles	Age Range	Sampling Methodology	Sample Size Gifted Group	Sample Size Non-Gifted Group	Effect Size and % of Variance Explained	Methodological Remarks
Perpiña Martí et al., 2023	8-11 years	Academic achievement<br>(language and mathematics)	$N = 173$		Academic achievement in mathematics / EI score<br>$r = .188$<br>Academic achievement in language / EI score<br>$r = .043$	—
Abdulla Alabbasi et al., 2020<br><br>(M)	Grades 4 - 12	21 Studies Involving Gifted Participants	$N = 15\ 455$	$N = 27\ 464$	- EI Level Mean Effect Size / Group<br>$g = .23, p < .001$<br>1.30 % var. explained<br><br>- EI Ability Measures (MSCEIT) / Group<br>$g = .43, p < .001$<br>4.41 % var. explained<br><br>- EI Trait Measures (EQ-i) / Group<br>$g = .22, p < .001$<br>1.19 % var. explained<br><br>- EI Trait Measures (SSEIT) / Group<br>$g = - .015$, n.s<br>0 % var. explained	Dissertation, theses (4) and conference proceeding (2) were included in the meta analysis.<br><br>No control of the dependency of multiple effect sizes within study.

Articles	Age Range	Sampling Methodology	Sample Size Gifted Group	Sample Size Non-Gifted Group	Effect Size and % of Variance Explained	Methodological Remarks
MacCann et al., 2020<br><br>(M)	Children to Adults	162 Studies<br><br>Academic Performance	—	—	EI Ability / Academic Performance<br>$r = .16$, $p < .001$<br>2.56 % var. explained	All studies included report effect sizes.
Ogurlu, 2020<br><br>(M)	Grade 1 to Adulthood	16 Studies,<br><br>Including 11 Studies focus on Children and Adolescents.	—	—	- EI Level Overall Effect Size / Group<br>$g = .12$, $p = .02$<br>0.36 % var. explained<br><br>- EI Ability Measures / Group<br>$g = .33$, $p = .05$<br>2.66 % var. explained<br><br>- EI Trait Measures / Group<br>$g = .04$, $p = .47$<br>0.04 % var. explained	—
Sánchez-Álvarez et al., 2020<br><br>(M)	Secondary Students	44 Studies<br><br>Academic Performance	$N = 19\ 861$		EI Ability / Academic Performance<br>$r = .31$<br>9 % var. explained	—

Articles	Age Range	Sampling Methodology	Sample Size Gifted Group	Sample Size Non-Gifted Group	Effect Size and % of Variance Explained	
Kong, 2014<br><br>(M)	—	46 Studies<br>- Intelligence Measures<br>- Admission Tests	- Intelligence Measures: *N* = 7 945<br>- Admission Tests: *N* = 3039		Focus on Intelligence Measures<br>- EI Ability Measures / General Intelligence *r* corrected = .33 10.89 % var. explained<br>- EI Ability Measures / Verbal Intelligence *r* corrected = .26 6.76 % var. explained<br>- EI Ability Measures / Non-Verbal Intelligence *r* corrected = .27 7.29 % var. explained	The authors distinguished between test-based measures of intelligence and admission tests.
Sharifi & Sharifi, 2014	Grades 10 - 12	—	*n* = 60	*n* = 60	—	Unspecified methodology.<br><br>Only females participants.

Articles	Age Range	Sampling Methodology	Sample Size Gifted Group	Sample Size Non-Gifted Group	Effect Size and % of Variance Explained	Methodological Remarks
Zeidner et al., 2005	Grades 7 - 10	Gifted Program<br>- Academic achievement<br>- Cognitive Ability Assessment	*n* = 83	*n* = 125	- EI Ability Measures / Group<br>*d* = .39, *p* < .05<br>3.65 % var. explained<br><br>- EI Trait Measures / Group<br>*d* = - .57, *p* < .05<br>7.51 % var. explained<br><br>- EI Ability Measures / Verbal Competencies<br>*r* = .32, *p* < .01<br>10.24 % var. explained<br><br>- EI Trait Measures / Verbal Competencies<br>*r* = -.21, *p* < .01<br>4.41 % var. explained	Participants' vocabulary levels were check.
Woitaszewski & Aalsma, 2004	Grade 11 and 12	Gifted Program	*n* = 39	Normative data	- EI Ability Measures / Cognitive Abilities<br>*r* = - .03<br>0.09 % var. explained<br><br>- EI Ability Measures / Academic Achievement<br>*r* = .37, *p* < .05<br>13.69 % var. explained	Wide heterogeneity in GPA scores within the gifted group.<br><br>No control group.

Articles	Age Range	- Sampling Methodology	Sample Size Gifted Group	Sample Size Non-Gifted Group	Effect Size and % of Variance Explained	Methodological Remarks
Mayer et al., 2001	13 - 17 years	- Summer Program for Gifted<br>- Relatives to one author	*N* = 11	—	—	Qualitative study with no control group.<br>Only verbal intelligence has been assessed.

Note. __ indicates that the information is not available in the article; (M) indicates that the study is a meta-analysis.

Table S17

Summary table of methodological aspects and effect sizes from articles on over-excitabilities.

Articles	Age Range	Sampling Methodology	Sample Size Gifted Group	Sample Size Non-Gifted Group	Effect Size and % of Variance Explained	Methodological Remarks
Winkler & Voight, 2016<br><br>(M)	—	12 Studies<br><br>Intellectual Giftedness Only	*N* from<br><br>79 to 486		Group / Type of OE<br>Overall Weighted Mean Effect Size = .17, n.s<br>0.72 % var. explained<br>To<br>Overall Weighted Mean ES = .55, $p < .05$<br>7.02 % var. explained<br>1.19 % Median var. explained	All studies included control groups.
Alias et al., 2013	10 - 15 years	Summer Program for the Gifted	$n = 335$	—	—	No control Group.<br>No data analyses.
Rinn et al., 2010	11 - 16 years	Summer Program for the Gifted<br>IQ ≥ 125	$n = 379$	—	—	No control group.
Gross et al., 2007	Grades 6 - 10	Summer Program for the Gifted<br>IQ ≥ 125	$n = 248$	—	Grade Level / Type of OE<br>$r = - .03$, n.s<br>0.09 % var. explained<br>To<br>$r = .22$, $p < .01$<br>4.84 % var. explained<br>1 % Median var. explained	No control group.

Articles	Age Range	Sampling Methodology	Sample Size Gifted Group	Sample Size Non-Gifted Group	Effect Size and % of Variance Explained	Methodological Remarks
Tieso, 2007	7 - 15 years	Academic Achievement	$n = 296$	$n = 184$	Gender / OE subscales<br>- Gifted Group<br>Partial $\eta^2 = .19$, $p < .001$<br>19 % var. explained<br>- Non-Gifted Group<br>Partial $\eta^2 = .24$, $p < .001$<br>24 % var. explained	—
Bouchard, 2004	4 - 12 years	—	$n = 96$	$n = 75$	- Psychomotor OE Score / Group<br>$\eta^2 = .037$, $p = .012$<br>3.67 % var. explained<br>- Sensual OE Score / Group<br>$\eta^2 = .008$, $p = 247$<br>0.8 % var. explained<br>- Imaginational OE Score / Group<br>$\eta^2 = .011$, $p = .165$<br>1.1 % var. explained<br>- Intellectual OE Score / Group<br>$\eta^2 = .119$, $p < .01$<br>11.9 % var. explained<br>- Emotional OE Score / Group<br>$\eta^2 = .002$, $p = 546$<br>0.2 % var. explained	Unspecified methodology.

Articles	Age Range	Sampling Methodology	Sample Size Gifted Group	Sample Size Non-Gifted Group	Effect Size and % of Variance Explained	Methodological Remarks
Bouchet & Falk, 2001	College Students	- Gifted Program<br>- Advanced Placement Classes	*N* = 273<br>- Gifted Program: *n* = 142<br>- Advanced Placement: *n* = 131	*n* = 288	—	—

Note. __ indicates that the information is not available in the article; (M) indicates that the study is a meta-analysis.
